# The effects of rhythmic structure on tapping accuracy

**DOI:** 10.3758/s13414-023-02778-2

**Published:** 2023-10-10

**Authors:** Andrew J. Milne, Roger T. Dean, David Bulger

**Affiliations:** 1 The MARCS Institute for Brain, Behaviour and Development, Western Sydney University, Locked Bag 1797, Penrith, NSW 2751 Australia; 2 Department of Mathematics and Statistics, Macquarie University, Sydney, Australia

**Keywords:** Music cognition, Rhythm, Meter, Synchronized tapping

## Abstract

Prior investigations of simple rhythms in familiar time signatures have shown the importance of several mechanisms; notably, those related to metricization and grouping. But there has been limited study of complex rhythms, including those in unfamiliar time signatures, such as are found outside mainstream Western music. Here, we investigate how the structures of 91 rhythms with nonisochronous onsets (mostly complex, several in unfamiliar time signatures) influence the accuracy, velocity, and timing of taps made by participants attempting to synchronize with these onsets. The onsets were piano-tone *cues* sounded at a well-formed subset of isochronous cymbal *pulses*; the latter occurring every 234 ms. We modelled tapping at both the rhythm level and the pulse level; the latter provides insight into how rhythmic structure makes some cues easier to tap and why incorrect (uncued) taps may occur. In our models, we use a wide variety of quantifications of rhythmic features, several of which are novel and many of which are indicative of underlying mechanisms, strategies, or heuristics. The results show that, for these tricky rhythms, taps are disrupted by unfamiliar period lengths and are guided by crude encodings of each rhythm: the density of rhythmic cues, their circular mean and variance, and recognizing common small patterns and the approximate positions of groups of cues. These lossy encodings are often counterproductive for discriminating between cued and uncued pulses and are quite different to mechanisms—such as metricization and emphasizing group boundaries—thought to guide tapping behaviours in learned and familiar rhythms.

**Supplementary Information:**

The online version contains supplementary material available at 10.3758/s13414-023-02778-2.

Western music, including baroque/classical/romantic, jazz, rock, and pop, typically uses rhythms with isochronous accents grouped, hierarchically, in twos or threes. Isochronous and syncopated rhythms in familiar time signatures have been widely studied in perceptual and sensorimotor synchronization tasks (the latter typically assessed by tapping experiments). These have found that isochronous pulses are typically grouped into twos or threes (Bolton, 1894; Fraisse, 1982); nonisochronous taps are accented according to how they are grouped (the starts and ends of groups are often emphasized; Essens & Povel, 1985; Repp et al., 2005); taps usually occur a little early (Aschersleben, 2002); tapped ratios between long and short interonset intervals are distorted (Fraisse, 1946; Repp et al., 2012); long interonset intervals are tapped more accurately when they are audibly subdivided (Repp, 2003); and mechanisms related to adaptation (period and phase correction) and anticipation (extrapolation and perseveration) are used by performers to synchronize with each other and with gradually changing tempos (Harry & Keller, 2019).

However, outside the broad generalities of the above musical genres, and in much non-Western music, more complex nonisochronous rhythms are common and aesthetically prized. For example, the rhythmic emphases in Balkan *aksak* music often form extended repeating patterns of “long” (*L*) and “short” (*s*) interonset intervals like the 3-onset *Lss*, approximated by (3 2 2) 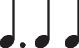 (the numbers represent the number of eighth-notes or quavers, although, in performance, the ratios between the differently sized IOIs may not be precisely equivalent to low-integer ratios; Bernacki, 2022; Bonini-Baraldi et al., 2016; Goldberg, 2015), or the 5-onset *ssLss* approximated by (2 2 3 2 2) 
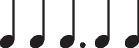
—see Fracile (2003) and Moelants (2006) for numerous other examples. In both these cases, the *period* of the rhythm (the number of isochronous pulses in the rhythmic *cycle* of repetition) are prime numbers: 7 and 11, respectively, which cannot be factorized by either 2 or 3. High-prime rhythms like these are common in *aksak* whilst being comparatively rare in Western music. As noted by Toussaint (2002, 2003), nonisochronous bell patterns are also characteristic of music originating from West Africa; one of the most notable of these being the *Bembé*, which has a form analogous to the equally tempered diatonic scale: (2 2 1 2 2 2 1) 
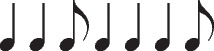
 (7 onsets, 12 pulses; Pressing, 1983); Polak (2010) identifies a number of distinct nonisochronous patterns used by Malian jembe drummers. Clayton (2020) provides a detailed analysis of how, in North Indian music, nonisochronous structures can pertain to even the deepest (slowest) metrical levels.

Furthermore, within the Western tradition, nonisochronous metrical or hypermetrical structures are a vital stylistic feature of jazz fusion, progressive and post-rock as exemplified by the (3 4 3) 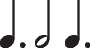
(3 onsets, 10 pulses) pattern in Mahavishnu Orchestra’s “The Dance of Maya” (McLaughlin, 1971); the (3 3 2 2) 
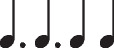
 (4 onsets, 10 pulses) pattern in King Crimson’s “Larks’ Tongues in Aspic, Part Two” (Fripp, 1973); the main (3 3 3 1 2 2 2 3) riff (8 onsets, 19 pulses) in Van der Graaf Generator’s “Meurglys III” (Hammill, 1976); the (6 4 5 3 3 2 7 3 7 3 4 8) cycle (of 12 onsets, 55 pulses) running through much of Henry Cow’s “Ruins” (Frith, 1974); and the widely discussed rhythm in Radiohead’s “Pyramid Song” (Yorke et al., 2001), which can be approximated by (5 4 6 5 4; 5 onsets, 24 pulses; Hesselink, 2013; Osborn, 2017).

Uneven rhythms with many onsets per cycle, such as these, raise many interesting questions related to how they affect tapping and metrical perception, and what mechanisms or strategies people use to learn their structure. With the exception of fascinating early work (Essens & Povel, 1985; Povel & Essens, 1985) and our own two recent studies (Bulger et al., 2022; Dean et al., 2021), which used rhythms with up to 11 onsets per cycle, there has been almost no experimental testing and modelling of tapping to uneven rhythms with many onsets per cycle; for instance, the synchronization experiments conducted by Repp et al. (2005, 2011, 2012, 2013), Snyder et al. (2006), and Polak et al. (2018) all used uneven rhythms with no more than three onsets per cycle.

As reported in the aforementioned Essens and Povel (1985), participants made *continuation taps* (replicating the previously heard rhythm) to 24 different cyclic rhythms with 7, 8, 10, and 11 onsets with two distinct IOIs. The short IOI was always 250 ms, the longer IOI varied from 375 to 1,000 ms. Participants typically lengthened the longer IOI, extending similar findings for spontaneous tapping experiments (Fraisse, 1946). In another tap-continuation experiment with rhythms comprising up to 9 onsets, timings were more accurate for “metrical” rhythms with 16-pulse periods than for “nonmetrical” rhythms with 10-, 11-, 13-, and 14-pulse periods. This suggests that high-prime periods (e.g., those with a prime factor higher than at least 2) are easier to tap because they can be metricized. There were no rhythms tested that had periods with a prime factor of 3 (e.g., 3, 6, 9, 12).

In Povel and Essens (1985), participants gave *continuation taps* (replicating the previously heard rhythm) to 35 different 9-onset rhythms in 15-pulse periods (cyclic permutations of (1 1 1 1 2 2 3 4), where 1 is 200 ms). Under their assumption that the starts and ends of groups of three or more onsets, the second onset of groups of two onsets, and isolated onsets, are perceptually accented, rhythms whose onsets (particularly the accented onsets) that more closely align with a putative isochronous pulse were tapped more accurately. For the more difficult rhythms, they note participants “reported that they used various mnemonics such as assigning numbers to successive tones” (Povel & Essens, 1985, p. 424). A subset of the above rhythms was accompanied by a low-frequency pulse that was well-matched to the rhythms; participants tapped more accurately with this pulse than without. In a third experiment, nonisochronous rhythms with a 12-pulse period were accompanied by a low-frequency isochronous pulse with an IOI of either 3 or 4 pulses. Participants typically rated the version where the pulse better aligned with the rhythm as simpler than the other version. These findings provide evidence for their hierarchical clock theory, which suggests that metricization of rhythms: onsets are perceived or tapped in relation to a subjective isochronous beat, and this subjective beat provides an efficient coding of the rhythmic structure (at least for rhythms that can be reasonably matched to at least one feasible beat). These findings, in part, rely on the assumption that the ends of groups of 2 onsets, the starts and ends of groups of 3 or more onsets, and isolated onsets, are perceptually accented. There is experimental evidence for the accenting of the second tone in 2-onset groups, although it is worth noting these studies had only three participants (Povel & Okkerman, 1981); of the other types accents, Povel and Essens (1985) refer only to unpublished pilot experiments.

Our above-mentioned articles (Bulger et al., 2022; Dean et al., 2021) analyzed participant tap-times and velocities to 91 uneven rhythms, each repeated for 30 seconds. Every rhythm comprised regular *pulses* (234 ms IOI, 256 BPM) sounded by a cymbal. Note that we define “pulses” as referring only to isochronous physical sonic events, not to a salient subjective metrical percept of a listener or performer; for the latter, we use the term *beat*. An uneven well-formed (Carey & Clampitt, 1989) subset of these pulses were also sounded by a piano tone to serve as *cues* to tap. Participants were asked to tap only to the *cued pulses*, and to tap as accurately as possible; once the rhythm started playing, they could start tapping as soon as they felt ready, and until the rhythmic repeats ended. In each trial, the first cue played in the rhythm was randomized so, across all trials, there was no favoured “rotation” of the rhythm. The 91 rhythms varied in their complexity: some were relatively straightforward to tap along with; many were extremely tricky, even after several repetitions. In the present article, we analyze the same raw dataset but approach it with different research questions and modelling approach.

The time-series analyses undertaken in Dean et al. (2021) shows overall poor performance compared with familiar rhythms in the literature, and progressive learning of most but not all rhythms. It also suggests the influences of a range of rhythmic features. The innovative point-process method used in Bulger et al. (2022) provides a continuous-time model of the propensity to tap and its dependence on recent cues and participants’ prior taps: learning of the rhythmic patterns generally improves over each performance, with more accurate learning for simpler or more familiar rhythms, but participants may rely on heuristics to approximate imperfectly learned rhythms.

Here, our aims are to use these same data to establish ways *rhythmic structure*—the sequence of interonset intervals in one cycle of a repeating rhythm—influence the following aspects of tapping behaviour (see Methods for more formal definitions):

- for each rhythm, the *overall accuracy of taps* (a summary measure of how frequently taps occur in the vicinity of cued pulses weighted by their temporal accuracy)
- for each cued pulse in a rhythmic structure, the *probability of a correct tap*; for each uncued pulse in a rhythmic structure, the *probability of an incorrect tap*
- for each tapped pulse in a rhythmic structure, the *velocity of a correct tap* and the *velocity of an incorrect tap*
- for each tapped pulse in a rhythmic structure, the *(signed) temporal asynchrony of a correct tap* and the *(signed) temporal asynchrony of an incorrect tap*.

The measure in the first of these four aspects allows us to assess ways in which rhythmic structure influences the rhythm’s overall learnability and playability; for instance, we may find that rhythms with cycles of 7 pulses (or another number not divisible by 2 or 3) are tapped less accurately than those that with cycles of 8 pulses (or another number divisible by 2 or 3). Such findings provide clues as to strategies and heuristics used by performers to learn and play sometimes difficult and unfamiliar rhythms. For example, only rhythms divisible by two or three can be mentally counted, in every cycle, as “1 & 2 & …” or “1 & a 2 & a …,” which aligns with the apparently natural tendency to *subjectively metricize* isochronous pulses in twos or threes (Bolton, 1894; Fraisse, 1982), also supported by Essens and Povel (1985) and Povel and Essens (1985).

The measures corresponding to the latter three aspects operate at the pulse level and so also give insight into temporal expectation: where in the rhythmic structure is expectation sufficiently strong to ensure correct and decisive (high velocity) taps; where in the rhythm might expectation be mistakenly focussed resulting in incorrect taps on uncued pulses, or taps that are systematically mistimed? In other words, how does rhythmic structure influence temporal expectation? For example, we might find that the first cue after a long gap is typically tapped with greater certainty or velocity than is a cue after a short gap, as would be predicted by the theory of metrical accenting developed by Povel and Essens (1985).

In this article, we test many different mathematical characterizations of rhythmic structures; several of these characterizations vary by rhythm, several vary by pulse (given a rhythmic structure). For example, characterizations of a full rhythm include things like the number of cues it contains, the number of pulses in its cycle, the balance, evenness, and IOI entropy of the rhythm (Milne & Herff, 2020). Characterizations of pulses within a given rhythmic structure include things like the distance of the pulse from the rhythm’s circular mean, *n*-gram Markov probabilities and other probabilities based on pattern continuation, edge-detection, all of which provide different values for different pulses (cued and uncued) as a function of the rhythmic structure of the cues.

In our models, we test whether these rhythm-level and pulse-level characterizations can predict the above four measures of tapping behaviour. Some of the predictors are correlated, and some indicate similar underlying mechanisms or strategies; we perform variable selection (using projective prediction; Piironen et al., 2020) to select the most useful set of predictors. Many of these predictors are novel, and many are also suggestive of possible underlying strategies, or perceptual and cognitive mechanisms that may underlie tapping behaviour. These may be conscious or learned strategies such as mental counting and grouping in twos or threes, which allows the rhythmic structure to be mentally chunked, thereby supporting short-term memory; unconsciously derived statistical summaries that reduce cognitive load but only partially represent the full rhythmic structure hence may lead to errors; rotational asymmetries that aid position-finding within the rhythm, such as occurs in uneven musical scales (Pelofi & Farbood, 2021); or the presence of short repeating rhythmic figures that can be easily learned. Here, we do not formally test any of these putative underlying strategies or perceptual and cognitive mechanisms and, due to the large number of predictors we use here, our work also has an exploratory component. However, the results may indicate fruitful directions for experiments focussed on substantiating precise mechanisms. All the predictors are summarized in Methods and some of the more complex ones are defined in a formal mathematical manner in the Supplementary (10.3758/s13414-023-02778-2).

## Methods

### Participants

One hundred and eleven first-year psychology university students recruited from Western Sydney University participated in the experiment. One participant did not provide demographic information; the following summaries are for the remaining 110 participants. There were 89 female and 21 male participants, their mean age was 22.4 (in the range 18 to 48, with a standard deviation of 6.2). Their mean Ollen musical sophistication index (Ollen, 2006) was 86 (in the range 10.5 to 873, with a standard deviation of 113.3, with 2 participants scoring higher than 500, the value of 500 is considered the transition between lower and higher sophistication). Their mean number of years of musical practice was 0.8 (in the range 0 to 13, with 85 participants having had no practice, and 6 having had more than 5 years regular practice). As Australian residents, these participants are enculturated to Western music.

### Materials

Participants tapped on a Roland Handsonic HPD20 (the Handsonic has multiple adjacent pads and participants were told they could tap on whichever pad was most comfortable for them). Each tap produced a piano tone at F above middle C. Each rhythm played for 30 seconds (129 pulses). The rhythmic loops (as exemplified in Table 1) comprised isochronous pulses each sounded with a cymbal (IOI 234  ms, 256  bpm), and cues sounded with a piano tone (middle C) on some pulses. The audio from the cues was subject to the same audio buffer latency (128 samples, ~3 ms) as was the audio coming from the tap: both MIDI streams were routed via Max 7 (Cycling74, 2021) into a standalone version of the software synthesizer Pianoteq 5 (Modartt, 2021), which was installed on the same computer and produced the piano sounds.

**Table 1:** Three well-formed rhythms The first rhythm has 8 pulses, 5 cues (*N* = 8, *K* = 5) with indicator vector (1 0 1 0 1 1 0 1); the second has 11 pulses, 5 cues (*N* = 11, *K* = 5) with indicator vector (1 0 0 1 0 0 1 1 0 0 1); the third has 12 pulses, 7 cues (*N* = 12, *K* = 7) with indicator vector (1 0 1 0 1 0 1 1 0 1 0 1)

*n*	0	1	2	3	4	5	6	7				
Pulses (cymbal)	×	×	×	×	×	×	×	×				
Cues (piano)	×		×		×	×		×				
*n*	0	1	2	3	4	5	6	7	8	9	10	
Pulses (cymbal)	×	×	×	×	×	×	×	×	×	×	×	
Cues (piano)	×			×			×	×			×	
*n*	0	1	2	3	4	5	6	7	8	9	10	11
Pulses (cymbal)	×	×	×	×	×	×	×	×	×	×	×	×
Cues (piano)	×		×		×		×	×		×		×

Because they are well-formed, the long and short IOIs are distributed as evenly as possible. Every pulse in every rhythm is uniquely identified by the number *n*, which counts upwards from 0 to *N* − 1, and 0 is aligned to the first cue when the rhythm is written such that the small IOIs are as close to the end as possible

The 91 rhythms—patterns of cues—were all possible nondegenerate well-formed rhythms (Carey & Clampitt, 1989) with between 2 and 11 cues that are subsets of 3 to 13 pulses and have no interonset intervals larger than 5 pulses; they are listed in the Supplementary. Well-formed rhythms are a useful class of rhythms because they allow a wide variety of multi-onset patterns with just two *distinct* interonset intervals, which seems to be a common feature of many non-Western rhythms (e.g., the Euclidean/maximally even rhythms identified by Toussaint, 2002), have interesting compositional possibilities (Milne, 2018; Milne & Dean, 2016), and are defined for cycles of any length (such as the familiar 3-, 4-, 6-, 8-, 9-, and 12-pulse cycles, and the less familiar 5-, 7-, 10-, 11-, and 13-pulse cycles). The pulse IOI is in the range of typical tempos in pop music and corresponds to a quaver (1/8th note) at a tempo of approximately 128 beats per minute (as would often be sounded by a high-hat pulse). This also means that cues on consecutive pulses are within Povel’s 250- ms grouping limit, while all other pairs of cues are substantially outside it. Pulses were sounded to make the often very difficult tapping task a little easier—sounded pulses offer the “subdivision benefit” (Repp & Su, 2013), which aids predicting the timing of cues comprising several pulses. Each rhythm was started on a randomly chosen cue; this was done to allow the effect of the first-heard cue (i.e., the rotation or phase of the rhythm) to be randomized out when aggregated across observations (as in the perceptual investigations conducted by Parncutt, 1994). Examples of three different well-formed rhythmic loops are shown in Table 1.

### Procedure

The tapping task reported in this article occurred in Blocks 1, 3, 5, and 7 of an 8-block experiment. Participants listened to the rhythm and were encouraged to start tapping as accurately as possible as soon as they felt ready (their written instructions defined an “accurate performance” as “one where you tap only where a piano sound occurs, and your taps are closely synchronized with the piano.” The 91 rhythms were divided into four disjoint sets of sizes 23, 23, 23, and 22, and each participant played every rhythm from one set in random order over two separate blocks (1 and 3). That participant then played the same set of rhythms again, also in two blocks (5 and 7) and in random order. In a very few cases, due to procedural errors, a rhythm was tapped 1 or 3 times instead of the intended 2. Different tasks—data from which are not reported in this paper—were undertaken in the even-numbered blocks. The tasks were interleaved to provide sufficient variety to maintain participants’ interest over the course of the experiment.

This study was not preregistered. Experimental data, code, and fitted models are available at https://osf.io/b8ad2/.

### Design

#### Dependent variables (rhythm-level)

##### Rhythm-level accuracy

For each 30-second performance of a rhythm, *tap* _ *acc* is a score summarizing the timing accuracy of taps (relative to the cues) as well as the correctness of the tapping (i.e., correctly tapping on cued pulses and not tapping on uncued pulses). It is calculated as the cosine similarity between a smoothed indicator vector of the taps and a smoothed indicator vector of the cues, both with a temporal resolution of 1  ms. Gaussian smoothing of 10- ms standard deviation is used because this loosely approximates the temporal just noticeable difference for isochronous rhythms (Friberg & Sundberg, 1995). We use this quantification, rather than mean asynchrony or the standard deviation of asynchronies, because it penalizes—in a principled way—“incorrect” taps that occur when there is no cue and missing taps when there is a cue; incorrect taps or missing taps are ignored when calculating asynchronies because of the requirement for a one-to-one pairing between every tap and every cue. This measure penalizes taps that occur early or late relative to a cue. This means it penalizes constant asynchrony; for example, a performance where every tap is exactly 30  ms before its cue will have lower accuracy than one where every tap is 15 ms before the cue. Our code allows for the effect of constant asynchrony to be factored out; however, we chose not to do so because constant asynchronies (e.g., negative mean asynchrony) are still inaccuracies; for example, professional drummers typically have lower negative mean asynchrony, sometimes approaching zero (Krause et al., 2010). Furthermore, for our data, the correlation between accuracy values achieved when factoring out constant offsets versus not factoring them out is 0.89; so, here, the two measures are quite similar. For reference, the standard deviation of tap-cue asynchronies in these data are reported in Dean et al. (2021). The *tap* _ *acc* variable lies in the unit interval, hence, is treated as beta distributed (conditional on the predictors). This novel method for calculating tap accuracy, and its useful properties, are detailed in the Supplementary.

#### Dependent variables (pulse-level)

For all of the pulse-level dependent variables, a tap is deemed to have occurred at any given pulse if it falls into the 234-ms window stretching from halfway towards the previous pulse and halfway towards the next pulse. Where a window includes more than one tap, the tap with the most accurate timing is kept and all other taps discarded. Out of a total of 314,671 taps, 10,768 such discards were made (3.4% of the total), leaving 303,903 taps in a total of 641,775 pulse-level observations. Many of these are the result of “double taps,” where the second tap follows the first by about 15 or 60 ms, presumably due to the finger bouncing back from the surface of the drum while the finger is still being directed towards the drum surface (Bulger et al., 2022). As detailed above, every pulse in each rhythm is uniquely identified by a number 0, 1, …, *N* − 1, where the pulse assigned the value of 0 is defined by the rhythmic structure, not by the first played cue. All pulse-level models use a binary variable that indicates whether or not the pulse is cued; this allows us to calculate tap probabilities, velocities, and timing errors separately for cued (correct) and uncued (incorrect) pulses.

##### Pulse-level tap probability

This dependent variable is the tap probability for every pulse in every performance of every rhythm (48,282 observations). The probability is derived from *tap* _ *num*, which is the number of times a participant tapped each of a rhythm’s *N* different pulses during each 30-second performance, and *n* _ *pulses*, which is the number of times each pulse occurs over those 30 seconds. For example, in any performance of the rhythm (2 2 1 2 1), which can also be written as an indicator vector (1 0 1 0 1 1 0 1), each of the 8 pulses (each lasting 234  ms) will occur 16 times over the 30 seconds (because 30/(8 × 0.234) ≈ 16.03), and there may be anything from 0 to 16 taps within the window centred on each pulse. Hence, this is a binomial-like variable (it is theoretically possible for each pulse to be separately modelled as a Bernoulli variable, which would have allowed for learning to be modelled within the duration of each performance, but this was not computationally feasible).

##### Pulse-level tap velocity

The dependent variable *tap* _ *vel* is the velocity of the tap for every tapped pulse (cued and uncued) in every performance of every rhythm (303,903 observations). All untapped observations are removed in order to allow for predictors’ effects on tap probability and tap velocity to be disambiguated. (A model using some form of zero-inflation would be an alternative way of disambiguating these two aspects but was not computationally feasible.) The MIDI value for velocity is a 7-bit integer—hence, nonzero velocities are integers from 1 to 127. The upper value is best considered as being *censored* because any tap with a velocity greater than 127 MIDI units is still recorded as 127. A histogram of the tap velocities showed a substantial peak in the number of these maximum velocity values (see the Supplementary). There were few values close to the minimum velocity of 1 MIDI unit. For these reasons, tap velocities were modelled as normally distributed (conditional on the predictors) with upper censoring. In the model, this variable was standardized (divided by its standard deviation) but not mean-centred.

The process used for variable selection (detailed later In Methods) is computationally intensive so, for this purpose only, the *tap* _ *vel* for each tapped pulse was averaged within each performance, and modelled as normally distributed (conditional on the predictors), without censoring.

##### Pulse-level tap-asynchrony

The dependent variable *tap* _ *delta* is the signed time difference between each tap and its closest pulse for every tapped pulse in every performance of every rhythm. The range of values in ms (i.e., prior to standardization) is, therefore, [−117, 117]. Both boundaries are *truncated* because values outside this range are not recorded in the data set: as detailed above, they are automatically assigned to the previous or following pulse or removed. For this reason, tap timing was modelled as normally distributed (conditional on the predictors) but truncated above and below (a truncated Student’s *t* distribution was also tried but performed less well under cross-validation). In the model, this variable was standardized (divided by its standard deviation) but not mean-centred. Unlike the tapping accuracy (*tap* _ *acc*) variable, the tap-timing error is signed (hence, sensitive to whether taps are late or early) and, because the timing error is measured with respect to the closest pulse—cued or uncued—it does not penalize incorrect taps. Also *tap* _ *acc* refers to an entire performance, whereas *tap* _ *delta* refers to each tap.

As with *tap* _ *vel*, for variable selection only, the *tap* _ *delta* for every tapped pulse was averaged within each performance and modelled as normally distributed (conditional on the predictors), without truncation.

### Predictors

Unless otherwise noted, all predictors were hypothesized prior to undertaking the experiment. In each case, we identify the hypothesized effect of each predictor on tapping behaviour, and the underlying strategies, or perceptual, cognitive, or motoric mechanisms or features they may indicate. Unless the mathematical specification is very simple, the predictors are described verbally in this section, and the formal mathematical definitions are provided in the Supplementary.

#### Performance-level predictors

##### Perf_num

The number of rhythm performances completed by the participant; this is a number between 1 and 44 or 46 (depending on which group of rhythms the participant was randomly assigned to). We expect this to be positively associated with tapping accuracy due to participants becoming more familiar with task and having had the opportunity to develop or select useful strategies. Hence, this predictor is indicative of the learning of overall strategies—*“meaningful” learning* (Mayer, 2002).

##### Repetition

The current number of performances (including the current performance) of each specific rhythm. Most rhythms were played twice by each participant, but there were a few instances of a participant having played a rhythm 1 or 3 times at the end of the experiment; hence, this predictor has values 1, 2, or 3. We expect this to be positively associated with tapping accuracy due to participants having gained greater familiarity with each specific rhythm with each successive performance of it. Hence, this predictor is indicative of *rote learning*.

#### Rhythm-level predictors

##### N

The number of pulses in each rhythmic cycle. We expect rhythms with higher *N* to be less accurately tapped because the duration of the rhythm’s cycle of repetition is longer and hence subject to greater memory decay. Furthermore, such rhythms repeat fewer times over each 30-second performance, which reduces the opportunity for rote learning of the pattern. Hence, this predictor is indicative of *short-term memory decay and rote learning*.

##### K

The number of cues or IOIs in each rhythmic cycle. We expect rhythms with higher *K* to be tapped less accurately because they require a longer sequence of IOIs to be learned. Hence, this predictor is indicative of *short-term memory capacity*.

##### Mean_IOI

The mean IOI between cues, which is given by *N*/*K*. This predictor was thought of after the initial data analysis, when it became apparent that it might play a useful predictive role. This predictor may be positively associated with tapping accuracy because the speed of information flow (the IOI sequence) in the rhythm is slower as it increases, in which case this predictor is indicative of *speed constraints on cognitive processing*. Rhythms with lower *mean* _ *IOI* have events occurring more frequently (across time, they have higher cue density) and so may encourage tapping regardless of correctness; in this case, this predictor is indicative of a very reductive *statistical summary of the rhythm* (its cue density). Cues separated by many pulses are less likely to induce a beat (beats of about 500 ms are easiest to induce), which may be detrimental, in which case this predictor is also indicative of *metricization*.

##### Evenness

Evenness quantifies the extent to which interonset intervals are of a similar size (Amiot, 2009; Milne et al., 2015, 2017). See the Supplementary for a formal mathematical specification. Rhythms with low evenness will, therefore, tend to have *groups* (cues clustered in time) and *gaps* (uncued pulses clustered in time). For example, consider the rhythms (1 1 0) and (1 1 0 0) with respective evennesses of 0.866 and 0.707; the latter is more uneven than the former and the contrast between the short and long IOIs are greater. As such, more uneven rhythms have more discriminable rotational asymmetries, which can facilitate *position-finding*—the ability to estimate where one is within the rhythm. The concept of position finding is more commonly applied to musical scales (Balzano, 1982; Browne, 1981) where it has been recently demonstrated that asymmetries facilitate position-finding (Pelofi & Farbood, 2021). For these reasons, we expect rhythms with higher evenness to be harder to tap. (It is interesting to note that if the task does not require position-finding, symmetries can be beneficial because working memory can make use of redundancies to reduce information content; Attneave, 1955). This predictor is, therefore, indicative of *position-finding*.

##### IOI_ent

*Interonset interval entropy* quantifies the unpredictability of the distribution of interonset intervals in the rhythm, and has previously been found useful for predicting the recognizability and liking of rhythms (Milne & Herff, 2020). In a rhythm with relatively low entropy (1 0 0 1 0 0 1 0 0 1 0), all but one IOI is of size 3 pulses, which means a performer can be fairly confident that a guess of 3 will be correct; conversely, in the higher entropy (1 0 0 1 0), half the IOIs are of length 3 and half of length 2 so a guess of length 3 is less likely to be correct. Hence, we expect rhythms with lower IOI entropy to be easier to tap (easier to guess). This predictor is, therefore, indicative of a *lossy compression of the rhythm* (the distribution of IOIs between consecutive cues, ignoring their order).

##### Int_ent

*Interval entropy* is conceptually similar to IOI entropy (*IOI* _ *ent*) but considers the distribution of all temporal intervals between cues in the rhythm, modulo the period (the length of the cycle in pulses)—not just those between consecutive cues. Being a different way of characterizing rhythmic entropy, this predictor is similarly plausible to the already proven *IOI* _ *ent*. In the relatively low entropy rhythm (1 0 0 1 0 0 1 0 0 1 0) there is one IOI of 2 pulses (between consecutive cues), three IOIs of 3 pulses (between consecutive cues), two IOI of 5 pulses (between nonconsecutive cues), two of 6 pulses (between nonconsecutive cues), three of 8 pulses (between nonconsecutive cues), one of 9 pulses (between nonconsecutive cues). Hence, in this rhythm, it would be a better guess to tap 8 pulses, rather than 9, after any cue. For the same reasons as with IOI entropy, we expect rhythms with lower interval entropy to be easier to tap. Like *IOI* _ *ent*, this predictor is, therefore, indicative of a *lossy compression of the rhythm* (the distribution of IOIs between all cues in one cycle, ignoring their order).

##### CQ

The *coherence quotient*, developed by Carey (2002, 2007) for musical scales, quantifies the extent to which knowledge of the *generic interval* between any two cues provides information about their *intercue interval*. The generic interval between any two cues is the number of cues between them plus one; this is different to their intercue interval, which is the number of pulses between them plus one. *CQ* quantifies the number of *coherence failures* in the rhythm: a coherence failure occurs whenever a generic interval has a larger IOI than a larger generic interval. For example, in (1 0 0 0 0 1 0 1 0 1 0), for the first two cues, their generic interval is 1 and their intercue interval is 5; for the second and last cues, their generic interval is 2 and their intercue interval is 4; so we have a coherence failure. The coherence quotient divides the number of these coherence failures by the maximum possible number of coherence failures for a rhythm with *K* onsets and subtracts from this from 1; hence, a maximally coherent pattern has a *CQ* of 1. For a high-*CQ* rhythm like this, counting just the cues gives partial information about their intercue intervals (because their size is bounded below and/or above by the next smaller and/or larger generic interval’s size). For any rhythm where *K* < *N*, this represents a data compression because there are $\left(\genfrac{}{}{0pt}{}{K}{2}\right)$ generic intervals and $\left(\genfrac{}{}{0pt}{}{N}{2}\right)$ intercue intervals; but, even for maximal-*CQ* rhythms, this is a lossy compression because the precise intercue intervals are not described, just their ranking. As *CQ* decreases, this heuristic would become less effective. This predictor, therefore, is indicative of a *lossy compression of the rhythm* that may serve as a heuristic that is useful in high-*CQ* rhythms.

##### SQ

The *sameness quotient* (Carey, 2002, 2007) is also derived from relationships between generic intervals and intercue intervals. In a rhythm with a high sameness quotient more generic intervals have fewer different inter-cue intervals. In this sense, it is conceptually related to *interval entropy,* but it is simpler in that it only counts the number of different such intervals rather than their precise distribution. As with *CQ*, it is normalized by dividing the number of different intervals for every generic interval by the maximum possible such number for a rhythm with *K* onsets and subtracts from this from 1. Because all nondegenerate WF rhythms (as used in our experiment) have two inter-cue intervals per generic interval (all nondegenerate WF scales have Myhill’s property; Carey & Clampitt, 1989), for our rhythms, this predictor is more highly correlated with *K* (0.80) than it is with *IOI* _ *ent* (−0.71). The partial information provided by high-SQ rhythms provides partial information about intercue interval distributions—hence, cue positions in rhythms with higher *SQ* should be easier to guess than rhythms with lower *SQ*. This predictor is, therefore, indicative of a *lossy compression of the rhythm* that may serve as a heuristic that is useful in high-*SQ* rhythms.

##### Balance

For a rhythm represented as weighted points on a unit circle, this is the smallest distance of the rhythm’s centre-of-mass from the circumference (equivalently one minus its distance from the centre of the circle). Balance is equivalent to the circular (directional) variance of the rhythm (Milne et al., 2015, 2017). The more unbalanced the rhythm, the more informative of the cues’ locations is their circular mean; for instance, a rhythm where all onsets are clustered is highly unbalanced because all its onsets are close to their circular mean. In an unbalanced rhythm like this, tapping in the vicinity of the circular mean will likely result in correct taps; hence, balance indicates how useful the circular mean is as a lossy descriptor of the rhythm, and how easy that circular mean is to estimate. Furthermore, unbalanced rhythms are more distinctly asymmetric with respect to rotation, which may facilitate position-finding. This predictor is, therefore, indicative of a *lossy compression of a rhythm* using its circular mean and variance and, furthermore, of the extent to which tapping behaviour is influenced by *position-finding* (like evenness).

##### Duple_triple

This predictor is a simple way of quantifying how well a rhythm can be metrically coded; that is, chunked into familiar elements spaced by an isochronous beat. Isochronous rhythms without acoustic accents are subjectively metricized into twos or threes (Bolton, 1894), so this predictor simply codes whether or not *N* is divisible by either 2 or 3. Note that any rhythm with *N* that cannot be divided by 2 and cannot be divided by 3, could be reinterpreted as a syncopated rhythm of length 2*N* (or even 3*N*), which would mean this double-length rhythm can now be partitioned into pulses grouped into twos (or threes). But, clearly, this doubling (or tripling) in length represents an increase in the cognitive demands required (e.g., both *N* and *K* would be doubled or tripled). This predictor is, therefore, indicative of lossless *metrical coding*, which facilitates chunking to increase short-term memory capacity; it is also indicative of *familiarity* given the prevalence of duple and triple times in Western music (to which our participants are enculturated).

#### Pulse-level predictors

##### Cue

A binary indicator of whether a cue is present at each pulse in the cycle. This predictor represents an idealized performance—the higher its effect on taps occurring, the more cued pulses are tapped rather than uncued pulses. As detailed in the next subsection, in the models, this predictor interacts with every other pulse-level predictor hence allows separate estimation of the probabilities, velocities, and timing errors of correct (cued) and incorrect (uncued) taps and, hence, how well participants discriminate—with their taps—cued from uncued pulses.

##### APM

This predictor aims to quantify metrical accents arising from isochronous beats induced by rhythmic cues. It is the column-sum of a *circular* version of Eck’s *autocorrelation phase matrix*, which is used for *beat detection*—finding a regular beat that aligns with the rhythm and each pulse in that rhythm (Eck, 2006). It is also conceptually similar to Parncutt’s (1994) pulse-match salience. Pulses with higher *APM* values are likely in phase with a salient beat, so we expect them to more likely tapped, tapped harder, and possibly tapped earlier. Given metrical coding (as indicated by *duple* _ *triple*), this predictor is indicative of the use of *metricization and the chunking this facilitates* (where metrical weights arise from possibly gapped sequences of cues IOIs).

##### Seq_exp

*Sequential expectation* is a different way of quantifying metrical accents that may arise from isochronous beats induced by rhythmic cues. For all sequences of isochronous cues, the predictor assigns a higher value to the pulse that continues that isochronous sequence, such that the resulting value is greater for longer such sequences, and lower for sequences with larger IOIs. The nonlinear parameters that control these latter two aspects were separately optimized and are detailed in the Supplementary. As with *APM*, we would expect pulses with higher *seq* _ *exp* values to be more likely tapped, tapped harder, possibly tapped earlier. Given metrical coding (as indicated by *duple* _ *triple*), this predictor is indicative of the use of *metricization and the chunking this facilitates* (where metrical strength is derived from unbroken sequences of cue IOIs).

##### Edge

This predictor is concerned with the grouping structure of the rhythm; it finds the edges of groups (clusters of cues) and the edges of gaps (clusters of uncued pulses) by adapting standard edge-detection techniques for images (convolving with the derivative of a Gaussian kernel). In vision, edge detection is an important means of reducing information whilst preserving the overall the structure of an image. It is possible encoding just the temporal edges in rhythms may hold similar benefits. This technique is one way to generalize Povel’s accentual weights (which are high at the starts and ends of cue clusters) to also include uncued pulses, which is necessary because we are interested in both; for example, the bold entries in each of these rhythms show where edges are detected (i.e., the value of *edge* is high): (1 **1 0** 0 0 **0 1** 1 1), (0 **0 1** 1 1 **1 0** 0 0), (0 0 **0** 1 0 1 0 1 0 1 **0**), (1 1 **1** 0 1 0 1 0 1 0 **1**), (1 1 1 **1** 0 **1** 1 **1** 0 **1** 1), (0 0 0 **0** 1 **0** 0 **0** 1 **0** 0). Note how every second pattern is a “flipped” version (the complement) of the previous pattern (cues are replaced by silences, and vice versa), but this has no effect on the detected edge locations. Furthermore, with clusters comprising just two cues, we typically find the second cue has a higher edge value; for example, in the pattern (0 **0 1 1 0** 0), the respective edge values are (0.00, 0.22, 0.15, 0.27, 0.20, 0.00); it is interesting to note that the greater weight on the second cue of a 2-cue cluster corresponds with Povel’s suggested accent. Following Povel’s theory and perceptual experiments on accenting (Povel & Essens, 1985), we would expect high-*edge* pulses to be tapped with greater probability and with greater velocity. This predictor is indicative of a *lossy compression of the rhythm* (prioritizing the locations of perceptually prominent edges).

##### Mean_offset

The normalized mean temporal offset between the rhythm’s cues and the pulse of interest. The *mean* _ *offset* for each pulse (modulo the rhythm’s period) is the sum of the distances between that pulse and all cues following it (up to one period after) minus the sum of the distances between that pulse and all cues preceding it (up to one period before); hence, its units are pulses. Like *edge*, this predictor is also related to the grouping structure of the rhythm: pulses closer to the starts of cue clusters have low *mean* _ *offset*; pulses closer to the ends of cue clusters have high *mean* _ *offset*. It is, therefore, indicative of biases towards tapping behaviour at the starts or ends of groups, some of which may result from motoric considerations or from biases towards remembering the lengths of gaps compared with remembering the lengths of clusters: (a) taps later in a sequence of fast taps may become weaker due to fatigue (Repp et al., 2005); (b) cues at the starts of clusters may be harder to predict than those towards the end; (c) cues at the ends of groups may be tapped earlier than those at the start (Repp, 2003; Snyder et al., 2006). In combination with *edge*, this allows for a variety of different “contours” to be quantified over clusters.

##### Proj_cent

**Fig. 1:**
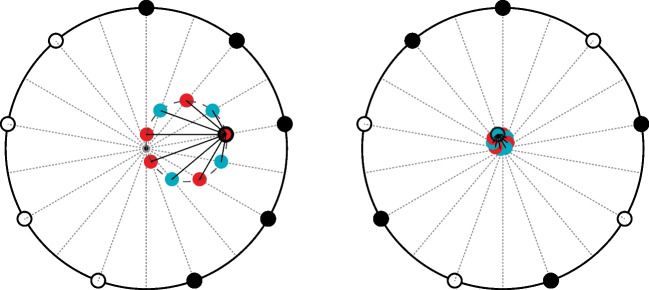
Two rhythms illustrated in circular format: cued pulses are depicted with black disks; uncued pulses with white disks. The first has lower balance than the second. A diameter (dashed) passes through every pulse in the rhythm and perpendicular projection lines (solid) are drawn from the rhythm’s centroid (coloured disk with black outline) until it intersects with each pulse’s diameter. Each intersection is depicted with a coloured disk centred on it. The distance of any intersection from the circle’s centre gives the magnitude of *proj* _ *cent* for that pulse; if the line meets the diameter on the side of the circle opposite to the pulse, it is negative (positive values are coloured red; negative are coloured blue). Note how the range of *proj* _ *cent* values is greater in the first low-balance rhythm than in the second high-balance rhythm; in a perfectly balanced rhythm (one whose centroid is at the circle’s centre), every pulse has a *proj* _ *pred* value of 0. For those familiar with audio recording, this is analogous to a figure-8 microphone polar pattern: the sound intensity produced is affected by its direction (pulse position) and the overall gain (imbalance); the sound’s phase is reversed by a 180-degree rotation. (Colour figure online)

The *projected centroid* is a pulse-level generalization of the rhythm-level *balance* predictor and represents how a simple statistical summary of a rhythm (its centroid) may influence predictive behaviour. Consider cued and uncued pulses as points on a circle, where angle denotes their time of occurrence. For any given pulse, *proj* _ *cent* is the signed magnitude of the rhythm’s centroid when projected onto the diameter passing through that pulse. This implies that pulses close to the circular mean of the cues have greater *proj* _ *cent* than pulses distant from the circular mean; the more unbalanced the rhythm (the lower its circular standard deviation) the greater the variation in these values. Its values are in the interval [−1, 1]. Figure 1 illustrates how *proj* _ *cent* is determined for two rhythms with differing balance levels. This predictor is indicative of a *lossy compression* using the rhythm’s centroid (i.e., its circular mean and its variance).

##### Markov2,3,4,...

The predictor *Markov*2 represents the optimal 2-step Markov predictor for the cue sequence. That is, at each pulse, we consider the length-2 binary sequence of cues or noncues over the previous two pulses. We then consider all occurrences of that cue sequence in the rhythm, and calculate the proportion that are followed by a cue. For instance, consider the tresillo rhythm, (1 0 0 1 0 0 1 0). It contains three distinct length-2 sequences: (0 0), (0 1) and (1 0). In this rhythm, the sequence (0 0) is always followed by 1, and the sequence (0 1) is always followed by 0, so the Markov predictor attaches probabilities of 1 and 0 to those following pulses, respectively. On the other hand, the sequence (1 0) occurs three times, followed by a 0 twice and a 1 once, so the optimal prediction after (1 0) would be a cue probability of 1/3. Altogether, therefore, the *Markov*2 cue probabilities for the tresillo rhythm are (1/3, 0, 1/3, 1, 0, 1/3, 1, 0). The predictors *Markov*3 and *Markov*4 are defined similarly for the optimal order-3 and order-4 Markov predictors. For our data, *Markov*2, *Markov*3, and *Markov*4 are highly correlated so it is not useful to include more than one in the same regression. Of the three predictors, *Markov*2 resulted in the model with the highest cross-validated likelihood; henceforth, we consider only *Markov*2. Our focus in this paper is on the structural features of rhythms, so we did not attempt Markov predictors that track probabilities over the course of the experiment, nor over a corpus of culturally familiar music (such as can be modelled within the IDyOM framework, Pearce, 2005, 2018). This predictor was introduced after the experiment had been performed. It is a probability, hence, lies in the interval [0, 1]. It is indicative of a mechanism derived from a *lossy compression* of a full rhythm (only the previous 2, 3, or 4 IOIs rather than all *K*).

##### Tap_lag1

For the tap velocity and timing models, this is a binary indicator for whether the previous pulse was tapped. For computational reasons (discussed earlier in Design), in the tap probability model, the data for each pulse (modulo the period) was averaged within in each 30-second performance. So, for this model, this predictor is the proportion of such taps occurring over each performance. Although not of primary interest in this investigation, it is included to help soak up residual autocorrelation and to allow the model to partially control for how past taps influence current taps.

#### Overview of the modelling approach

##### Bayesian multilevel regression

We used Bayesian multilevel (mixed effects) regression including as many random effects as computationally feasible (bear in mind that the dataset is very large—651,725 observations—and most of the models took many days to complete fitting). Maximal random effects structures are generally recommended to ensure uncertainties of estimated effect sizes are correctly estimated (Barr et al., 2013). In the first rhythm-level tapping accuracy model, all predictors’ effects were allowed to vary by participant and by rhythm as random effects; hence, the random effects’ structure was maximal. In the other models, it was unfeasible to include this many random effects due to the required runtime for the models. But it was possible to include the intercept and *cue* (the most important predictor) as random effects varying by participant and rhythm; that is, in their R-style formulas, these models included the terms ⋯ + (1 + *cue* | *participant*) + (1 + *cue* | *rhythm*); the complete formulas for all models are provided in Tables 2, 3, 5, and 6. In the Results section, we do not fully report the numerical values of the group-level (random) effects—these are available in the Supplementary.

**Table 2:**
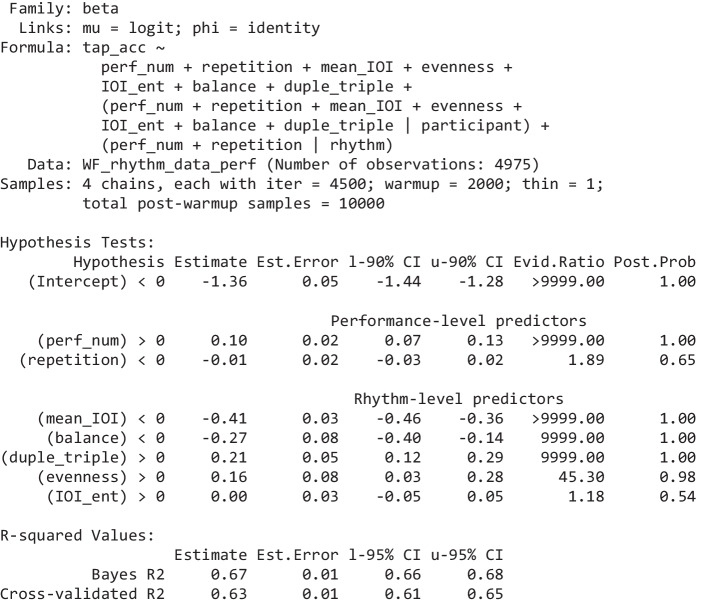
Rhythm-level tap accuracy model. The effects are grouped by level (performance, rhythm)

The “Hypothesis” column shows the hypothesis being tested, “Estimate” is the mean of the respective effect’s posterior distribution, “l-90% CI” and “u-90% CI” show the effect’s 90% credibility interval (a 95% CI is used for *R*^2^ at the bottom), “Evid.Ratio” are the odds of the hypothesis being true, “Post.Prob” shows the probability the hypothesis is true (hence, Evid.Ratio = Post.Prob/(1-Post.Prob)). We qualify an evidence ratio > 39 (a posterior probability > 0.975) as “strong” evidence. Full model summaries, including the group-level effects are provided in the Supplementary. The two forms of *R*-squared are calculated using the methods detailed in (Gelman et al., 2018); the cross-validated R-squared is calculated from the PSIS-LOO approximation of leave-one cross-validation (Vehtari et al., 2017)

**Table 3:**
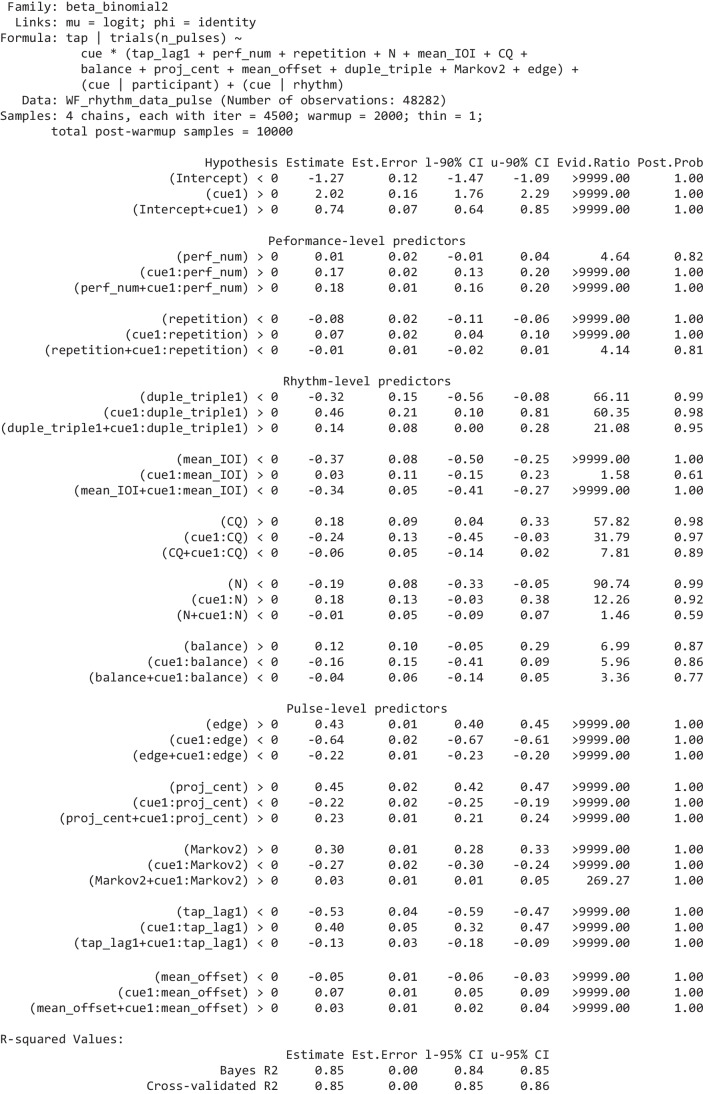
Summary of the model of pulse-level tap probability

The three-line groupings, for every predictor, show Bayesian hypothesis tests for three effects of interest: in the first line, the predictor’s effect on tapping bias (tapping incorrectly); in the second line, its effect on tapping discriminability; in the third line, its effect on tapping correctly (which is the sum of the previous two effects). The effects are grouped by level (performance, rhythm, pulse). The columns are the same as Table 2, as are the methods for calculating the *R*-squared values. Full model summaries, including the group-level effects are provided in the Supplementary

The regressions were performed using the brms package (Bürkner, 2017, 2018), which is a front-end for the Bayesian inference and MCMC sampler Stan (Carpenter et al., 2017). This means we can estimate the entire posterior probability distribution of each coefficient, so we do not report *p* values. Instead, we report the evidence ratios and posterior probabilities that each coefficient, or sums of coefficients that define a contrast of interest, is greater than (or less than) zero. We interpret evidence ratios greater than 39 (or less than 1/39) as strong evidence in favour of a directional hypothesis (this is loosely analogous to a one-sided *p* value of 0.025 or a two-sided *p* value of 0.05; Makowski et al., 2019; Marsman & Wagenmakers, 2017). All discrete regression inputs (*cue* and, in the tap velocity and timing models, *tap* _ *lag*1) were dummy (binary) coded; all continuous regression inputs were standardized to have a mean of 0 and a standard deviation of 1 with the exception of *tap* _ *lag*1 in the tap probability model, which was kept as a proportion between 0 and 1 to keep it on the same scale as in the tap velocity and timing models. All effects were given a prior with a Student’s *t*-distribution having a mean of 0, 3 degrees of freedom, and a scale of 1. For standardized regression inputs, this is a weakly informative prior which, centred at zero, helps to regularize the effect sizes towards plausibly small to medium values. The full model specifications are provided in Tables 2, 3, 5, and 6. Further information on how to interpret the coefficients of the models, given their distribution and link function, is provided in the Supplementary.

By interacting all pulse-level predictors with *cue*, we can assess how each predictor is associated with correct (cued) taps and incorrect (uncued) taps. When estimating tap probabilities, this means we can estimate the *discriminability* of cued and uncued pulse. In signal detection theory, “discriminability” (*d*-prime, also known as *sensitivity*) is the difference between the probit-transformed probabilities of a “hit” or “true positive” (here, a cued tap) and a “false alarm” or “false positive” (here, an uncued tap). For example, consider a simple model where a predictor *x* interacts with *cue* so that *y* (tap probability, velocity, or signed asynchrony) is predicted by *β*_0_ + *β*_1_*cue* + *β*_2_*x* + *β*_3_(*cue* × *x*) + *ϵ*. At an uncued pulse, *cue* = 0; at a cued pulse, *cue* = 1. Hence, the effect of *x* on uncued *y* is given by *β*_2_; the effect of *x* on cued *y* is given by *β*_2_ + *β*_3_; and the difference between the cued and uncued effects of *x* is given by *β*_3_ (for the tap probability model, this is the effect of *x* on discriminability). (See the Supplementary for a more in-depth explanation and an overview of the relationship to signal detection theory.)

All reported models fitted without divergences, had $\hat{R}$ values no higher than 1.02, and had at least 400 effective samples per effect. Posterior predictive checks confirmed that appropriate distributional families had been chosen.

##### Variable selection

For our rhythms, there are some extremely high multicollinearities between some of the predictors, which means there are considerable redundancies in the information they provide. Given that each predictor is individually meaningful, a PCA type approach—where the resulting subset of predictors lose their simple interpretation—is not desirable. In order to select a good subset of predictors, we use the technique of projective prediction variable selection as implemented in the R package projpred (Piironen et al., 2020; Piironen & Vehtari, 2017). This technique is related to the familiar forward stepwise selection but is protected from overfitting and double-use of the data by testing each model, with cross-validation, against a fitted reference model that contains all the predictors from which the subset is chosen. This procedure returns a sequence of predictors, ordered by decreasing predictive importance, and these can be successively added in order to improve the projected fit. We added predictors in this order, but any predictor that introduced a VIF greater than 10—for itself or any other predictor—was not added and we instead proceeded to the next predictor in the list. No previously added predictors were removed. This means that each final model contains a set of important predictors such that no predictor can be regressed on the remaining predictors with an *R*^2^ greater than 0.9. For reference, in the Supplementary we provide the correlations between the rhythm- and pulse-level predictors, across the three sets of data used in the models detailed below: the data differ because the tap accuracy model is at the rhythm-level, the tap probability model is at the pulse level, the tap velocity and timing models are also at the pulse level but only use data from pulses that are tapped).

## Results and discussions by model

In this section, we first provide and discuss data visualizations. We then report results from each of the models for rhythm-level accuracy, pulse-level tap probability, pulse-level tap velocity, pulse-level timing error. For these models, we focus discussion on effects that are strongly evidenced (posterior probabilities of direction greater than 0.975, which corresponds to an evidence ratio of 39).

### Rhythm- and pulse-level visualizations

**Fig. 2:**
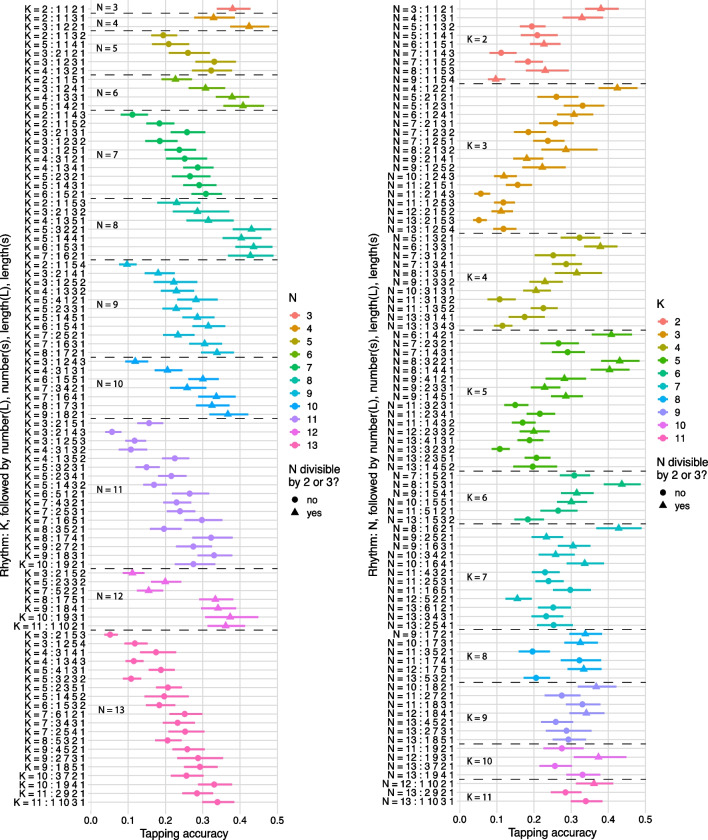
Tapping accuracy by rhythm (95% credibility intervals): on the left, ordered by 𝑁; on the right, ordered by 𝐾. The sequence of four numbers after each colon are the number of large IOIs, the number of small IOIs, the size (in pulses) of the large IOI, the size of the small IOI. (Colour figure online)

Figure 2 shows the rhythm-level tapping *accuracy* for all 91 rhythms. These are obtained from a model with intercept and rhythm randomly varying by participant; the R-style formula is: *tap* _ *acc *~ 0 + *rhythm* + (*rhythm* | *participant*). Hence, these are results for an average participant. On the left, the rhythms are ordered by *N* (number of pulses in the rhythmic cycle) and, within each *N*, by *K* (number of cues in each rhythmic cycle); on the right, they are ordered by *K* and, within each *K*, by *N*.

**Fig. 3:**
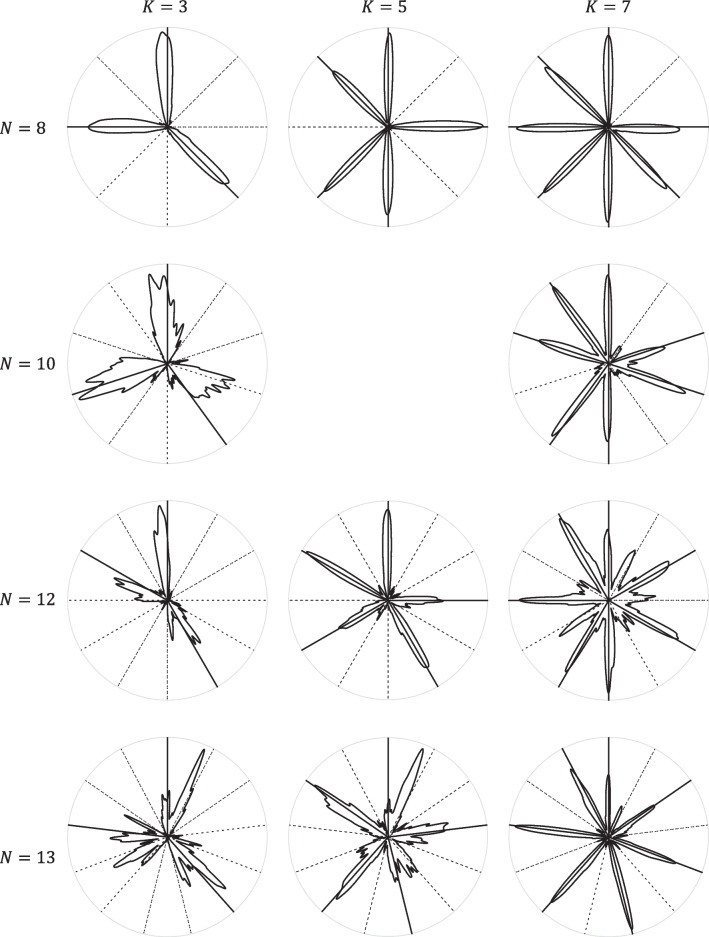
Velocity distributions smoothed across time (modulo the rhythm’s period) of a subset of 11 of the 91 rhythms performed. Solid radii mark cued pulses; dashed radii mark uncued pulses. The data are normalized for each rhythm

**Fig. 4:**
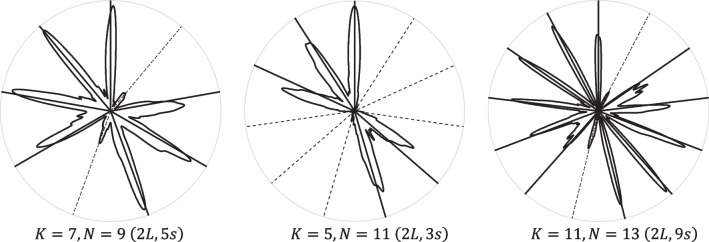
Velocity distributions smoothed across time (modulo the period) of three rhythms, all of which have two distinct clusters (groups) of cues. Solid radii mark cued pulses; dashed radii mark uncued pulses. The data are normalized for each rhythm

Figures 3 and 4 summarize tapping behaviour at the millisecond-level—they show the smoothed distribution of tap velocities over time (modulo the rhythm’s period), aggregated over all performances (and participants). The smoothing was achieved by circularly convolving the raw data with a Gaussian kernel with a standard deviation of 10 ms; the method is detailed in the Supplementary. The rhythms shown in Figs. 3 and 4 are useful examples because they demonstrate the wide range of tapping behaviours arising from combinations of three different *N* and three different *K*; analogous figures for every tested rhythm are available in the Supplementary.

#### Discussion of descriptive visualizations

There are some obvious patterns in the rhythm-level data, which are particularly noticeable in the left-hand plot of Fig. 3: rhythms with higher *N*, and lower *K* within each *N* (higher mean interonset interval), seem harder to tap. This is indicative of an interesting asymmetry: compare the two (*N*, *K*) = (13, 10) rhythms with the two (*N*, *K*) = (13, 3) rhythms. The former two are *complements* of the latter two—they are the “same” except the cued and uncued pulses are swapped. And yet the versions where the cues are more common are performed a lot more accurately, and this pattern seems quite consistent. There is possibly also an effect from whether the rhythm can be grouped into twos or threes.

Note that the shape of each of the “circle plots” in Figs. 3 and 4 arises from three possibly independent aspects of the data: the probability of a tap occurring at each periodic time location; the velocities of all occurring taps; the timings of all occurring taps. This means they give a good overview of the accuracy of the tapping and where consistent errors occur, both within and between rhythms. It is apparent from these examples (which are not exceptional: see the Supplementary for circle plots of all 91 rhythms) that some rhythms are tapped with considerably more accuracy than others and that, within each rhythm, some cues are tapped with greater probability, velocity, or temporal accuracy than are other cues. Overall tap accuracy is more precisely quantified by the model in the section Rhythm-level Tap Accuracy; the remaining three aspects are separated out in the sections Pulse-level Tap Probability, Pulse-level Tap Velocity, and Pulse-level Tap Timing Error.

Prior to reporting this more detailed modelling, it is useful to point out a few observations arising from these three figures:

1. Amongst the *N* depicted in Fig. 3, 8 is tapped most accurately and 13 least accurately. But one of the rhythms in 13, which has a large number of cues, is tapped, perhaps surprisingly, rather well.
2. Following on from the previous point, rhythms with only a few cues—the left-hand column in Fig. 3—seem to be harder to tap accurately than those with many closely spaced cues. This is consistent with the observation from Fig. 2
3. In rhythms with clear groups (clusters of cues), the first cue is often the weakest and the medial cues can be stronger than the last. This can be seen in Fig. 3, but is particularly clear in Fig. 4, which shows three rhythms with obvious two-group structures. The models in the sections Pulse-level Tap Probability and Pulse-level Tap Velocity help us to see whether this results from tap probabilities or from velocities of those taps.

The first of these observations is unsurprising: for our participants, 8 is a very commonly heard subdivision of the rhythmic cycle (e.g., eighth notes in common time), while 13 is very uncommon; furthermore, 10 and 12 (the former also being unfamiliar) can be metrically subdivided into a regular beat each comprising two pulses, while 13 cannot. The second observation was not expected beforehand; it is discussed in more detail later. The third observation is surprising because prior experimental evidence has shown that the starts and ends of clusters (groups) of cues are perceptually and performatively accented (Povel & Essens, 1985; Povel & Okkerman, 1981; Repp et al., 2005).

To determine more precisely which aspects of rhythmic structure are most relevant to which performance outcomes—overall accuracy, probability of tapping, velocity of taps, timing errors of taps—we now model each of these outcomes with the previously detailed predictors.

### Rhythm-level tap accuracy

#### Results

This Bayesian regression model is fitted over 4,975 observations of the accuracy of a complete performance (each of the 111 participants were asked to provide 45 or 46 performances; some performances were not provided resulting in a reduced total number). This number is sufficient to obtain strong evidence for the direction of small effects. From the full set of performance-level and rhythm-level predictors, the seven shown in Table 2 emerge after the variable selection process.

#### Discussion

The effect size for *mean* _ *IOI* is substantial. This confirms the previous observation that rhythms with more densely packed cues are tapped more accurately than rhythms with sparser cues.

As expected, *balance* has a negative impact—the more unbalanced a rhythm, the easier it is to tap. The balance of a rhythm is equivalent to its circular variance, so the lower a rhythm’s balance, the easier it should be to estimate its circular mean and, furthermore, the closer the cues are to that mean. Hence, for low-balance rhythms, tapping in the vicinity of the circular mean is easier and is more likely to result in correct taps. Somewhat related to this, the preponderance of cues in one half of the rhythmic cycle should generally facilitate position-finding within the rhythm.

Also as expected, *duple* _ *triple* has a positive effect—rhythms with a period (*N*) that is divisible by two or by three are easier to tap. It suggests that being able to metricize rhythms into duple or triple time, provides some cognitive or motoric advantage; as discussed earlier, it may reduce working memory demands by allowing a complete rhythm to be chunked from familiar subrhythms which start on a regular metrical grid. This coefficient may also result from the greater prevalence of, hence, participants’ familiarity with, duple and triple meters in music (of course, the prevalence of such rhythms may arise because they can be more easily metricized).

*Evenness* has a strongly evidenced weak effect; but in the opposite direction to that hypothesized (hypothesized, because uneven rhythms typically have one or more distinct clusters and gaps, and these should facilitate the identification of the grouping structure of the rhythm). This is likely because balance and evenness are so highly correlated (.94). The larger magnitude coefficient for balance, compared to evenness, shows that rhythms low in either balance or evenness are tapped more accurately; it is only when controlling for balance that there appears to be a small positive effect of evenness. But, given their high correlation, the precise values of these two coefficients will be variable across different data sets. Hence, the precise influence of balance relative to evenness and the effect of either conditioning on the other should be interpreted with some caution, and we can safely conclude only that rhythms with low balance or evenness are generally tapped more accurately.

Any effect of *IOI* _ *ent*, which was anticipated to have a negative effect on tapping accuracy, is likely miniscule (its 95% credibility interval is −0.05 to 0.05).

There is a small positive effect of *perf* _ *num*; as participants progress through the experiment, they perform (slightly) better—a clear indication they develop general strategies to improve their performance. However, any effect of *repetition* (the same rhythm being performed for the second or third time) is almost certainly miniscule.

### Pulse-level tap probability

#### Results

This Bayesian regression model is fitted over 48,282 binomial observations of the number of times each of the *N* pulses in each rhythms rhythmic cycle was tapped (111 participants providing 45 or 46 performances of rhythms, each comprising 3–13 pulses per cycle, on average about 10; some performances were not provided), which is sufficient to obtain strong evidence for the direction of small effects. As identified above, overall tapping accuracy is a function of tapping probability, velocity, and timing. In this subsection, we focus on the probability of tapping at each pulse. After the variable selection process, we obtain the following set of predictors.

The overall fit of the above “full” model—as assessed by the Bayesian *R*^2^ values—is very good. It is interesting to compare its fit with those of the three reduced variants shown in Table 4: a model with only cue as a predictor (randomly varying by participant and rhythm); a full model but with all random effects removed; a model with only cue and no random effects. It is apparent that a model with only cue still has an acceptable fit (e.g., its cross-validated *R*^2^ is 0.75) which reflects its large effect size (2.02); adding random effects moderately improves the fit; adding all the predictors provides an additional small improvement in fit, which is highly significant. (Note that, in the random effects models, the intercept and the effect of *cue* vary by rhythm and so can make up for any missing rhythm-level predictors; hence, the comparison between the “no random effects” models gives a better indication of the importance of the predictors beyond *cue*.)

**Table 4:** Measures of the fit of the full model reported in Table 3 compared with three reduced variants

	Bayes R2	CV R2	ΔELPD	SE
Full model (with random effects)	0.85	0.85	0.0	0.0
Only cue (with random effects)	0.83	0.83	−2910.1	75.1
Full model (no random effects)	0.79	0.79	−6979.9	118.3
Only cue (no random effects)	0.72	0.75	−11014.8	136.4

The columns show two types of *R*^2^ value (detailed in Table 2) and their cross-validated ELPD (expected log pointwise predictive density), which is an estimate of the model’s ability to predict out-of-sample data. Higher ELPD values are better, and differences between ELPDs can be considered significant when they are at least twice their standard error

**Fig. 5:**
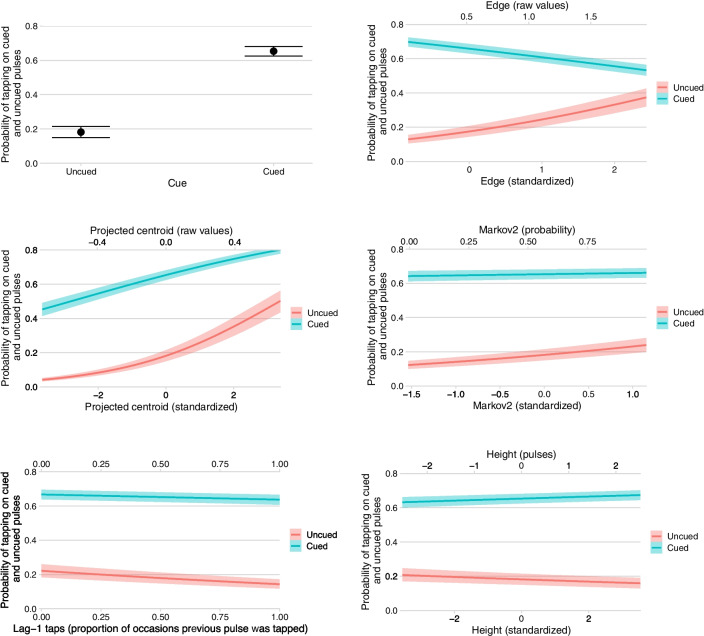
The effect of each pulse-level predictor on the probability of tapping on uncued (red) and cued (blue) pulses. Each effect is conditioned on all other predictors being at their mean values. The ribbons show 95% Bayesian credibility intervals. (Colour figure online)

The conditional effects plots in Fig. 5 show how each of the pulse-level predictors influences the probability of tapping incorrectly (when there is no cue) and correctly (when there is a cue) when all other predictors are at their mean. The more separated the two lines the greater participants’ discrimination between cued and uncued pulses. For example, we can see that as the *edge* predictor increases (i.e., for cues at the edges of distinct clusters), discriminability drops markedly; slightly differently, as *proj* _ *cent* increases (i.e., for cues close to an unbalanced rhythm’s centroid), the overall bias for tapping on-cue and off-cue strongly increases, while discriminability reduces a smaller amount.

#### Discussion

The meanings of the results for all the regression inputs are now verbally summarized and discussed, by level. Note that effects shown below are standardized (because all predictors were standardized prior to running the model); odds ratios (*OR*) are also included; unless specifically mentioned.

##### Group-level effects

Tapping behaviour differs substantially between participants and rhythms. For example, discriminability has a standard deviation of 0.78 between participants (a 1 standard deviation difference, therefore, corresponds to an odds ratio of e^0.78^ = 2.2), and 0.96 between rhythms (a corresponding *OR* of 2.6).

##### Performance-level predictors

*perf* _ *num* increases the discriminability of cues through increasing the number of cued taps, though the effect is relatively modest (0.17, *OR* = 1.18). The positive effect, on discriminability, of *repetition* is very small (0.07, *OR* = 1.08) and is achieved through reducing the number of uncued taps. In summary, participants slightly improve their general tapping strategies over the course of the experiment but show only marginal rote learning of individual rhythms between performances; although it is worth remembering that repeat performances of the rhythms were well separated because they occurred in different experimental blocks.

##### Rhythm-level predictors

*duple* _ *triple* has the expected large positive effect on discriminability (0.46, *OR* = 1.58)—cues are substantially better discriminated in rhythms that can be grouped into twos or threes without having to double the period of the rhythm (i.e., *N* = 3, 4, 6, 8, 9, or 12, rather than *N* = 5, 7, 11, or 13). The improvement in discriminability results mostly from participants tapping less on uncued pulses. This may be due to such rhythms facilitating chunking (as explained earlier in Predictors) as well as their greater familiarity (such rhythmic periods being substantially more common in many musical traditions, including Western).

The effect of 𝑚𝑒𝑎𝑛_𝐼𝑂𝐼 is interesting. As it decreases (i.e., cue density increases), the general probability of tapping (correctly or incorrectly) substantially increases (the effect on bias is −0.37, *OR* = 0.69) but there is no evidence for an effect on discriminability.

*CQ*—Carey’s coherence quotient—seems to play a role here (it did not survive the variable selection process for the previous rhythm-level model). But its effect is not as expected; higher-*CQ* rhythms result in more incorrect (uncued) taps (0.18, *OR* = 1.20) although there is not strong evidence that discriminability actually reduces.

Cues are more discriminable in longer (higher *N*) rhythms (0.18, *OR* = 1.20); note that we are controlling for *mean* _ *IOI*, so this is across different-length rhythms with similar cue density. This increase in discriminability results mostly from a lower probability of tapping incorrectly. It is not obvious what underlying mechanism would account for this effect of *N*.

The effect of *balance* is rather uncertain and there is no strong evidence of any specific directionality of the three effects of interest (note that all evidence ratios are below 39). This is probably because of the strong effect obtained from *proj* _ *cent*, discussed below, which is a pulse-level generalization of the rhythm-level *balance*.

##### Pulse-level predictors

The effect of *cue* (2.02, *OR* = 7.52) is very strong. Participants are nearly 8 times more likely to tap correctly on-cue than to tap incorrectly off-cue; this shows they were typically following the task we had set them and were learning the rhythms within each performance (remember the very small effect of *repetition*, which implies only a small amount of learning of specific rhythms occurred between performances). The strikingly high odds ratios may seem, at first sight, to contradict the relatively poor tapping accuracy values (*tap* _ *acc*) predicted in the previous model and illustrated in Fig. 2; but it is important to remember that the *tap* _ *acc* dependent variable penalizes timing errors, while the *tap* _ *num* dependent variable used in this model does not (so long as the tap falls into the 234 -ms window centred on the pulse). So, although discrimination between cued and uncued taps is rather good, the timings of those taps is not. The standard deviations of the group-level (random) effect of cue by rhythm (0.96) and of cue by participant (0.78) show that the rhythms differed substantially in the discriminability of their cues, and participants differed substantially in their ability to discriminate cues.

*Edge* represents a generalization of Povel’s accentual weights. Povel’s accents include pulses at the ends of groups of one or more cues, and at the starts of groups of three or more cues; *edge* also accents the starts and ends of groups of uncued pulses. The model shows that tap probabilities actually proceed in the opposite direction to the expected Povel accents: medial pulses (those not at the edges of clusters) are tapped more often, which confirms the observations made earlier in Rhythm- and Pulse-level Visualizations. For cued pulses, the effect of being at the edge of a cluster—as quantified by *edge*—decreases tapping (−0.22, *OR* = 0.81); for uncued pulses, the effect of being at the edge of a cluster of uncued pulses—as quantified by *edge*—strongly increases tapping (0.43, *OR* = 1.53). This indicates that participants are aware of cue-clusters but not sure when they will start: once they have heard the first cue in a cluster, they are confident the cluster has started and so they should now tap; given a long gap (a cluster of uncued pulses), they begin to anticipate the next cue-cluster is coming and so tap incorrectly before the cluster has started—the incorrectness of this tap perhaps then encouraging them to not tap in the next pulse, hence, missing a possible cue at the start of the cluster. In sum, participants are aware of the clusters but not sure exactly where they start or end.

These results for *edge* run counter to the theory and findings of Povel and Essens (1985); for this reason, and despite the above plausible explanation, we felt it advisable to check whether these results may arise from the precise way that *edge* generalizes Povel’s weights (which apply only to cues) to uncued pulses. To do this, we tested two additional models of tapping probabilities; both fitted only to cued pulses. One model contained the *edge* predictor, the other model replaced *edge* with three binary predictors indicating whether a cue was isolated (*iso* _ *accent*), at the end of a group of two or more cues (*end* _ *accent*), at the start of groups of three or more clusters (*start* _ *accent*)—these being the precise criteria used by Povel. Both models are fully summarized in the Supplementary but, in brief, both had an excellent fit to the data (cross-validated *R*^2^s of 0.80), and both confirmed, with very strong evidence, that cues at the edges of clusters were less likely to be tapped. The second new model showed that isolated cues were, as expected, tapped more. However, the extra level of detail provided by the three Povel indicators, compared to the single, hence, more parsimonious, *edge* predictor, is questionable because cross-validation shows the out-of-sample predictive performance of the two models cannot be reliably distinguished (although on balance of probabilities, the *edge* model is weakly favoured). This striking finding suggests that edge-finding plays an important role in guiding taps but, when the rhythms are particularly complex (e.g., many of ours have periods of 5, 7, 11, and 13), the precise locations of the edges are not correctly remembered or are displaced by duple or triple metrical expectations, which results in decreased discriminability at the boundaries between gaps and groups.

The effects for *tap* _ *lag*1 show that tapping on the previous pulse improves discriminability (0.40, *OR* = 1.49), principally by discouraging incorrect taps (−0.53, *OR* = 0.59) on the current pulse (correct taps are discouraged by a somewhat smaller amount). (Note that *tap* _ *lag*1 is not standardized like the other continuous variables; its standardized effects are given by multiplying its effects shown in Table 3 by the standard deviation of *tap* _ *lag*1 in the data, which is 0.348.) The overall reduction in tap probability may be an artefact of the rhythms performed because the proportion of consecutive cues is 0.333 (and taps occur more commonly on cued pulses than on uncued pulses); it may also result from the short-term fatigue induced by making a tap, which discourages a subsequent tap (regardless of its correctness). The increase in discriminability may be due to a prior tap acting as a confirmation that the participant is at least attempting to perform the rhythm rather than “sitting it out”.

*Proj* _ *cent* encourages participants to tap on cue (0.23, *OR* = 1.25) but encourages them even more to tap off-cue (0.45, *OR* = 1.56); hence, reduces discriminability (−0.22, *OR* = 0.80). It seems that although the information provided by the rhythm’s centroid—tap close to strong rhythmic centroids; do not tap close to the opposite of strong rhythmic centroids—is very influential for behaviour, it is actually a rather poor tapping strategy for discriminability. As with *edge*, this perhaps suggests that *proj* _ *cent* may be a useful mechanism in more conventional rhythms—such as those with unsyncopated groups—but that it fails for the mostly multi-onset uneven rhythms used in this study.

*Markov*2 strongly encourages participants to tap off-cue (0.30, *OR* = 1.35) and so reduces discriminability (−0.27, *OR* = 0.76). This suggests that they were relying, in part, on a Markovian representation, or some similar lossy representation of the pattern in terms of how very short-term rhythmic figures tend to continue within that pattern. Interestingly, this effect seems to operate in one direction only: we did not find a tendency *not* to tap on cued pulses with *low Markov*2. A hybrid mechanism for remembering patterns that combines rote memory of a few memorable features with lossy mechanisms for the pattern’s remaining features suggests a possible explanation for this asymmetric effect. It seems likely that a sound (i.e., a cue) that defies a subrhythmic regularity is more memorable than a “silence” that defies a subrhythmic regularity (just as onsets make a stronger impression than offsets). Using such a hybrid mechanism, a participant might—with relative ease—learn the surprising cues by rote, but perform more poorly at the surprising silences, misled by a lossy mechanism into tapping incorrectly. This is reflected in our earlier observation, related to Fig. 2, that there is a tap-accuracy asymmetry between rhythms and their complements—the version with higher *K* is typically tapped more accuractely.

The effect of *mean* _ *offset* is very small—it marginally decreases off-cue taps (−0.05, *OR* = 0.95) and marginally increases on-cue taps (0.03, *OR* = 1.03); hence, marginally increases discriminability (0.07, *OR* = 1.07). Generally, for well-formed rhythms, pulses with high *mean* _ *offset* are close to the ends of grouped cues (sequences of small IOIs); pulses with low *mean* _ *offset* are close to the starts of grouped cues. This effect, therefore, suggests a predictive mechanism that pays particular attention to sequences of short-IOI cues—perhaps the number of cues in each cluster is learned and once the participant has recognized that that cluster has started, they can enact that knowledge. However, this effect is very small.

### Pulse-level tap velocity

#### Results

This Bayesian regression model is fitted over 303,903 observations of tap velocity (111 participants made 45 or 46 performances, with an average of about 60 taps per performance; i.e., a tap approximately every 2 pulses; some performances were not provided), which is sufficient to obtain strong evidence for the direction of small effects. The model for tap velocity was fitted only to pulse-level observations where a tap had occurred. For the tapped pulses’ velocities, a Gaussian family model was used and values at the maximum possible MIDI velocity level (127) were treated as censored (i.e., they were, correctly, modelled as meaning ≥127). Following the variable selection process, the model summarized in Table 5 emerged.

**Table 5:**
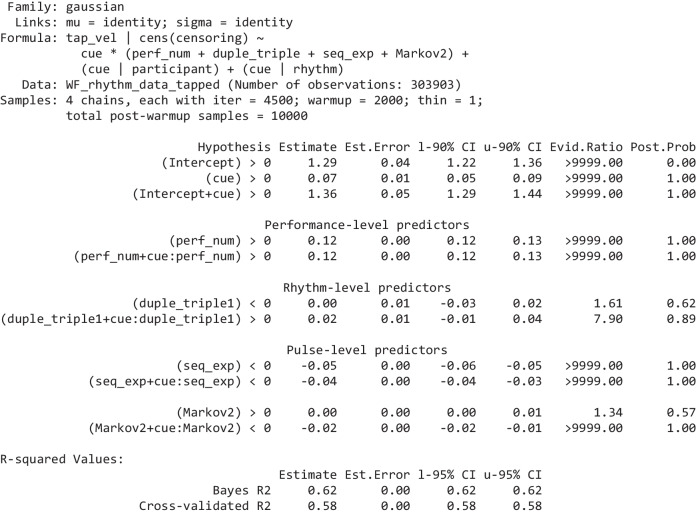
Summary of the model of pulse-level tap velocity

The two-line groupings, for every predictor except cue, show Bayesian hypothesis tests for two effects: In the first line, the predictor’s effect on the velocity of incorrect taps; in the second line, its effect on the velocity of correct taps. The effects are grouped by level (performance, rhythm, pulse). The columns are the same as Table 2, as are the methods for calculating the *R*-squared values. Full model summaries, including the group-level effects are provided in the Supplementary

#### Discussion

Every effect is small to vanishing. The largest is *perf* _ *num*, which has a decisively evidenced but small effect of 0.12—with each successive performance, participants tend to tap slightly harder. The remaining predictors’ effects—even *cue*—are vanishingly small. The model has impressive *R*^2^ values but these are due mostly to the differences between participants as captured by the participant-level variation in the intercept, which has a standard deviation of 21.7 MIDI velocity units—some participants generally tap substantially harder than others. Variation between rhythms was trivial. (An otherwise equivalent model without group-level effects has Bayesian and cross-validated *R*^2^ values of only 0.05).

It is interesting to note that *edge* does not make it through the variable selection process, so has weak predictive power for tap velocity. Hence, we have no evidence that the starts and ends of clusters are tapped harder than are medial cues. Once again, this does not align with prior hypotheses in Povel and Essens (1985) nor the tap velocities observed in Repp et al. (2005), where starts and ends of clusters were tapped harder than medial onsets. As before, this suggests that Povel and Essen’s accents at the edges of groups are only manifested in simpler rhythms or rhythms that have been fully learned such that the precise locations of these edges is actually remembered.

### Pulse-level tap timing error

#### Results

This Bayesian regression model is fitted over 303,903 observations of tap timing error (111 participants made 45 or 46 performances, with an average of about 60 taps per performance; i.e., a tap approximately every 2 pulses; some performances were not provided), which is sufficient to obtain strong evidence for the direction of small effects. For the signed tap-timing error, a truncated Gaussian family model was used; due to the assignment of each tap to its closest possible pulse and the deletion of any duplicate taps within a single pulse window, which is in the interval [−117, 117] ms relative to each pulse, the data are truncated outside that interval and are modelled accordingly. Following the variable selection process, the model summarized in Table 6 emerged.

**Table 6:**
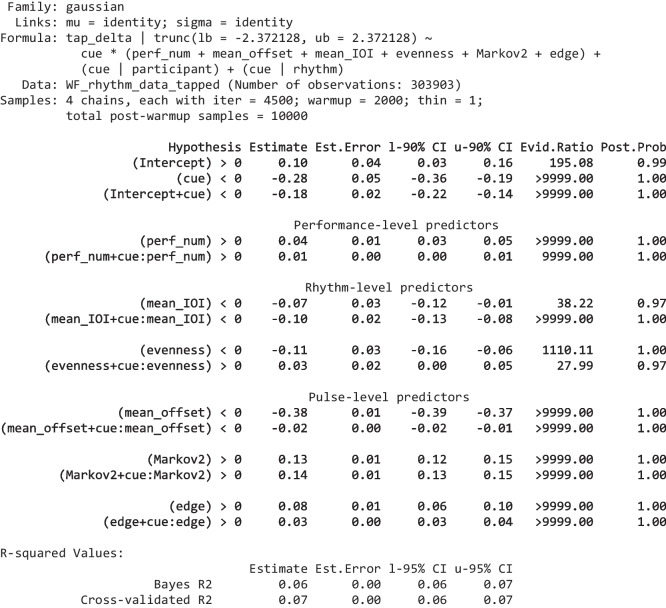
Summary of the model of pulse-level tap timing error

The two-line groupings, for every predictor except cue, show Bayesian hypothesis tests for two effects: In the first line, the predictor’s effect on the timing of incorrect taps; in the second line, its effect on the timing of correct taps. The effects are grouped by level (performance, rhythm, pulse). The columns are the same as Table 2. Full model summaries, including the group-level effects are provided in the Supplementary

The model’s overall fit to the data is poor, indicating that timing behaviour is not particularly influenced by between-participant or between-rhythm effects (the group-level standard deviations of the intercept and cue are, respectively, 9.4  ms and 14.3  ms by participant; 14.8  ms and 19.2  ms by rhythm). We do, however, obtain some predictors with nontrivial effects.

#### Discussion

Cued (correct) taps are earlier than uncued (incorrect taps). With all other predictors at their means or reference levels—a cued tap is predicted to occur about 9 ms before the pulse; an uncued tap about 4.8 ms after the pulse; a small but meaningful difference of 13.8 ms. For simple isochronous rhythms, performers typically exhibit a negative mean asynchrony (for nonmusicians, approximately 20–80  ms before the auditory cue; Aschersleben, 2002), and this is indicative of the performers making anticipated rather than reactive taps (bearing in mind, of course, that a tap would have to be late by at least 150 ms to be reactive; Repp, 2003). Given that cued pulses are more likely to be anticipated than uncued (the tap probability model shows that cued pulses are tapped with substantially greater probability than uncued pulses), this suggests that earlier tap timings may be a useful proxy for the extent to which the tap is based on a strong prediction (regardless of whether that prediction is correct).

Uncued pulses with higher *mean* _ *offset* are tapped moderately earlier (a unit increase in *mean* _ *offset* has an effect of −0.38, which corresponds to −18.8 ms). For well-formed patterns, uncued high *mean* _ *offset* pulses come soon after a group of cues (a sequence of small IOIs); uncued low *mean* _ *offset* pulses come shortly before a group of cues (in both cases, the precise value of an uncued pulse’s *mean* _ *offset* depends on the number of cues in the preceding or proceeding cue cluster and how far it is from that cluster, although other factors also play a role). To the extent that tap timing is reflective of how strongly the participant has predicted that tap, these results suggest that participants more strongly anticipate a cue following a sequence of fast cues (i.e., a cue cluster). Typically, we would expect predicted taps to occur early—the negative mean asynchrony effect—but note that for incorrect taps (all else at the mean) the intercept predicts taps will be almost 5-ms late; the effect of *mean* _ *offset*, which is substantial only for uncued taps, has the potential to turn this into an early tap for pulses where a tap is predicted, but predicted incorrectly. The tap probability model shows that these incorrect predictions of high *mean* _ *offset* pulses are uncommon but, when they do occur, they occur early. The lack of an effect of *mean* _ *offset* for cued pulses does not obviously replicate those of Repp et al. (2005) and Snyder et al. (2006) who found that, within groups, taps (only correct taps were assessed) occur progressively earlier.

Taps (correct and incorrect) are slightly later for pulses with greater *Markov*2 (an effect of 0.14 corresponds 6.7 ms). There is no obvious mechanism to account for why taps motivated by a Markov-like process would occur later, but the timing differences are very small.

The effects of *evenness* on incorrect taps, and of *mean* _ *IOI* on correct taps, are small (e.g., a unit increase in either induces taps about 5 -ms earlier). There is no obvious mechanism to explain these small shifts: if either rhythmic feature were to encourage participants' confidence in their predictions—which might induce negative mean asynchrony—it is not clear why this would not apply similarly to both correct and incorrect taps. All other effects are vanishingly small and so not discussed further.

## General discussion

In summary, the most important pulse-level predictor of tap probability, velocity, and timing is (unsurprisingly) *cue*: cued pulses are substantially more likely tapped than are uncued pulses, although its effect on velocity and timing are only small. This shows that participants were following the task and were overall able to discriminate between cued and uncued pulses. At the same time, the rhythm-level accuracies and the circle plots (Figs. 3 and 4 and the Supplementary) show that performances had many incorrect and badly timed taps, which highlights the challenging nature of most of the rhythms.

At the performance level, the overall number of performances is more important than the number of times a given rhythm had been performed: Tap discriminability increased, as did tap velocity, over the duration of the experiment. This suggests “meaningful learning” where participants developed or refined strategies over the course of the experiment. Rote learning, on the other hand, had only a small or no effect, which is unsurprising given that each rhythm was usually played only twice and usually with other rhythms occurring in between.

Although the remaining rhythm- and pulse-level predictors play only weak roles in velocity and timing, they have substantial effects on tap probabilities. As such, they provide useful insights into the nature of the above-mentioned strategies and how effective they were, as now discussed.

To tap precisely to a rhythm, a performer must, somehow, store, retrieve, and process a lossless encoding of its cues’ onset times. The difficulty of doing this likely depends on the quantity and rate of information content in the rhythm, and conformance with prior expectations, which are influenced by the following:

1. The overall information content of any rhythm increases with its number of cues (*K*).
2. The rate at which that rhythm-level information is imparted is a function of the length of the rhythmic cycle (*N*).
3. The rate at which this information is imparted is given by the mean speed of the cues (*mean* _ *IOI*).
4. Prior familiarity with that rhythm or aspects of its structure (*duple* _ *triple*).

There are strong multicollinearities (mutual redundancies) between *K*, *N* and *mean* _ *IOI*. Our variable selection process favoured *N* and *mean* _ *IOI* over *K*: the effect of the first is in line with *point* 2, but the effect of *mean* _ *IOI* runs counter to *point* 3; we discuss a reason for this below. We see a strong effect of *duple* _ *triple* on discriminability (*point* 4). This may be due, in part (see below for a complementary “bottom-up” explanation for *duple* _ *triple*), to our participants’ greater familiarity with duple and triple time (particularly the former) who will likely have had their expectations “thrown” by periods that last one pulse more or one pulse less than commonly heard. A more sophisticated analysis of familiarity, given a representative corpus, would be obtainable with the IDyOM model (Pearce, 2018); our focus is on intrinsic structural features of rhythms.

Different mental representations of the rhythm across time should also impact on cognitive demands. In particular, metricization should reduce demands on short-term memory by breaking a rhythm into nonminimal chunks, each of which starts at an easy-to-remember isochronous beat, and can take only a limited number of forms; for example, if chunks are of two pulses, they can take only four forms: (0 0), (0 1), (1 0), or (1 1). Metricization like this is more likely if:

5. the rhythm allows metricization over chunks comprising a sufficiently small amount of information (e.g., of no more than three pulses, *duple* _ *triple*);
6. the cues are close enough in time to induce a strong beat (*mean* _ *IOI*) (e.g., beats of around 500 ms are easier to induce than those that are substantially longer or shorter).

Our results for *duple* _ *triple* and *mean* _ *IOI* support the role of metrical chunking into groups of 2 or 3 pulses in rhythms with smaller interonset intervals (*points* 5 and 6), although we cannot be certain to what extent *duple* _ *triple* is indicative of familiarity or metricization.

Given that the distribution of possible rhythms heard by a person over their lifetime is nonuniform (i.e., it has lower than maximal entropy), there will be lossless codings that compress common rhythms at the expense of uncommon rhythms (it is a consequence of Shannon’s source coding theorem that a lossless coding that reduces the size of common items must increase the size of rare items). Any such lossless coding, like metrical chunking, may, therefore, reduce the cognitive load of common rhythms whilst increasing the load of unusual rhythms (as indicated by the positive effect of *duple* _ *triple*, because grouping into twos or threes is likely to disrupt performances in the rhythms with cycles of 5, 7, 11, or 13 pulses). For rhythms that are too complex to be losslessly compressed sufficiently to avoid overwhelming cognitive resources, or rhythms whose rarity means they are not helped by established lossless encodings, lossy encodings may play a role. Such encodings do not perfectly reproduce the rhythm—indeed, they may be crude and simplistic—but they have the capacity to increase the probability of correctly discriminating between cued and uncued pulses. Simple and lossy summaries (some statistical in nature) that might improve discriminability, and which can be indicated by our predictors, include:

7. For a rhythm that is similar to an isochronous beat (*APM* and *seq* _ *exp*), it makes sense to focus on that isochronous beat as a crude summary of the rhythm: it is easy to remember and tap along with, and doing so will result in good discriminability.
8. Cue density (*mean* _ *IOI*) might modify the criterion at which a tap is made—if cues are densely packed, the criterion for tapping reduces (the strategy, conscious or not, is “cues are occurring often, so it’s a good idea to tap often even if I am not completely sure I should”, and its converse “cues are rather sparse, so I will refrain from tapping unless I am very sure I should”).
9. Approximate positions of groups and gaps (clusters of cued and uncued pulses) can facilitate discrimination (*edge*, *mean* _ *offset*)—even if a performer is not sure exactly when each cue occurs, if a group is heard to have started, the next pulse is probably a good time to tap (this mechanism is more effective in more uneven rhythms because these have more obvious groups, *evenness*).
10. The circular mean and variance of the rhythm would allow for a rough guess of when a cue is more likely to occur (*proj* _ *cent*) (and, clearly, this strategy would work better the more unbalanced the rhythm, *balance*).
11. Asymmetries in the rhythm (low *balance* or *evenness*) help the performer to position-find.
12. Small common patterns within each rhythm—for example, *n*-grams of consecutive pulses (*Markov*2, 3, 4, …)—may guide tapping decisions; even if imperfectly.
13. In rhythms with lower interonset interval entropy (*IOI* _ *ent*, *int* _ *ent*, *SQ*), knowledge of the distribution (or number) of IOIs would allow for better-informed guessing of when to tap next (i.e., choose the commonest IOI).
14. For high *CQ* rhythms, cognizance of generic intervals informs guesses of their IOIs.

The absence of any notable effect of *APM* (which did not make it through the variable selection process in any of the models) or of *seq* _ *exp* suggests that our participants did not make direct use of metricization (*point* 7); they did not prefer tapping, or tap harder, at cues occurring in time with isochronous beats that might have been plausibly induced by the rhythmic cues. This suggests that the strategy indicated by *duple* _ *triple* is, for these rhythms and performers, more related to participant familiarity with the rhythmic period (*point* 4) rather than to the bottom-up metricization (*point* 5). Our models show a strong negative influence of *mean* _ *IOI* on the probability of tapping (cued or uncued), hence, supports *point* 8.

The remarkably strong effect of the *edge* predictor on discriminability supports *point* 9 (also clear in the descriptive plots, Figs. 3 and 4). It seems that our performers are aware there are groups of cues but unsure of their precise start time or their length; so they frequently tap, incorrectly, before the group has started or wait for confirmation that the group has started and then start tapping with gusto (but starting later than they should have) until they suspect the group is about to end or, indeed, hear that it has ended (hence, they tap one more time than cued). Hence, discriminability around the boundaries of groups is strongly reduced. This crude predictive mechanism works well for pulses in the centres of groups, which are often larger in number than the poorly discriminated pulses at the edges of groups; so, this is an overall reasonable approach for the participants to take. These findings, as well as the absence of any effect of *edge* on velocity, seem to contradict Povel’s model for metrical accenting being stronger at the starts and ends of groups (Povel & Essens, 1985) and the results of Repp et al. (2005), but they are unsurprising if the groups’ start times and lengths are perceptually important features but their positions are remembered only to a low order of approximation. In this way, our results can be consistent with those using simpler or more familiar rhythms where edges are tapped with greater certainty: in both scenarios, edges are important guides for tapping behaviour.

*Points* 10 and 11 are supported by the finding that less balanced rhythms are tapped moderately more accurately and with better discrimination; and pulses close to the circular mean are tapped substantially more often. The former result parallels findings that unbalanced rhythms are more commonly classed as having been heard before (regardless of whether or not they have) and are generally preferred (Milne & Herff, 2020). The results here suggest that participants can approximately estimate the rhythm’s circular mean and use that knowledge to guide their tapping: “the mean is about here so tap about now!” (We do not mean these quotes to imply this must be a conscious strategy.) In terms of discriminability, however, this is not a wholly successful approach: Close to the mean, incorrect taps increase more than correct taps (conversely, of course, tapping has more discrimination distant from the mean). The fact that imbalance has a moderately positive effect perhaps shows that, despite this intemperate tapping behaviour, a rhythm with more clearly defined “halves” (one more “full”, one more “empty”) may facilitate position-finding (knowing where one is) within the rhythm (*point* 11).

Small distinctive features in the rhythms—common trigrams of pulses, cued or uncued (*Markov*2)—are remembered and guide tapping decisions (*point* 12); even though this often results in uncued pulses being incorrectly tapped.

Neither type of IOI entropy nor *SQ* (*point* 13) obviously influence tapping. A surprising finding is that the coherence quotient (*CQ*) has a negative rather than positive effect on discriminability, mostly though increasing incorrect taps (hence, *point* 14 is not supported either). Perhaps, for our 91 rhythms, the coherence quotient is correlated with some unidentified feature that strongly affects discrimination, but it is not clear what that would be.

In sum, our results point towards several underlying strategies that guide taps to complex and mostly unfamiliar rhythms such as these. At the rhythm level, the most significant of these are related to cue density (*mean* _ *IOI*), which can cause performers to over compensate for dense or sparse rhythms; familiarity with duple or triple rhythmic structures (*duple* _ *triple*), which results in expectations going awry in rhythms with 5, 7, 11, or 13 pulses per cycle; memory decay, which causes longer rhythms to be tapped less accurately; and the utility of position-finding, which can be aided by more asymmetrical (less balanced or even) rhythms. At the pulse-level, the most significant of these are identifying perceptually prominent edges between groups and gaps; estimating the centroid of the rhythm, which is a heuristic for finding reasonable time ranges to tap; and the detection of short repeating patterns within each rhythm.

The key results of our earlier time series analyses (Dean et al., 2021) are supported and substantially extended here. In the time series models, each performance was studied intact, and some models treated the whole performance set in a single model. This allows clarification of autocorrelation and effects of learning and of some specific rhythmic features. But it does not treat individual taps or rhythmic cycles separately, and, hence, lacks the multiplicity of observations and attendant statistical power that is exploited here. Our other preceding paper (Bulger et al., 2022) uses point process models to demonstrate on a minute time scale (resolution of a few msecs) a quite different complementary aspect of the rhythmic performances: the participants’ changing propensity to tap, and the distinction between the different rhythm types in that respect. It again uses performance-level and cycle-level data rather than isolating individual tap data.

Cultural exposure to rhythms influences rhythmic tapping behaviour and which rhythms can be tapped accurately (Cameron et al., 2015; Jacoby & McDermott, 2017; Polak et al., 2018). Given the music-cultural homogeneity of our participants, we cannot determine whether the mechanisms we identify above are moderated by cultural exposure. Most of our rhythms would have been unfamiliar to our participants—particularly those where *N* is factorized by a prime higher than 3—and it is plausible that a person familiar with such *N* (e.g., a Balkan familiar with *aksak*) may have performed them more accurately than most of our participants. Furthermore, all but six of our participants had less than five years of regular musical practice. It is likely that trained musicians—particularly those with experience of unusual rhythms—would approach the task differently; for example, explicitly counting out the rhythms or making use of lossless encodings that do not so strongly penalize these complex rhythms. Having said that, a personal observation from two of this paper’s authors, both of whom have experience playing complex rhythms, is that these rhythms are hard to replicate purely from hearing them, but they become substantially easier to perform if notation is provided.

### Conclusion

For these mostly difficult and unusual rhythms, participants' taps are disrupted by unfamiliar cyclic periods (as quantified by *duple* _ *triple*) and, to a large extent, guided by crude representations of the rhythm: notably, its density (as quantified by *mean* _ *IOI*), its circular mean and variance (as quantified by *balance* and *proj* _ *cent*), the approximate positions of groups of cues (as quantified by *edge*), and low-order Markov approximations (as quantified by *Markov*2). Furthermore, these cognitive mechanisms are often counterproductive for discriminating between cued and uncued pulses, which was the task participants were asked to undertake. It also seems that these are quite different to mechanisms—such as metricization and emphasizing group boundaries—thought to guide tapping behaviours in learned and familiar rhythms; this is further underlined by the negative mean asynchrony of taps being much smaller than is typical for simple rhythms.

These findings increase our understanding of the types of lossy encodings used to internally represent unfamiliar and complex periodic temporal patterns. From a musical perspective, they are particularly relevant to understanding how listeners perceive the complex (and possibly unfamiliar) rhythms found in many non-Western musical traditions and within more experimental Western genres.

### Supplementary Information

ESM 1 (PDF 4.39 MB)
