# Supplementary material for: The effects of rhythmic structure on tapping accuracy
Source: Atten Percept Psychophys. 2023 Oct 10;85(8):2673–99. doi: 10.3758/s13414-023-02778-2 (PMC10600317; doi:10.3758/s13414-023-02778-2)
Supplement: Supplementary file 1 — (PDF 4.39 MB) [file 13414_2023_2778_MOESM1_ESM.pdf]

Supplementary for “The effects of rhythmic structure on tapping accuracy”

Andrew J. Milne[a,*], Roger T. Dean[a], David Bulger[b]

[a] *The MARCS Institute for Brain, Behaviour and Development, Western Sydney University, Penrith, Australia*
[b] *Department of Mathematics and Statistics, Macquarie University, Sydney, Australia*

S. 1 Performance-level tap accuracy method and calculation

We measured our performers' tap times and velocities, and the stimulus times. For each 30-second performance, it is useful to find a way to summarize these data to produce a single number that serves as an effective estimate of the *tapping accuracy* of each 30-second performance. For our purposes, a measure of tapping accuracy ideally fulfils the following criteria:

- Penalizes non-performance. For example, if a performer is silent for the first half of the performance, but then taps perfectly for the second half, it seems reasonable that their tapping accuracy should be half that of a performer who taps perfectly throughout the entire stimulus.
- Monotonically and symmetrically penalizes timing errors. The greater the difference between the tap time and the stimulus time, the lower the accuracy. For example, if a performer taps perfectly except for one cue where they tap 50 ms early this should result in the same accuracy as if they tap 50 ms late; furthermore, this accuracy should be lower than that achieved by a performer who taps perfectly except for one cue where the tap is 10 ms early.

A way to neatly meet these criteria is to represent both the stimulus and the taps in indicator vectors $\boldsymbol{x}$ and $\boldsymbol{y}$ with high temporal resolution; that is, $x_t = 1$ when there is a cue at time t, otherwise $x_t = 0$; $y_t = 1$ when there is a tap at time t, otherwise $y_t = 0$ (hence the velocity of taps plays no role in this measure, although it could if it were considered useful). We use a temporal granularity of 1 ms; that is, each successive t is 1 ms after the previous. The two vectors $\boldsymbol{x}$ and $\boldsymbol{y}$ are each circularly convolved by a truncated normal distribution (a *kernel*). The resulting distributions are mathematically equivalent to adding normally distributed noise to the tap times and the cue times, and serve to approximate inaccuracies of continuous time perception. Indeed, the standard deviation σ of the kernel models the probability distribution of possible timings that would be perceived under a time difference limen (just noticeable time difference) of σ milliseconds. For these data, we set the standard deviation to 10 ms, which worked well for the visualizations above and which is similar to the 6 ms just noticeable difference for isochronous sequences (Friberg & Sundberg, 1995). The similarity of these two vectors is then computed using their cosine similarity: $\frac{\langle \boldsymbol{x},\boldsymbol{y}\rangle}{\sqrt{\langle \boldsymbol{x},\boldsymbol{x}\rangle\langle \boldsymbol{y},\boldsymbol{y}\rangle}}$, where $\langle \boldsymbol{x}, \boldsymbol{y}\rangle$ denotes the inner product of $\boldsymbol{x}$ and $\boldsymbol{y}$. This dependent variable is denoted tap_acc, and is a real number in the unit interval.

S. 2 Mathematical specification of predictors

Here, mathematical specifications are provided for the more complex predictors outlined in the main text. In the equations below, all indices of the predictors and the rhythm's indicator vectors, are denoted by n taken modulo N, which is the number of pulses in the rhythmic period. Cues are indexed by k and when necessary also by j, both taken modulo K, which is the number of cues in the rhythmic period. We represent a rhythm's indicator function by $\boldsymbol{x}$, which is a binary vector with N entries indexed by periodic pulse number $n \in \{0, 1, \ldots, N - 1\}$ where 1 represents a cued pulse and 0 represents an uncued pulse.

- $balance$ and $evenness$: Let us represent a rhythm $\boldsymbol{x}$ with a vector $\boldsymbol{t}(\boldsymbol{x})$ of the times its K cues occur in ascending order and in pulse units; for instance, the rhythm with indicator vector $\boldsymbol{x} = (1\ 0\ 1\ 0\ 1)$ has $\boldsymbol{t}(\boldsymbol{x}) = (0\ 2\ 4)$. Balance and evenness are given by

$$balance(\boldsymbol{x}) = 1 - \left|\sum_{k=0}^{K-1} \mathrm{e}^{2\pi\mathrm{i}(\boldsymbol{t}(\boldsymbol{x})_k)/N}\right| / K$$

$$evenness(\boldsymbol{x}) = \left|\sum_{k=0}^{K-1} \mathrm{e}^{2\pi\mathrm{i}((\boldsymbol{t}(\boldsymbol{x})_k/N)-(k/K))}\right| / K$$

- IOI_ent: Entropy quantifies the unpredictability of a probability mass function. *Normalized entropy* is a unit-less form of entropy suitable for comparisons across probability mass functions with differing supports. The normalized entropy of probability mass function $\boldsymbol{x}$ is given by $H(\boldsymbol{x}) = -\frac{1}{\log N}\sum_{n=1}^{N} x_n \log x_n$, where $\log N$ is the maximum possible entropy for a probability mass function with a support of size N. If the mass function is dominated by a small number of different outcomes with relatively high probabilities, new outcomes are easier to predict correctly and the entropy is low; if the mass function has a large number of different outcomes with similar probabilities, new outcomes are harder to predict correctly and the entropy is high. To calculate the interonset interval entropy, we calculate the probability mass function of all interonset intervals (between consecutive cues) in one period of the rhythm. For example, in the rhythm $(1\ 0\ 1\ 0\ 1)$, there is one IOI with a 1-pulse duration, and two IOIs with durations of 2 pulses; there are no IOIs of any other duration. Hence, the probability mass function over the two different IOIs is $x = (1/3, 2/3)$. The entropy of this is calculated and then normalized by dividing by $\log 5$ (because there are five possible IOIs in a rhythm with 5 pulses to the period).
- int_ent: Interval entropy is calculated in exactly the same way as IOI_ent except that IOIs between all pairs of cues in the rhythm – not just consecutive cues – are considered.
- CQ: The coherence quotient is one minus the number of coherence failures divided by the maximum number of coherence failures possible for a rhythm with K cues (Carey, 2007). A *coherence failure* occurs whenever $x_{j+\ell} - x_j > x_{k+m} - x_k$ (subscripts taken modulo K) and $\ell < m$, In words, a coherence failure occurs when a pair of cues with a larger generic size (the generic sizes are ℓ and m) than another two cues does not have a greater specific size (the specific sizes are $x_{j+\ell} - x_j$ and $x_{k+m} - x_k$). We would expect rhythms with higher CQ to be easier to tap along with. For rhythms with two cues, the maximum possible number of coherence failures is zero, hence the coherence quotient is undefined. We replace these values with a coherence quotient of 1; this is somewhat arbitrary but there are only 9 such rhythms in our set of 91.

- SQ: The sameness quotient is one minus the number of different specific sizes taken by all generic intervals $\sum_{m=1}^{K-1}\left|\{x_{k+m} - x_m : k \in \{0, 1, \dots, K-1\}\}\right|$ divided by the maximum of such sizes possible for a pattern with K cues. We would expect rhythms with higher SQ to be easier to tap along with.
- seq_exp: *Sequential expectation* simulates the expectation that pulse x_n is a cue, conditioned on specific timing patterns of prior cues: if there has been a sequence of regular (evenly spaced) cues, the predictor assigns a higher expectation of a cue occurring on the pulse that continues that sequence. However, the influence of each cue is weighted by its lag measured by numbers of cues (denoted ℓ), and the size of its interval onset intervals (denoted m). These weights are the respective nonlinear parameters λ and μ, which are both positive real numbers. Expected parameter values would be greater than 0, because this implies that cues further back in the sequence are less important, and cues belonging to sequences with longer IOIs are less important.

$$seq\_exp(\boldsymbol{x}; \lambda, \mu)_n = \sum_{\ell=1}^{L}\sum_{m=1}^{M} \ell^{-\lambda} m^{-\mu} x_{n-\ell m} \,/ \sum_{\ell=1}^{L}\sum_{m=1}^{M} \ell^{-\lambda} m^{-\mu}$$

For computational feasibility, the upper bounds of the summations are set to values L and M sufficiently large that $L^{-\lambda}M^{-\mu} \approx 0$. In our calculations, we started with $L = 27$ and $M = 13$, which would be sufficient for $\lambda, \mu > 1$ and could be revised upwards if either parameter was estimated close to, or below, 1 (which they were not). The nonlinear parameters λ and μ must be optimized with cue also included in the model, or they will become artificially inflated to boost the Nth lag to make up for the missing (and likely important) zero-lag represented by cue (note that lags $0, N, 2N, 3N, \dots$ are equivalent because all indices are taken modulo N). The parameters were optimized to the tapping data separately from the other predictors; the resulting values are $\lambda = 2.2367$ and $\mu = 2.1397$; hence, the importance of cues in sequences is heavily penalized by their sequence position and the IOI sizes of the sequence.
- APM: The column sum of the autocorrelation phase matrix is calculated by

$$APM(\boldsymbol{x})_n = \frac{1}{N}\sum_{\ell=0}^{N-1}\sum_{i=0}^{N-1} x_{\ell i+n}\, x_{\ell(i+1)+n}$$

- $mean\_offset$: The normalized mean temporal offset between the rhythm's cues and the pulse of interest (inspired by the descriptions of pitch height given in Temperley & Tan, 2013 but generalized to uncued pulses) is given by

$$mean\_offset(\boldsymbol{x})_n = \frac{1}{N}\sum_{m=1-N}^{N-1} m\, x_n x_{n+m}$$

In this way, the calculation subtracts all periodic cue intervals, from x_n, that occur before (below) x_n from all periodic cue intervals, from x_n, that occur after (above) x_n.

- $proj\_cent$: When all pulses are represented as complex numbers on the unit circle, the *projected centroid* is the signed projection of the centroid onto the diameter passing through the pulse in question

$$proj\_cent(\boldsymbol{x})_n = |r| \cos\left(\arg(r) - \frac{2\pi n}{N}\right), \text{where}$$
$$r = \frac{1}{K}\sum_{n=0}^{N-1} x_n \,\mathrm{e}^{-\frac{2\pi i n}{N}}$$

- $edge$: Due to circularity, we use a discrete kernel $\boldsymbol{v}$ that is the derivative of a zero-mean von Mises distribution, which has a single nonlinear concentration parameter κ. The value of this parameter, $\kappa = 15.678$ms, was chosen by hand to produce results that reliably picked out the edges of groups (note that this corresponds to 6.7 hundredths of a pulse, which are the units used in the numerical calculation):

$$\boldsymbol{v}[m;\kappa] = -\kappa \sin\left(\frac{2\pi m}{N}\right) \mathrm{e}^{\kappa\cos\left(\frac{2\pi m}{N}\right)} / 2\pi I_0(\kappa)$$
$$edge(\boldsymbol{x};\kappa)_n = (|\boldsymbol{x} * \boldsymbol{v}|)_n,$$

where $*$ denotes circular convolution and I_0 is the modified Bessel function of the first kind.

- $Markov2, 3, 4, \ldots$: For each $S = 2$, 3 or 4, we define

$$MarkovS(\boldsymbol{x})_n = \frac{|\{k \mid (x_{k-S}, \ldots, x_{k-1}, x_k) = (x_{n-S}, \ldots, x_{n-1}, 1)\}|}{|\{k \mid (x_{k-S}, \ldots, x_{k-1}) = (x_{n-S}, \ldots, x_{n-1})\}|},$$

which is the cue probability at n as predicted by the maximum-likelihood description of $\boldsymbol{x}$ as an S-step Markov chain.

S. 3 Interpreting the models' coefficients

The interpretations of the models' coefficients (effects) depend on the modelled distribution of the dependent variable and any link function that is used to map that distribution's mean to a linear combination of the predictors. These are now explained.

The rhythm-level $accuracy$ model has a response variable that is beta-distributed (conditional on the predictors). The mean of the beta distribution is in the unit interval and we used a logit link function to map it to the linear predictor. In comparison to a standard linear model with no link function, this complicates the interpretation of each predictor but, in summary, given a tap accuracy of y, a coefficient β_{x} implies that a unit increase in x multiplies y by $\frac{\exp(\beta_{\mathrm{x}})}{(\exp(\beta_{\mathrm{x}})-1)y+1}$. Note, therefore, that this multiplicative increase differs according to the starting value of tap accuracy, y, and the resulting accuracy value is always in the unit interval.

As outlined earlier, all of the pulse-level models are constructed so that all selected predictors interact with *cue*. This enables us to determine the independent effect of each predictor on incorrect taps (i.e., probability of tapping within the window of a uncued pulse) and on correct taps (i.e., probability of tapping within the window of a cued pulse), as well as the difference between these two (e.g., how well the taps discriminate between uncued and cued pulses).

The distribution used for the tap_num model is beta-binomial. This is similar to the binomial distribution, but the probability of tapping at each periodic pulse randomly varies according to a beta distribution. It has a wider dispersion than the binomial distribution and fitted the data better. As with binomial, this distribution requires a link function such as logit or probit to transform the linear predictor's values into the unit interval; we used the former link function, which means the coefficients for the predictors, including the interactions with cue, have the meanings that are now explained.

The model can be written with the familiar Wilkinson and Rogers syntax for regression models as $tap\_num \sim cue * (x_1 + x_2 + \cdots)$. Using a logit link function, and assuming all continuous variables are centred, the exponentiated value of the intercept, $\exp(\beta_0)$, gives the odds of tapping incorrectly (all predictors at their mean or reference); the exponentiated sum of the intercept and the effect of cue, $\exp(\beta_0 + \beta_{\mathrm{cue}})$, gives the odds for correctly tapping (all other predictors at their mean or reference); $\exp(\beta_{\mathrm{x}})$ gives the *odds ratio* – the multiplicative change in the odds – of incorrectly tapping given a unit increase in x (all else being equal); the exponentiated effect of $\exp(\beta_{\mathrm{x}} + \beta_{\mathrm{cue:x}})$ gives the odds ratio for correctly tapping given a unit increase in x (all else being equal).

There are also useful interpretations in terms of the signal detection theory concept of *discriminability* (also known as d' or d-prime) (Milne & Herff, 2020). This is a standard quantification of how well a "signal" (here, a cue) can be "detected" (here, tapped) in the presence of noise or uncertainty. Under the assumption of uncertainty with a given standard deviation, discriminability is simply the number of standard deviations between no signal (an uncued pulse) and signal (a cued pulse) – the further apart they are, the more easily they can be discriminated (correct taps can be made). When all other predictors are at their mean or reference, $\beta_{\mathrm{cue}}/1.7$ closely corresponds to the discriminability of cues (as evidenced by tapping); while $\beta_{\mathrm{cue:x}}/1.7$ closely corresponds to the change in discriminability predicted by a unit increase in x (all else being equal). This means that the effects for the interactions with *cue* immediately indicate the importance of the associated predictor for facilitating or hindering their discriminability.

Furthermore, $\beta_0/1.7$ closely corresponds to the *tapping bias*, which is the propensity to not tap, regardless of whether or not there is a cue. Analogously, β_x gives the amount by which the bias is predicted to change given a unit increase in x. (There are alternative

definitions for bias but this is a common one, Milne & Herff, 2020.) This is also a useful quantity because it characterizes the extent to which any predictor influences the overall propensity to tap. (Note that if a probit link is used, none of the divisions by 1.7 are necessary and the coefficients correspond exactly to bias, discriminability, and their effects thereon; we use the logit link because the interpretation in terms of odds ratios is probably more interpretable.)

The models for tap_vel and tap_delta have the same structure as that for tap_num; that is, $tap\_vel \sim cue * (x_1 + x_2 + \cdots)$ and $tap\_delta \sim cue * (x_1 + x_2 + \cdots)$. However, in both cases the dependent variable is treated as normally distributed (conditional on the predictors) and no link function is required. This means the intercept, β_0, gives the predicted tap velocity or timing error for incorrect taps (when all predictors are at their mean or reference). The sum of the intercept and the effect of cue, $\beta_0 + \beta_{\text{cue}}$ gives the mean tap velocity or timing error for correct taps (when all predictors are at their mean or reference). The coefficient β_x gives the predicted change in incorrect tap velocity or timing error. Sums of coefficients of the form $\beta_{\text{cue}} + \beta_{\text{cue:x}}$ give the predicted change in correct tap velocity or timing error.

S. 4 Rhythms tested

Table S1. The 91 rhythms used in the experiment: m is the number of long IOIs; n is number of short IOIs ; K is the number of cues; N is the number of pulses; L is length of the long IOIs (in pulses); s is length of the short IOIs (in pulses); R is ratio L/s. The example IOI sequence shows the structure of the rhythm (note that in the experiment, the first cue played in each rhythm was randomly chosen).

m	n	L	s	K	N	R	Example IOI sequence
1	1	2	1	2	3	2/1	2 1
1	1	3	1	2	4	3/1	3 1
1	2	2	1	3	4	2/1	2 1 1
1	1	4	1	2	5	4/1	4 1
1	1	3	2	2	5	3/2	3 2
1	2	3	1	3	5	3/1	3 1 1
2	1	2	1	3	5	2/1	2 2 1
1	3	2	1	4	5	2/1	2 1 1 1
1	1	5	1	2	6	5/1	5 1
1	2	4	1	3	6	4/1	4 1 1
1	3	3	1	4	6	3/1	3 1 1 1
1	4	2	1	5	6	2/1	2 1 1 1 1
1	1	5	2	2	7	5/2	5 2
1	1	4	3	2	7	4/3	4 3
1	2	5	1	3	7	5/1	5 1 1
2	1	3	1	3	7	3/1	3 3 1
1	2	3	2	3	7	3/2	3 2 2
1	3	4	1	4	7	4/1	4 1 1 1
3	1	2	1	4	7	2/1	2 2 1 2
1	4	3	1	5	7	3/1	3 1 1 1 1
2	3	2	1	5	7	2/1	2 1 2 1 1
1	5	2	1	6	7	2/1	2 1 1 1 1 1
1	1	5	3	2	8	5/3	5 3
2	1	3	2	3	8	3/2	3 3 2
1	3	5	1	4	8	5/1	5 1 1 1
1	4	4	1	5	8	4/1	4 1 1 1 1
3	2	2	1	5	8	2/1	2 2 1 2 1
1	5	3	1	6	8	3/1	3 1 1 1 1 1
1	6	2	1	7	8	2/1	2 1 1 1 1 1 1
1	1	5	4	2	9	5/4	5 4
2	1	4	1	3	9	4/1	4 4 1
1	2	5	2	3	9	5/2	5 2 2
1	3	3	2	4	9	3/2	3 2 2 2
1	4	5	1	5	9	5/1	5 1 1 1 1
2	3	3	1	5	9	3/1	3 1 3 1 1
4	1	2	1	5	9	2/1	2 2 2 2 1
1	5	4	1	6	9	4/1	4 1 1 1 1 1
1	6	3	1	7	9	3/1	3 1 1 1 1 1 1
2	5	2	1	7	9	2/1	2 1 1 2 1 1 1
1	7	2	1	8	9	2/1	2 1 1 1 1 1 1 1
1	2	4	3	3	10	4/3	4 3 3
3	1	3	1	4	10	3/1	3 3 3 1
1	5	5	1	6	10	5/1	5 1 1 1 1 1
1	6	4	1	7	10	4/1	4 1 1 1 1 1 1
3	4	2	1	7	10	2/1	2 1 2 1 2 1 1
1	7	3	1	8	10	3/1	3 1 1 1 1 1 1 1
1	8	2	1	9	10	2/1	2 1 1 1 1 1 1 1 1
2	1	5	1	3	11	5/1	5 5 1
1	2	5	3	3	11	5/3	5 3 3
2	1	4	3	3	11	4/3	4 4 3
1	3	5	2	4	11	5/2	5 2 2 2
3	1	3	2	4	11	3/2	3 3 3 2
2	3	4	1	5	11	4/1	4 1 4 1 1

3	2	3	1	5	11	3/1	3 3 1 3 1
1	4	3	2	5	11	3/2	3 2 2 2 2
5	1	2	1	6	11	2/1	2 2 2 2 2 1
1	6	5	1	7	11	5/1	5 1 1 1 1 1 1
2	5	3	1	7	11	3/1	3 1 1 3 1 1 1
4	3	2	1	7	11	2/1	2 2 1 2 1 2 1
1	7	4	1	8	11	4/1	4 1 1 1 1 1 1 1
3	5	2	1	8	11	2/1	2 1 2 1 1 2 1 1
1	8	3	1	9	11	3/1	3 1 1 1 1 1 1 1 1
2	7	2	1	9	11	2/1	2 1 1 1 2 1 1 1 1
1	9	2	1	10	11	2/1	2 1 1 1 1 1 1 1 1 1
2	1	5	2	3	12	5/2	5 5 2
2	3	3	2	5	12	3/2	3 2 3 2 2
5	2	2	1	7	12	2/1	2 2 2 1 2 2 1
1	7	5	1	8	12	5/1	5 1 1 1 1 1 1 1
1	8	4	1	9	12	4/1	4 1 1 1 1 1 1 1 1
1	9	3	1	10	12	3/1	3 1 1 1 1 1 1 1 1 1
1	10	2	1	11	12	2/1	2 1 1 1 1 1 1 1 1 1 1
2	1	5	3	3	13	5/3	5 5 3
1	2	5	4	3	13	5/4	5 4 4
3	1	4	1	4	13	4/1	4 4 4 1
1	3	4	3	4	13	4/3	4 3 3 3
2	3	5	1	5	13	5/1	5 1 5 1 1
4	1	3	1	5	13	3/1	3 3 3 3 1
1	4	5	2	5	13	5/2	5 2 2 2 2
3	2	3	2	5	13	3/2	3 3 2 3 2
1	5	3	2	6	13	3/2	3 2 2 2 2 2
2	5	4	1	7	13	4/1	4 1 1 4 1 1 1
3	4	3	1	7	13	3/1	3 1 3 1 3 1 1
6	1	2	1	7	13	2/1	2 2 2 2 2 2 1
5	3	2	1	8	13	2/1	2 2 1 2 2 1 2 1
1	8	5	1	9	13	5/1	5 1 1 1 1 1 1 1 1
2	7	3	1	9	13	3/1	3 1 1 1 3 1 1 1 1
4	5	2	1	9	13	2/1	2 1 2 1 2 1 2 1 1
1	9	4	1	10	13	4/1	4 1 1 1 1 1 1 1 1 1
3	7	2	1	10	13	2/1	2 1 1 2 1 1 2 1 1 1
1	10	3	1	11	13	3/1	3 1 1 1 1 1 1 1 1 1 1
2	9	2	1	11	13	2/1	2 1 1 1 1 2 1 1 1 1 1

S. 5 Correlations of predictors

Table S2. Correlations of all predictors across performances (from the data used in the rhythm-level tap accuracy model but including all predictors, not just those selected for the reported model).

	duple_triple	N	K	mean_IOI	step_ent	all_ent	balance	evenness	CQ	SQ
duple_triple	1.00	-0.31	-0.07	-0.12	0.08	0.02	-0.22	-0.17	-0.20	-0.08
N	-0.31	1.00	0.58	0.09	-0.74	0.30	0.56	0.43	0.32	0.58
K	-0.07	0.58	1.00	-0.69	-0.73	0.80	0.44	0.37	-0.02	0.80
mean_IOI	-0.12	0.09	-0.69	1.00	0.25	-0.87	-0.11	-0.09	0.35	-0.63
step_ent	0.08	-0.74	-0.73	0.25	1.00	-0.55	-0.37	-0.32	0.02	-0.76
all_ent	0.02	0.30	0.80	-0.87	-0.55	1.00	0.37	0.28	-0.28	0.92
balance	-0.22	0.56	0.44	-0.11	-0.37	0.37	1.00	0.94	0.54	0.52
evenness	-0.17	0.43	0.37	-0.09	-0.32	0.28	0.94	1.00	0.50	0.40
CQ	-0.20	0.32	-0.02	0.35	0.02	-0.28	0.54	0.50	1.00	-0.16
SQ	-0.08	0.58	0.80	-0.63	-0.76	0.92	0.52	0.40	-0.16	1.00

Table S3. Correlations of all predictors across the *N* pulses in each rhythm (from the data used in the pulse-level tap probability model but including all predictors, not just those selected for the reported model).

	N	K	mean_IOI	evenness	balance	all_ent	step_ent	SQ	CQ	duple_triple	cue	reg_exp	proj_cent	Mean_offset	Markov2	APM	edge
N	1.00	0.51	0.07	0.43	0.53	0.25	-0.65	0.52	0.34	-0.37	-0.02	-0.03	0.00	0.00	-0.03	0.29	0.32
K	0.51	1.00	-0.75	0.32	0.37	0.82	-0.71	0.80	-0.06	-0.07	0.33	0.57	0.00	0.00	0.43	0.93	0.08
mean_IOI	0.07	-0.75	1.00	-0.06	-0.10	-0.91	0.34	-0.71	0.35	-0.13	-0.37	-0.65	0.00	0.00	-0.50	-0.77	0.09
evenness	0.43	0.32	-0.06	1.00	0.94	0.21	-0.26	0.35	0.58	-0.18	0.06	0.11	0.00	0.00	0.08	0.26	0.09
balance	0.53	0.37	-0.10	0.94	1.00	0.29	-0.25	0.45	0.64	-0.24	0.06	0.10	0.00	0.00	0.08	0.27	0.18
all_ent	0.25	0.82	-0.91	0.21	0.29	1.00	-0.55	0.92	-0.28	0.03	0.33	0.58	0.00	0.00	0.44	0.75	0.03
step_ent	-0.65	-0.71	0.34	-0.26	-0.25	-0.55	1.00	-0.71	0.08	0.05	-0.17	-0.29	0.00	0.00	-0.22	-0.61	-0.08
SQ	0.52	0.80	-0.71	0.35	0.45	0.92	-0.71	1.00	-0.15	-0.08	0.26	0.46	0.00	0.00	0.35	0.68	0.13
CQ	0.34	-0.06	0.35	0.58	0.64	-0.28	0.08	-0.15	1.00	-0.22	-0.13	-0.22	0.00	0.00	-0.17	-0.13	0.15
duple_triple	-0.37	-0.07	-0.13	-0.18	-0.24	0.03	0.05	-0.08	-0.22	1.00	0.06	0.11	0.00	0.00	0.09	0.03	-0.15
cue	-0.02	0.33	-0.37	0.06	0.06	0.33	-0.17	0.26	-0.13	0.06	1.00	0.11	0.42	0.00	0.75	0.54	-0.15
reg_exp	-0.03	0.57	-0.65	0.11	0.10	0.58	-0.29	0.46	-0.22	0.11	0.11	1.00	0.16	0.54	0.20	0.57	-0.13
proj_cent	0.00	0.00	0.00	0.00	0.00	0.00	0.00	0.00	0.00	0.00	0.42	0.16	1.00	0.00	0.21	0.08	-0.16
mean_offset	0.00	0.00	0.00	0.00	0.00	0.00	0.00	0.00	0.00	0.00	0.00	0.54	0.00	1.00	-0.03	0.00	0.01
Markov2	-0.03	0.43	-0.50	0.08	0.08	0.44	-0.22	0.35	-0.17	0.09	0.75	0.20	0.21	-0.03	1.00	0.58	-0.12
APM	0.29	0.93	-0.77	0.26	0.27	0.75	-0.61	0.68	-0.13	0.03	0.54	0.57	0.08	0.00	0.58	1.00	-0.01
edge	0.32	0.08	0.09	0.09	0.18	0.03	-0.08	0.13	0.15	-0.15	-0.15	-0.13	-0.16	0.01	-0.12	-0.01	1.00

Table S4. Correlations of all predictors across the all tapped pulses (from the data used in the pulse-level tap velocity and timing error models but including all predictors, not just those selected for the reported model).

	N	K	mean_IOI	evenness	balance	all_ent	step_ent	SQ	CQ	duple_triple	cue	reg_exp	proj_cent	Mean_offset	Markov2	APM	edge
N	1.00	0.66	0.06	0.39	0.52	0.35	-0.74	0.61	0.36	-0.30	-0.11	0.02	-0.29	-0.01	-0.06	0.44	0.33
K	0.66	1.00	-0.64	0.42	0.47	0.77	-0.76	0.80	0.09	-0.09	0.12	0.55	-0.26	0.00	0.28	0.94	0.13
mean_IOI	0.06	-0.64	1.00	-0.17	-0.16	-0.85	0.27	-0.60	0.29	-0.14	-0.26	-0.68	0.09	-0.01	-0.45	-0.72	0.09
evenness	0.39	0.42	-0.17	1.00	0.94	0.33	-0.29	0.43	0.54	-0.16	-0.07	0.12	-0.58	0.01	0.08	0.36	0.09
balance	0.52	0.47	-0.16	0.94	1.00	0.39	-0.34	0.53	0.59	-0.22	-0.09	0.09	-0.58	0.01	0.05	0.37	0.21
all_ent	0.35	0.77	-0.85	0.33	0.39	1.00	-0.58	0.91	-0.21	0.02	0.18	0.59	-0.24	0.01	0.34	0.73	0.08
step_ent	-0.74	-0.76	0.27	-0.29	-0.34	-0.58	1.00	-0.78	-0.02	0.06	-0.04	-0.31	0.22	0.01	-0.09	-0.66	-0.09
SQ	0.61	0.80	-0.60	0.43	0.53	0.91	-0.78	1.00	-0.08	-0.07	0.09	0.46	-0.32	0.00	0.21	0.69	0.17
CQ	0.36	0.09	0.29	0.54	0.59	-0.21	-0.02	-0.08	1.00	-0.20	-0.17	-0.25	-0.30	0.00	-0.10	-0.01	0.18
duple_triple	-0.30	-0.09	-0.14	-0.16	-0.22	0.02	0.06	-0.07	-0.20	1.00	0.11	0.12	0.12	-0.01	0.10	0.03	-0.15
cue	-0.11	0.12	-0.26	-0.07	-0.09	0.18	-0.04	0.09	-0.17	0.11	1.00	0.04	0.29	0.04	0.62	0.31	-0.16
reg_exp	0.02	0.55	-0.68	0.12	0.09	0.59	-0.31	0.46	-0.25	0.12	0.04	1.00	0.08	0.53	0.12	0.59	-0.17
proj_cent	-0.29	-0.26	0.09	-0.58	-0.58	-0.24	0.22	-0.32	-0.30	0.12	0.29	0.08	1.00	0.01	0.09	-0.20	-0.34
mean_offset	-0.01	0.00	-0.01	0.01	0.01	0.01	0.01	0.00	0.00	-0.01	0.04	0.53	0.01	1.00	-0.08	0.01	0.02
Markov2	-0.06	0.28	-0.45	0.08	0.05	0.34	-0.09	0.21	-0.10	0.10	0.62	0.12	0.09	-0.08	1.00	0.40	-0.20
APM	0.44	0.94	-0.72	0.36	0.37	0.73	-0.66	0.69	-0.01	0.03	0.31	0.59	-0.20	0.01	0.40	1.00	0.05
edge	0.33	0.13	0.09	0.09	0.21	0.08	-0.09	0.17	0.18	-0.15	-0.16	-0.17	-0.34	0.02	-0.20	0.05	1.00

S. 6 Circular descriptive plot calculations

The circular descriptive plots in the main paper were created with the following method. The tap-data is expressed as a vector $\boldsymbol{y} = (y_1, y_2, \dots)$, whose elements are indexed by t milliseconds and whose entries are the normalized tap velocity (MIDI velocity values are integers from 0 to 127; these were normalized by dividing by 127; a value of 0 corresponds to no tap). For each 30-second performance, the vectors were divided into segments of the length of the rhythmic period, which is $234N$ ms. The different segments were summed and multiplied by N to account for the differing numbers of repetitions of rhythms with differing N over the 30-second performance. For each rhythm, these $234N$-entry vectors were aligned, and then averaged across all performances (remember that for each presentation, rhythms are started with a random phase, which makes it possible to average across them to gain an understanding of how tap velocities and timings vary regardless of start-phase). This results in the distribution of tap velocities over the rhythmic period; in order to approximate the underlying shape of this distribution and make it easier to see, the data is smoothed by convolution with a truncated discrete normal distribution with a standard deviation of 10 ms.

S. 7 Smoothed velocity distributions for all 91 rhythms

Solid radii mark cued pulses; dashed radii mark uncued pulses. The data are normalized for each rhythm.

1 1 2 1

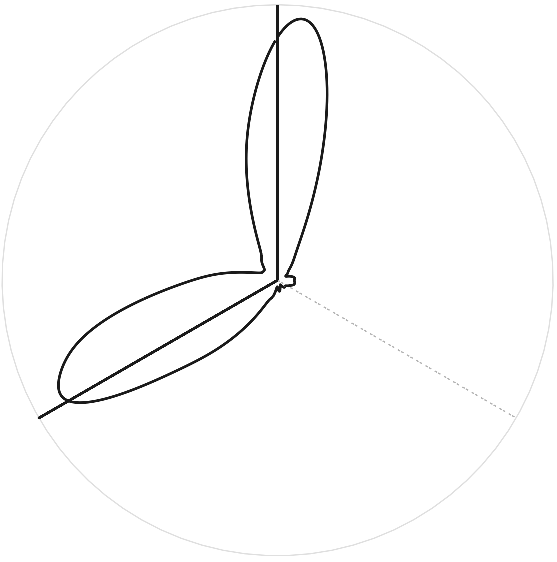

1 1 3 1

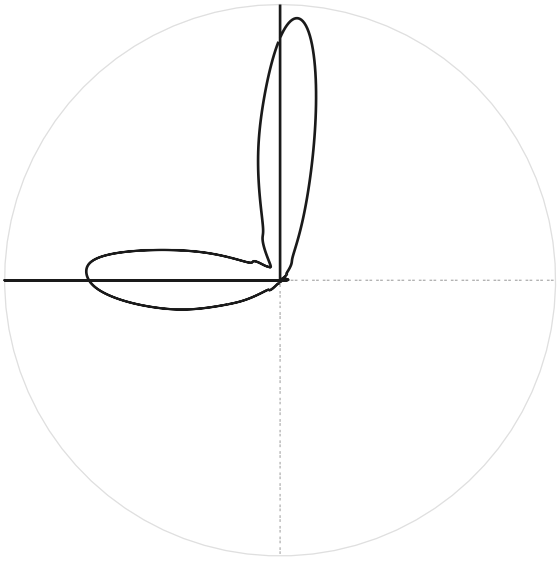

1 1 3 2

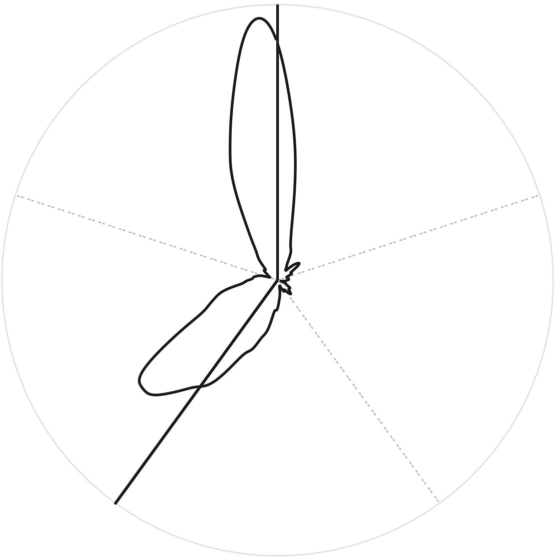

1 1 4 1

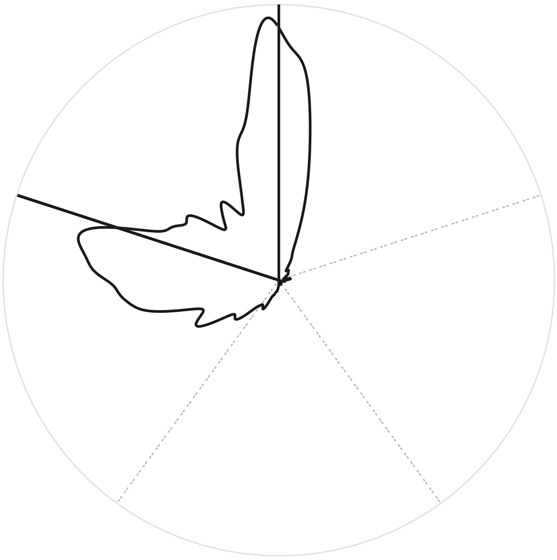

1 1 4 3

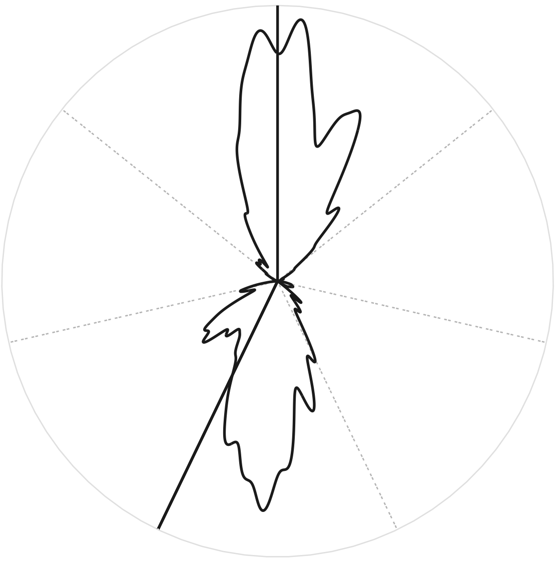

1 1 5 1

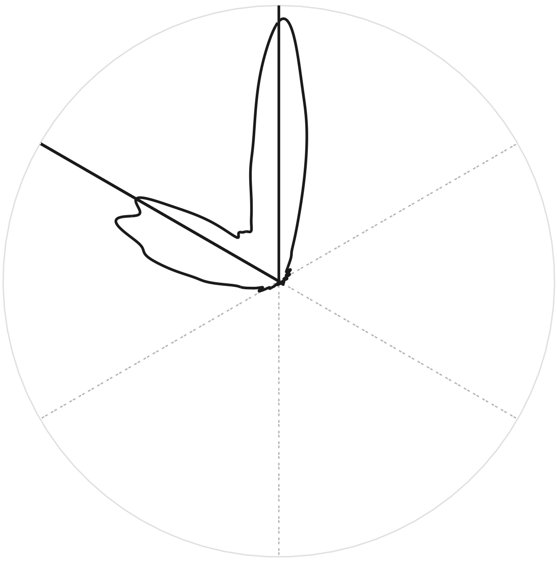

1 1 5 2

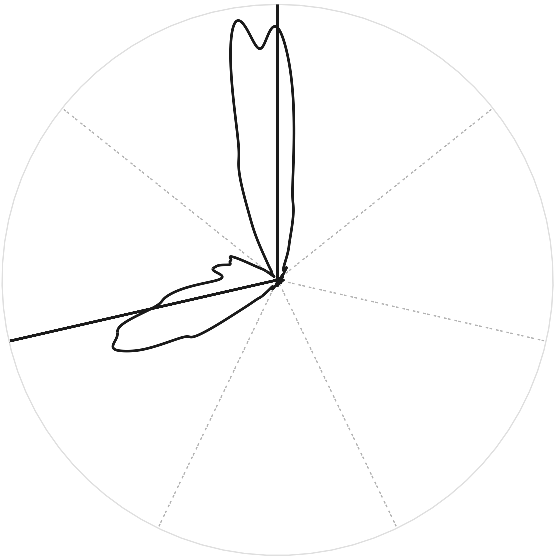

1 1 5 3

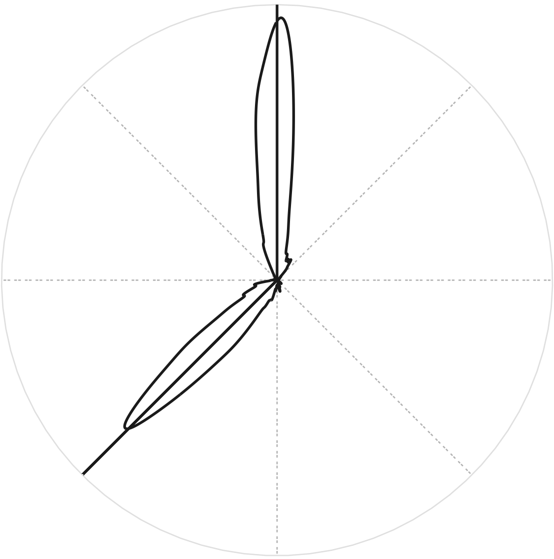

1 1 5 4

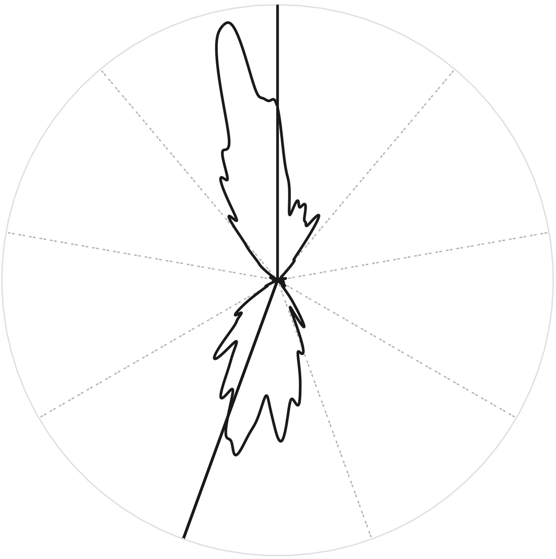

1 2 2 1

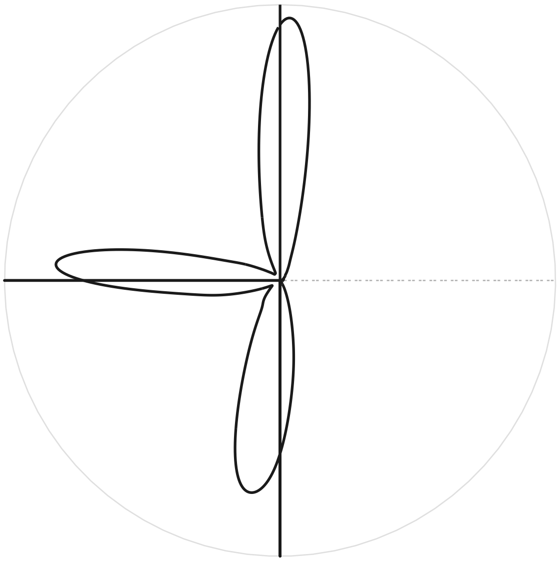

1 2 3 1

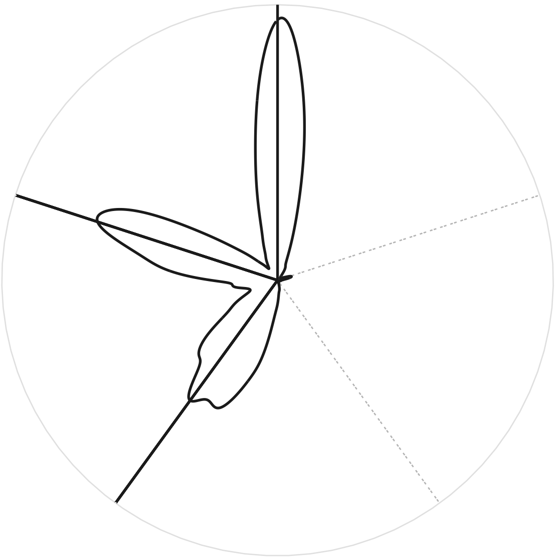

1 2 3 2

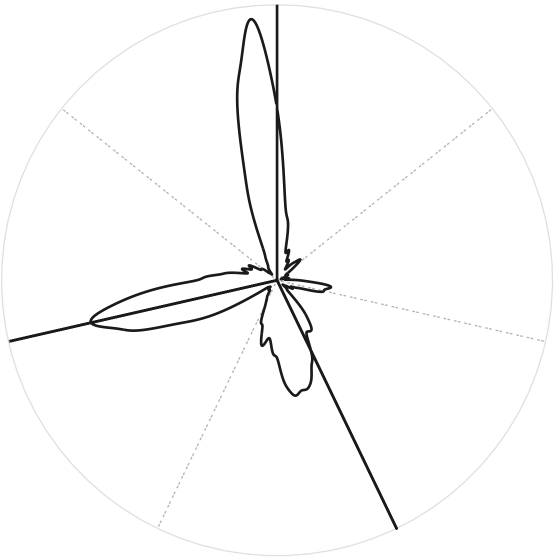

1 2 4 1

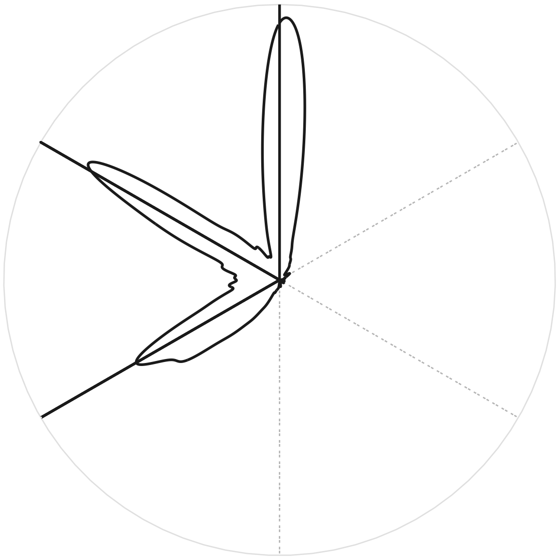

1 2 4 3

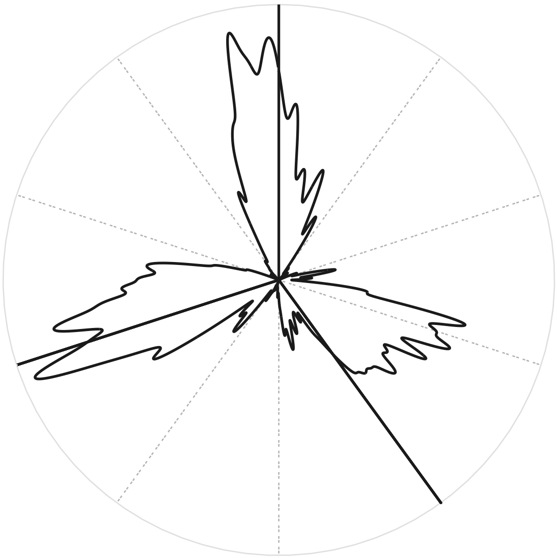

1 2 5 1

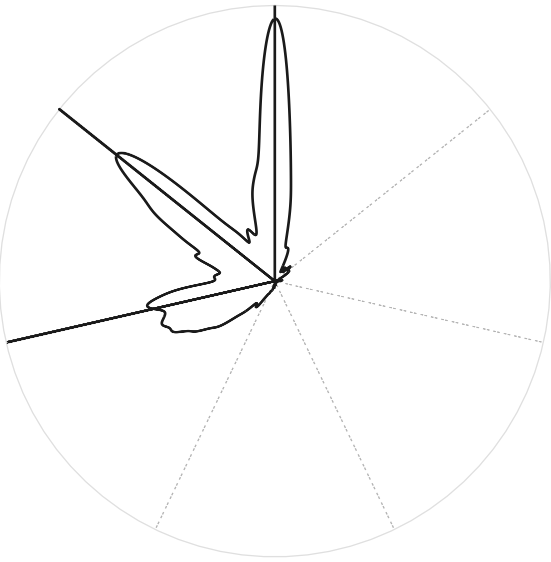

1 2 5 2

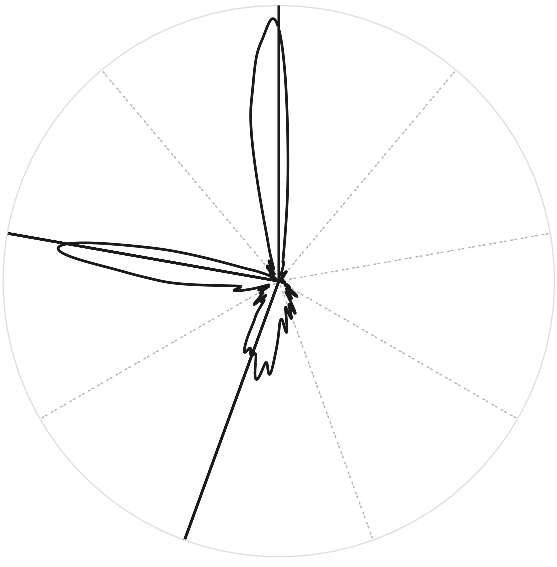

1 2 5 3

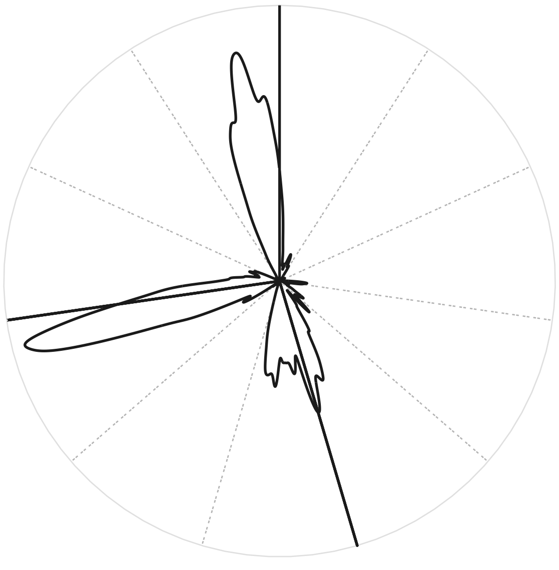

1 2 5 4

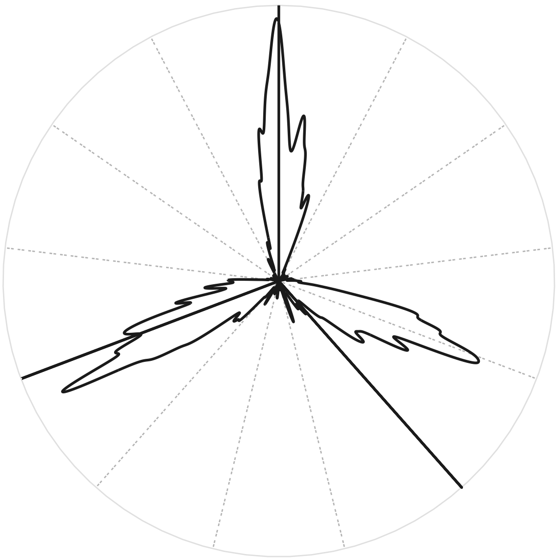

1 3 2 1

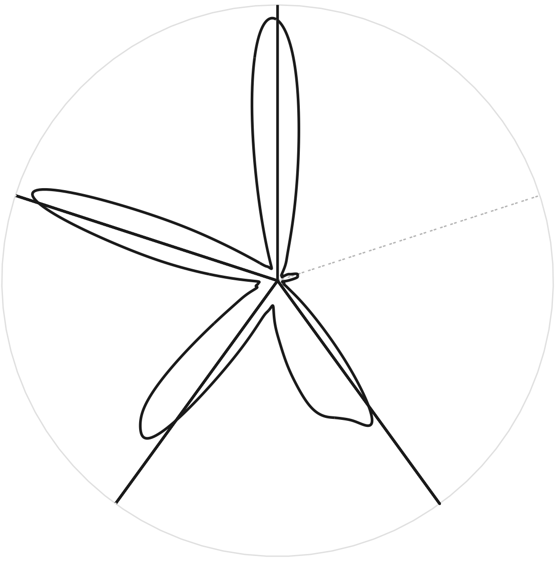

1 3 3 1

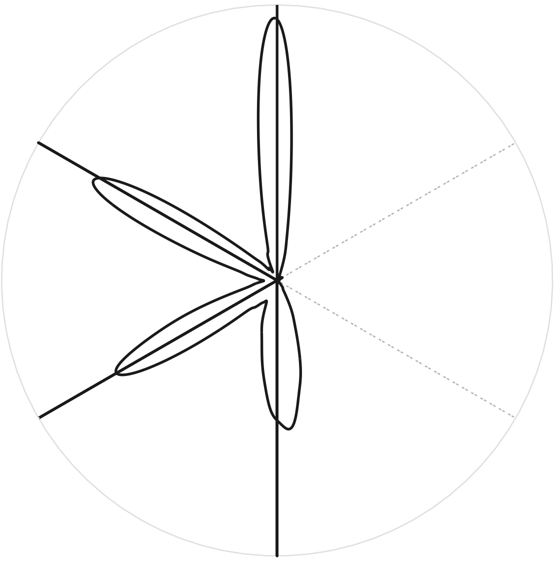

1 3 3 2

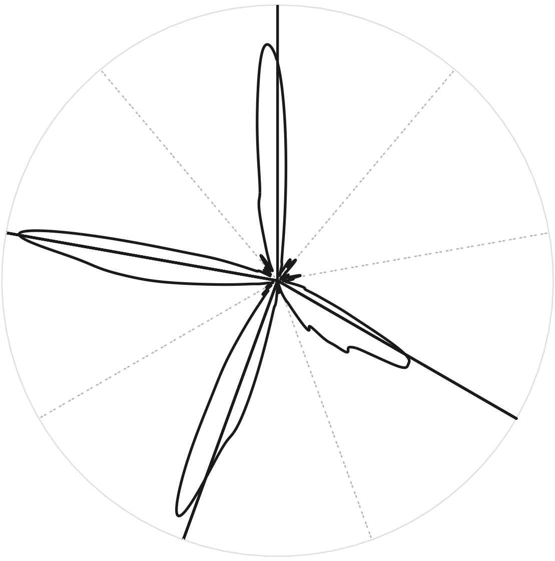

1 3 4 1

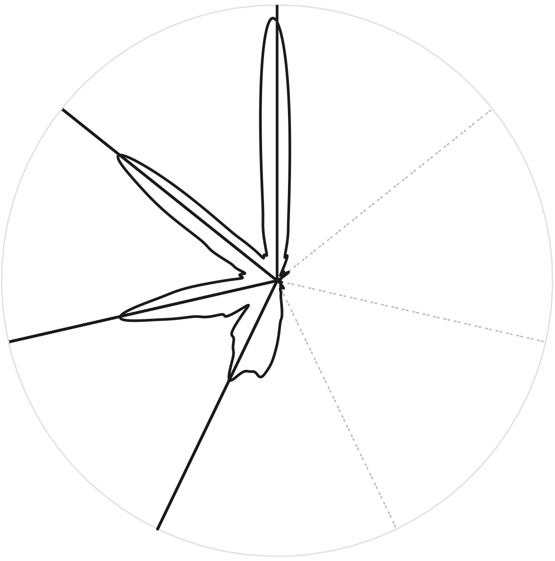

1 3 4 3

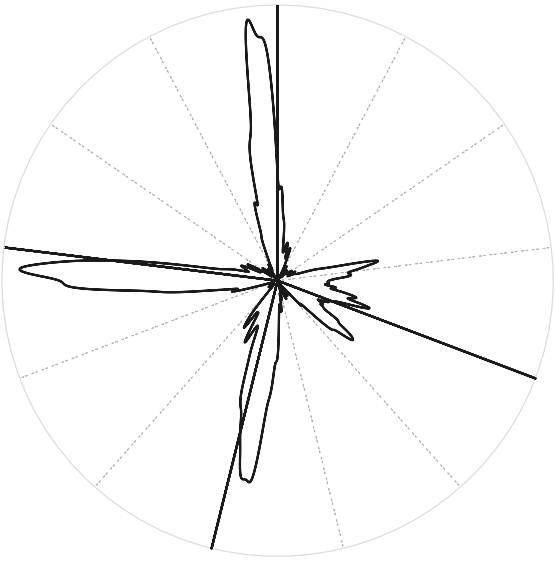

1 3 5 1

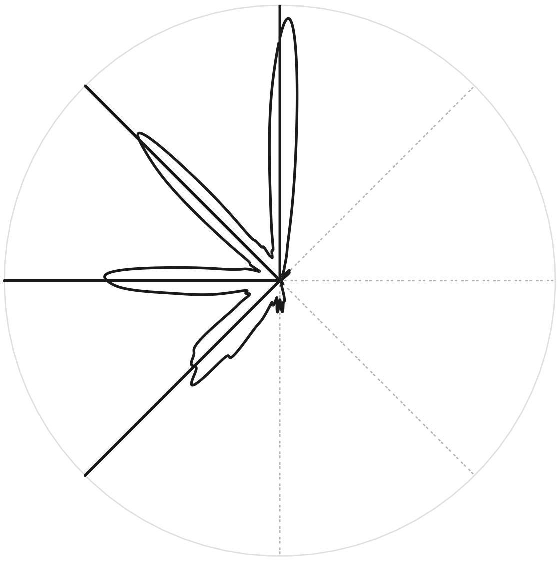

1 3 5 2

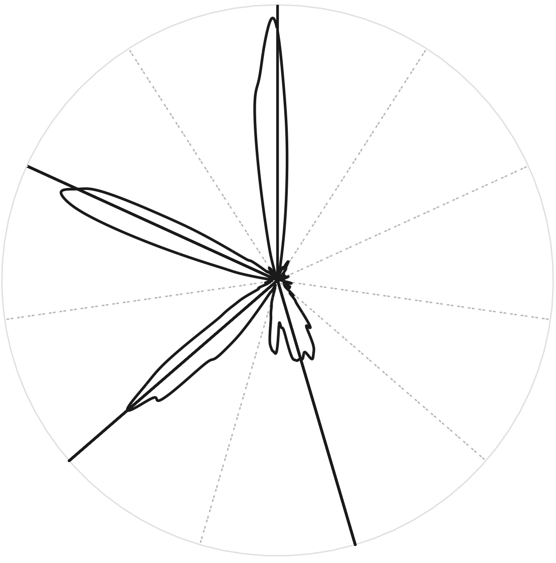

1 4 2 1

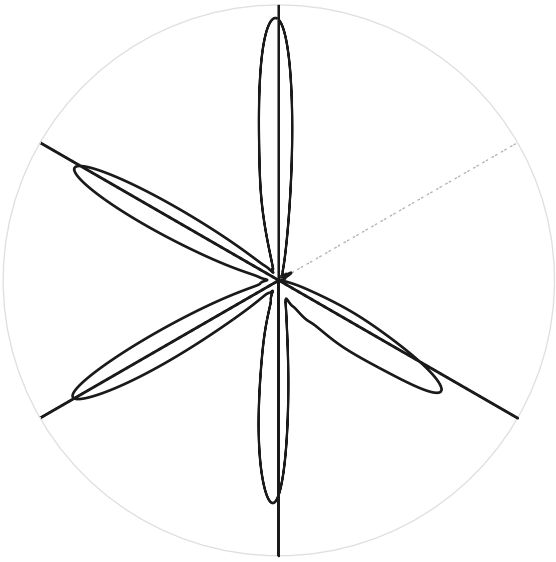

1 4 3 1

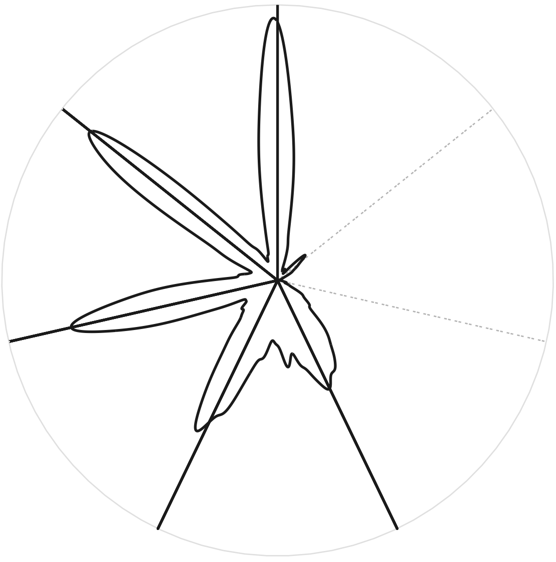

1 4 3 2

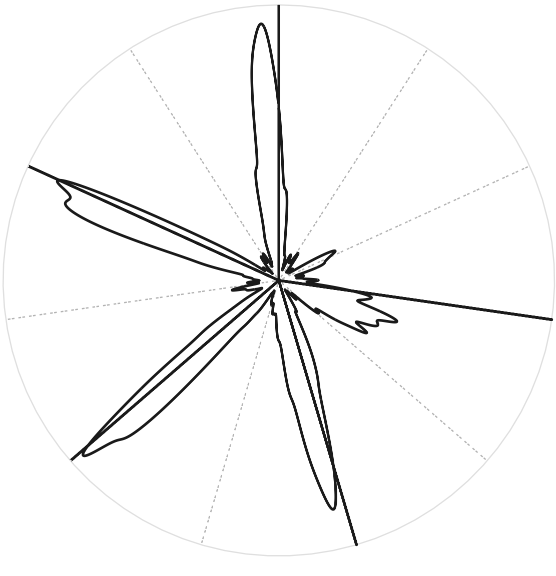

1 4 4 1

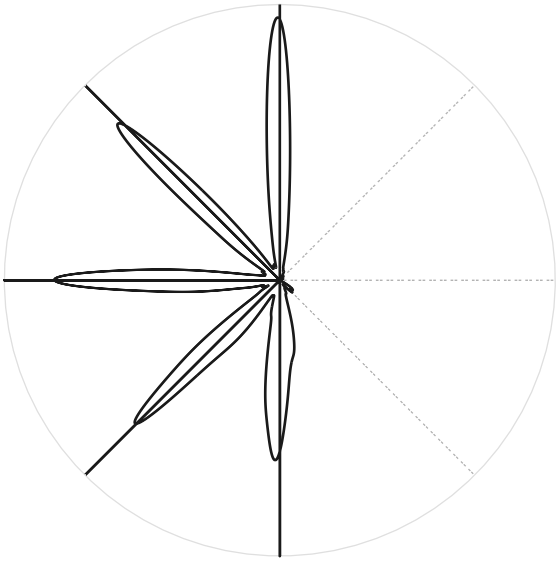

1 4 5 1

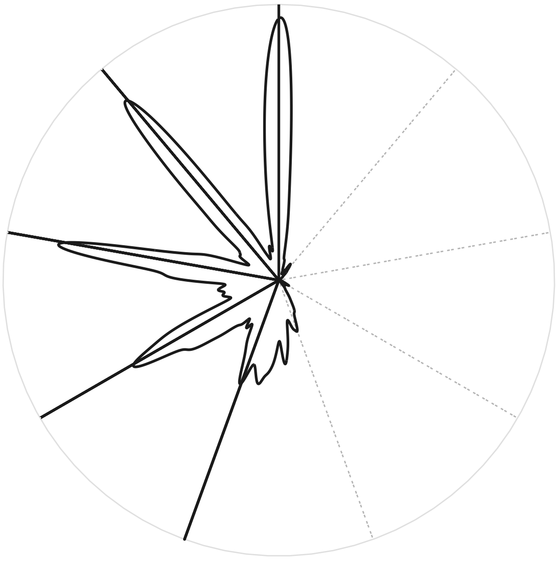

1 4 5 2

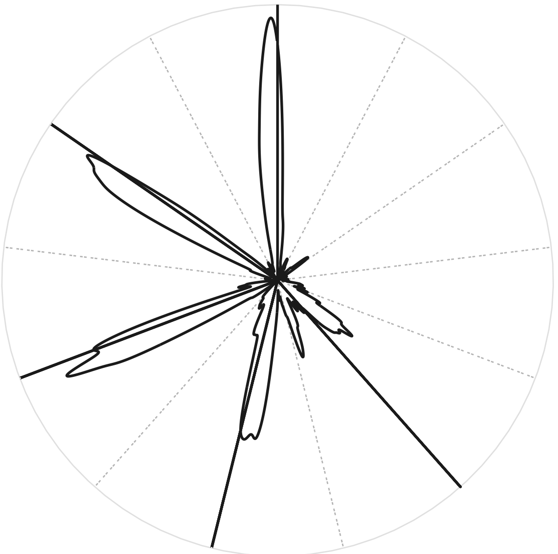

1 5 2 1

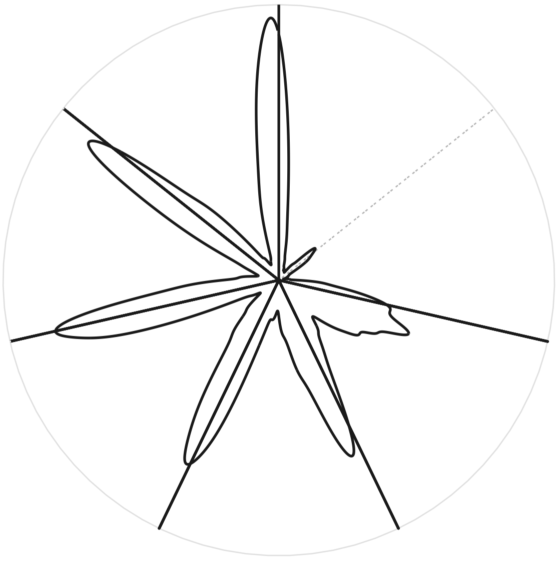

1 5 3 1

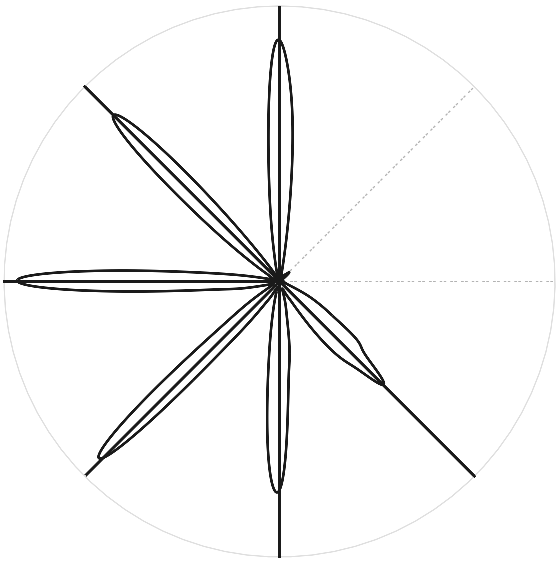

1 5 3 2

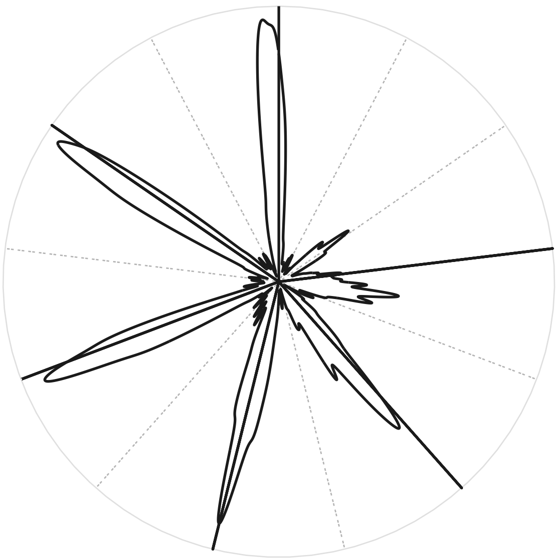

1 5 4 1

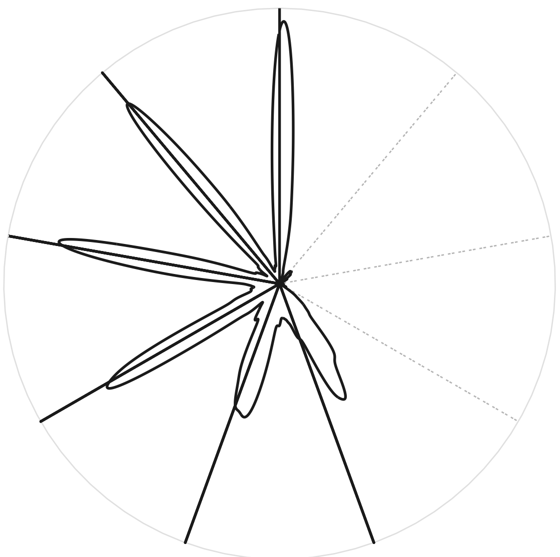

1 5 5 1

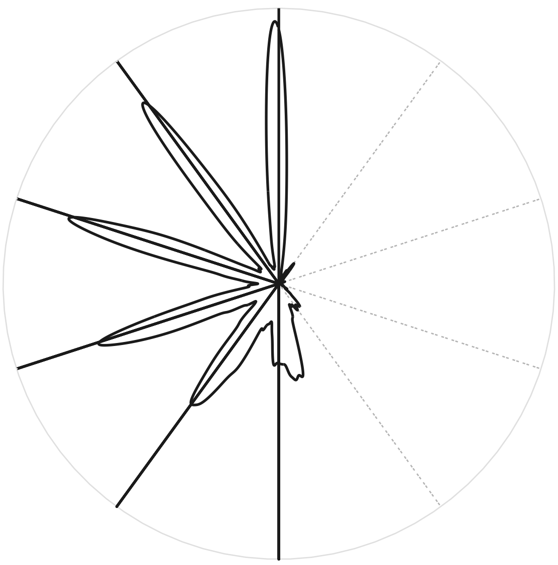

1621

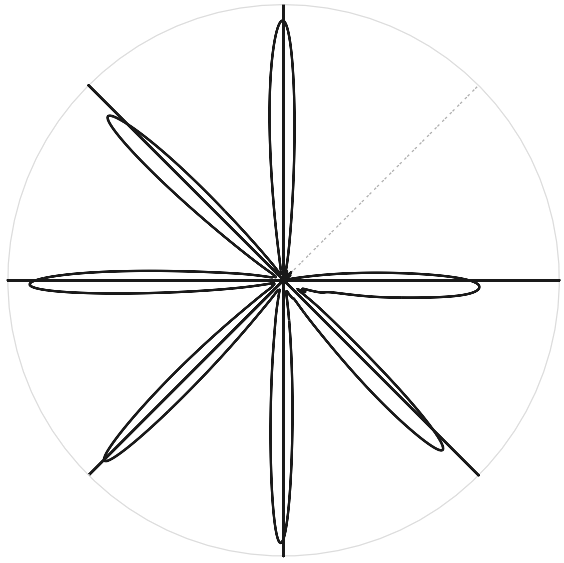

1631

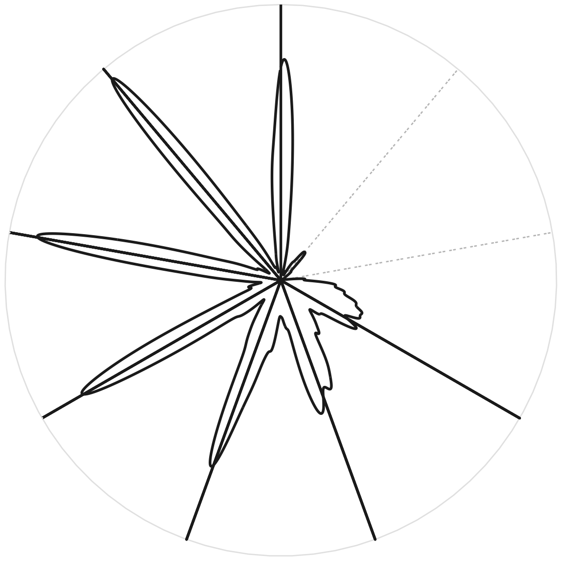

1641

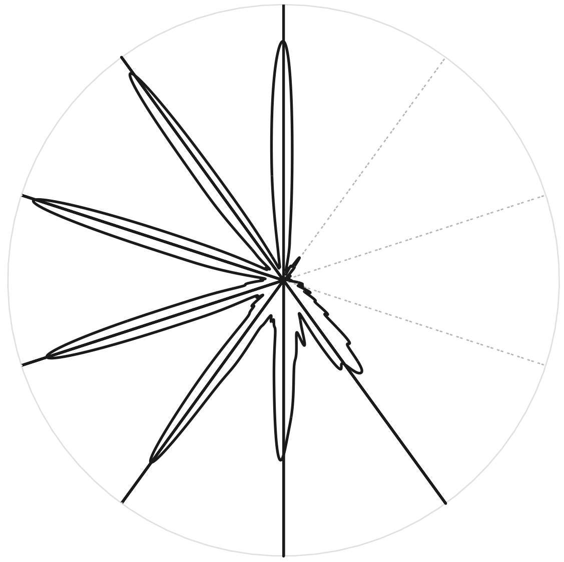

1651

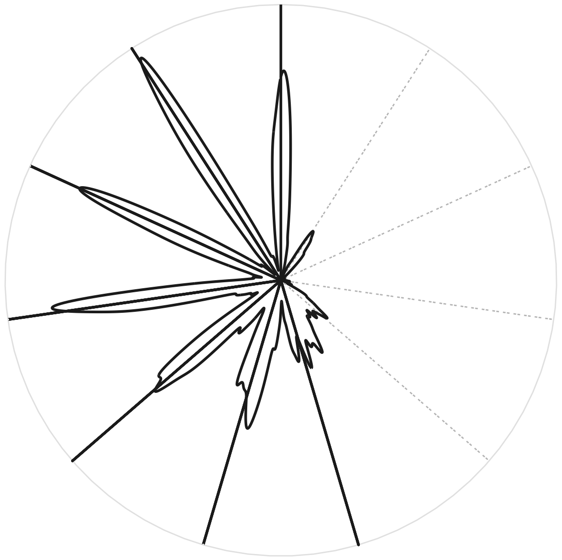

1721

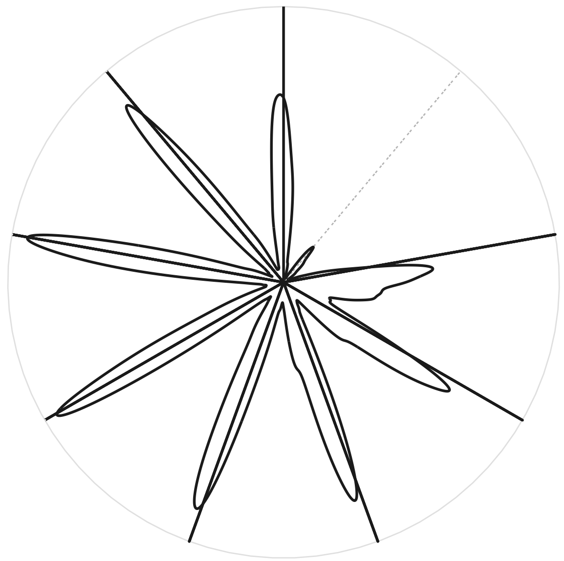

1731

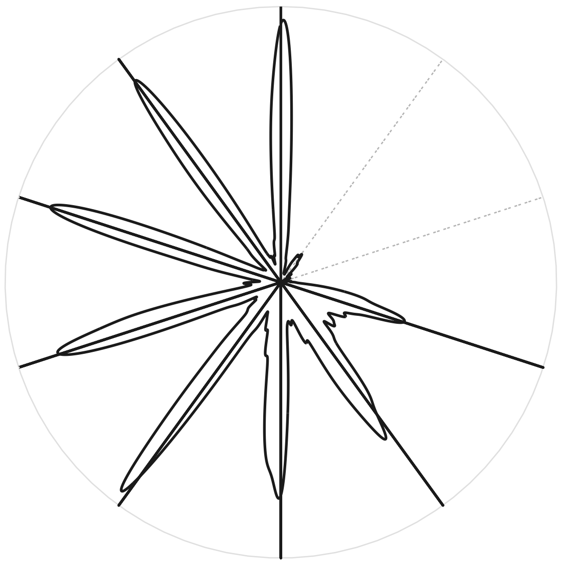

1741

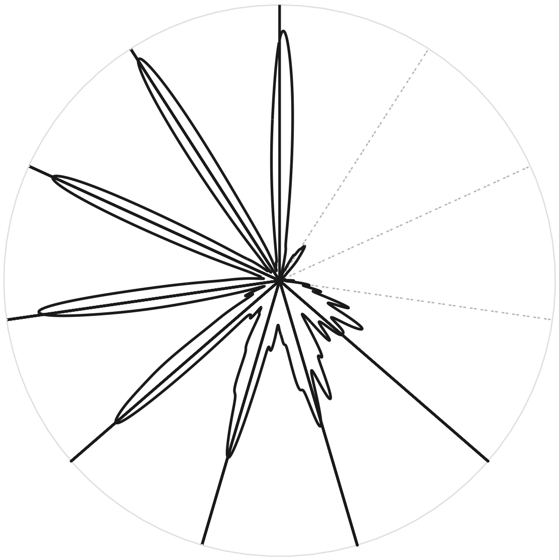

1751

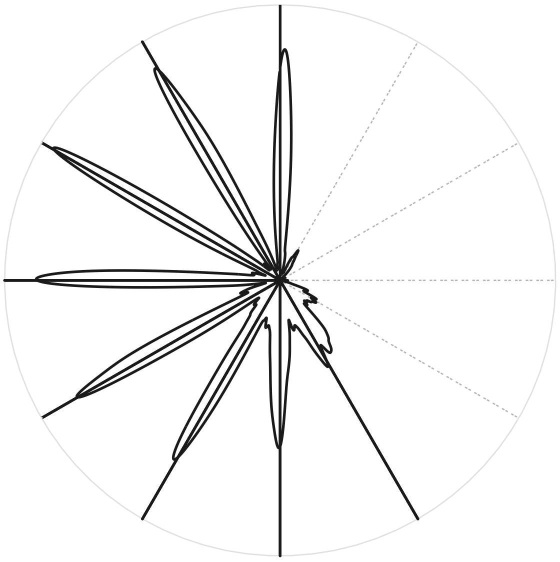

1821

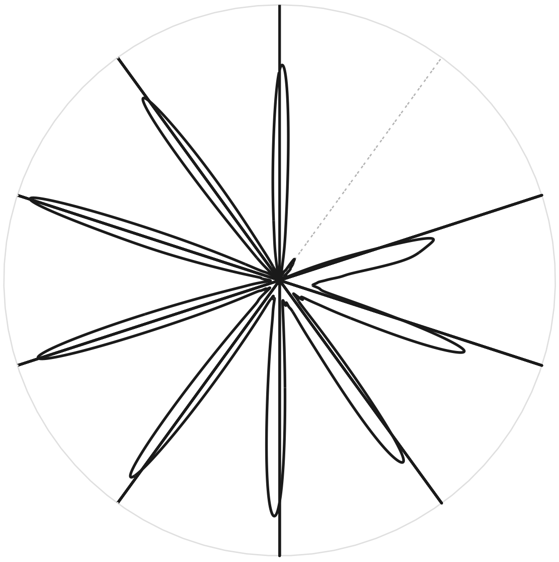

1831

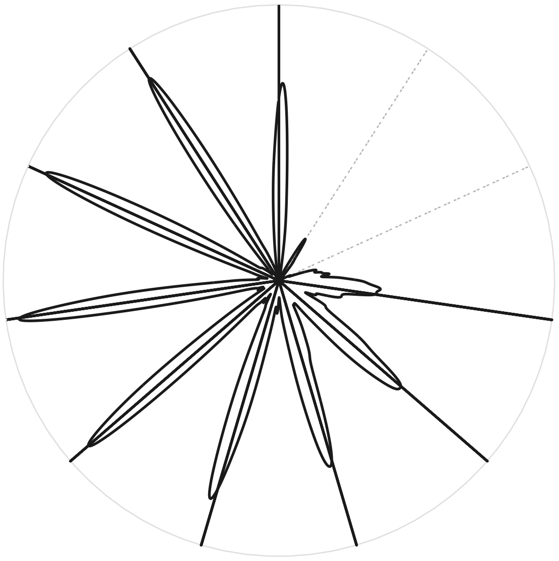

1841

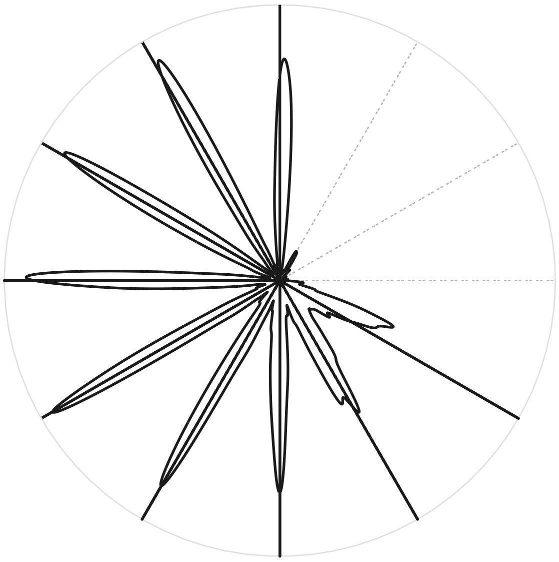

1851

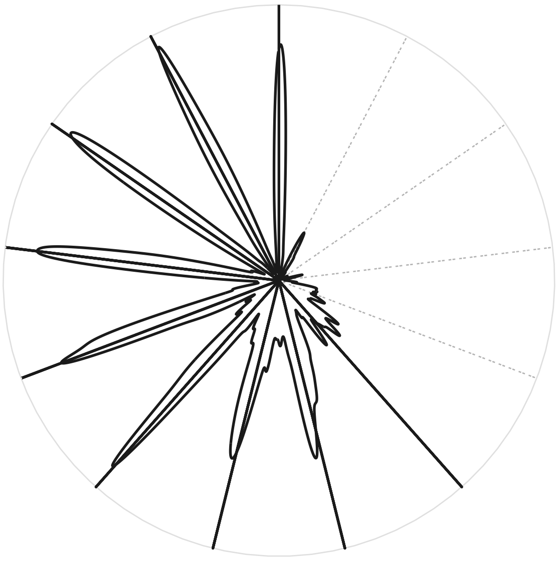

1 9 2 1

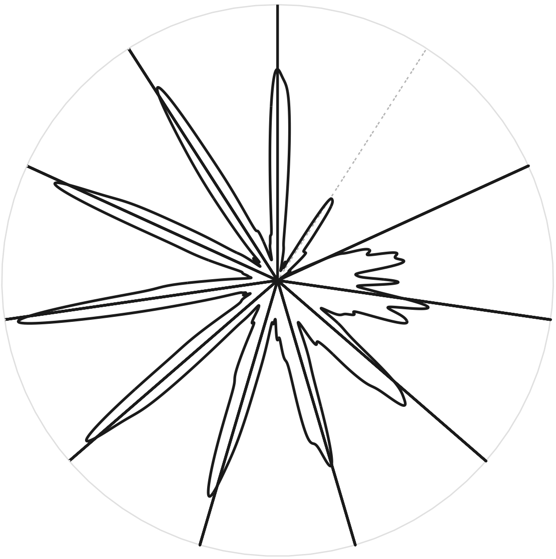

1 9 3 1

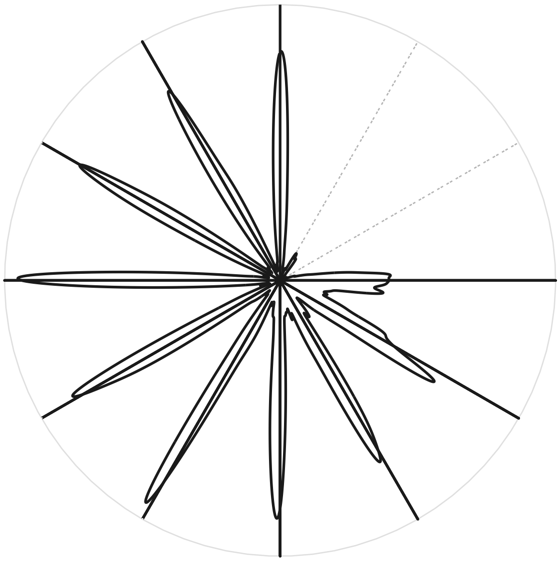

1 9 4 1

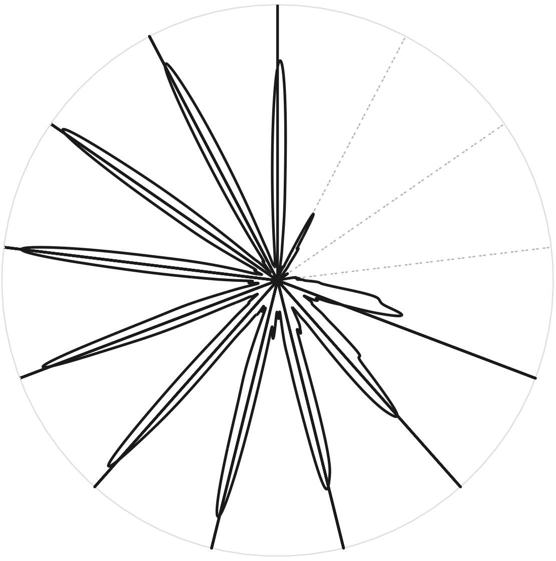

1 10 2 1

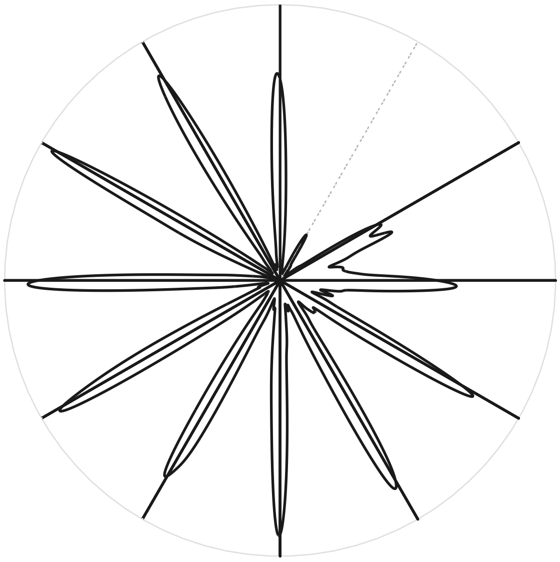

1 10 3 1

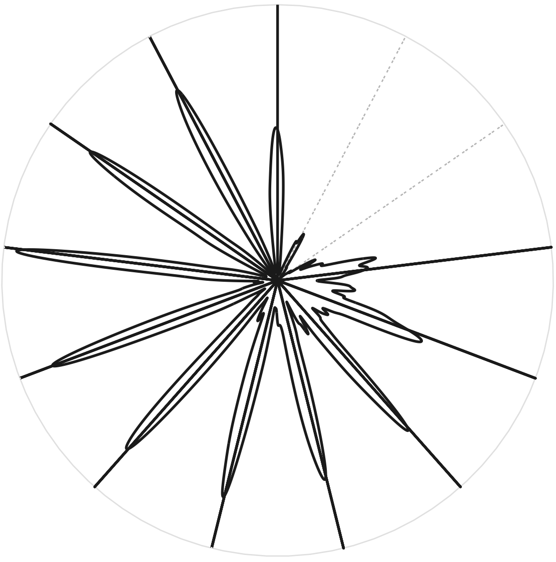

2 1 2 1

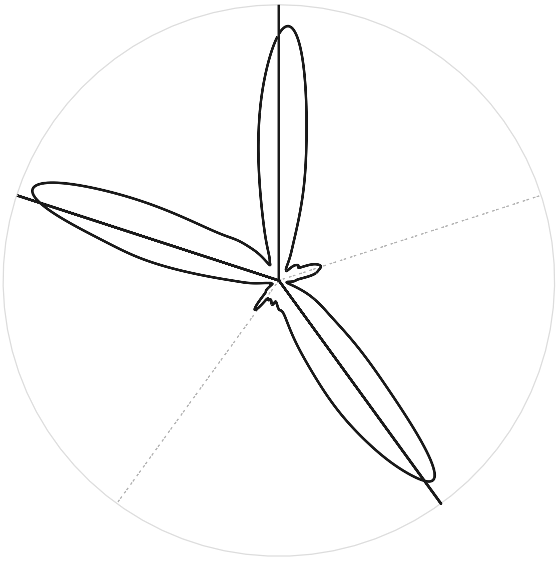

2 1 3 1

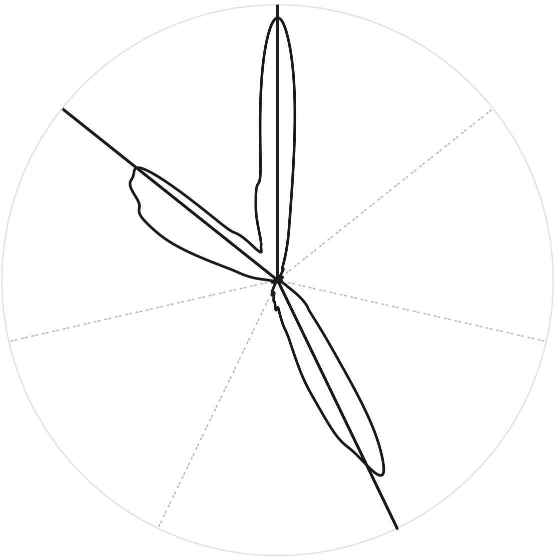

2 1 3 2

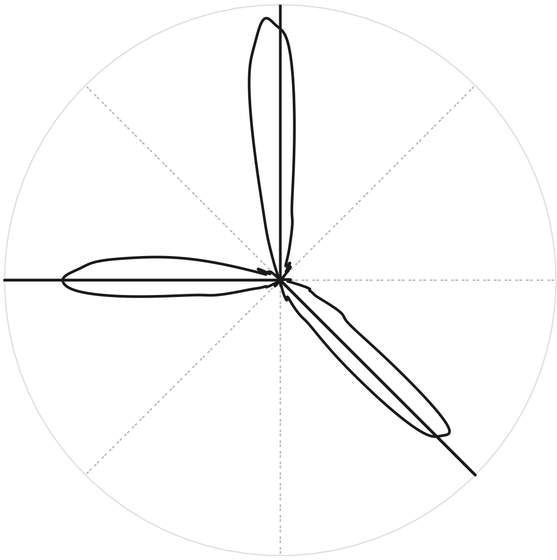

2 1 4 1

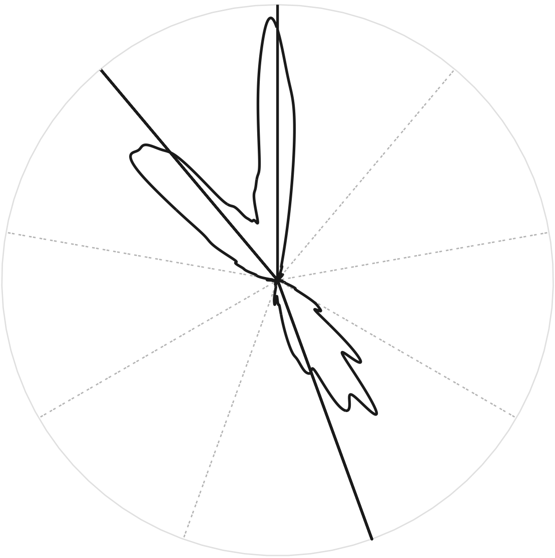

2 1 4 3

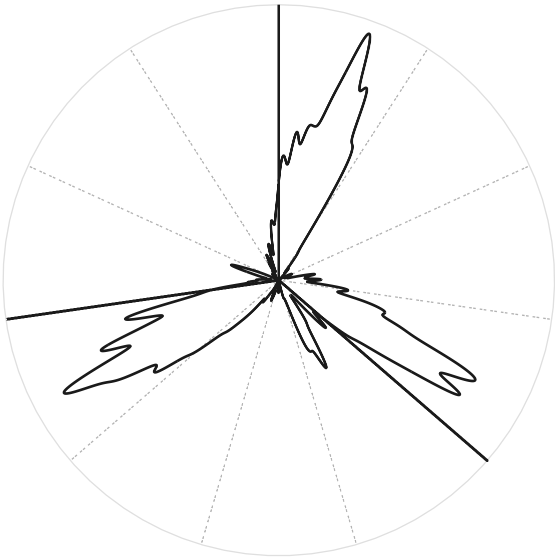

2 1 5 1

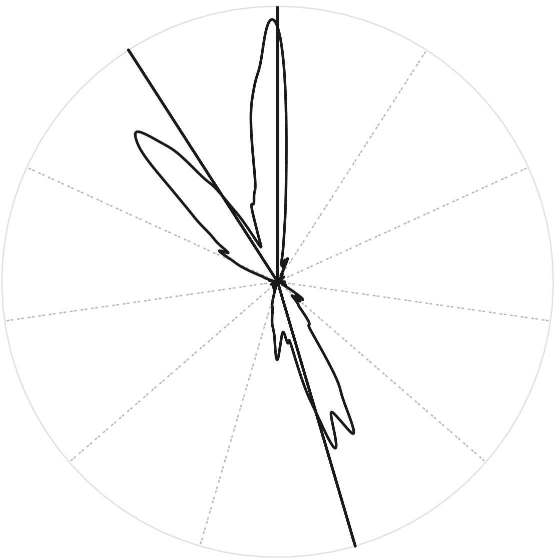

2 1 5 2

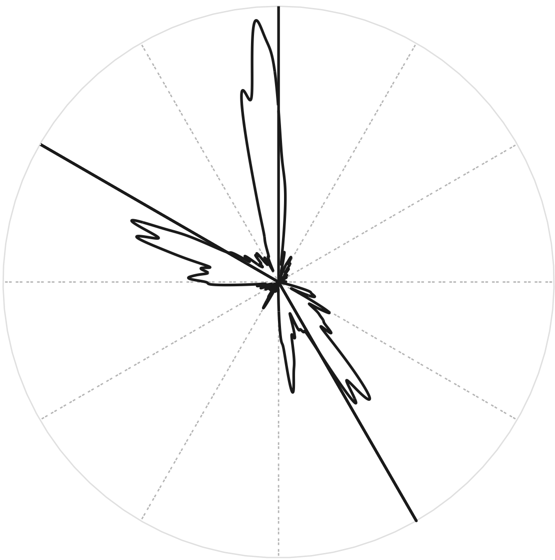

2 1 5 3

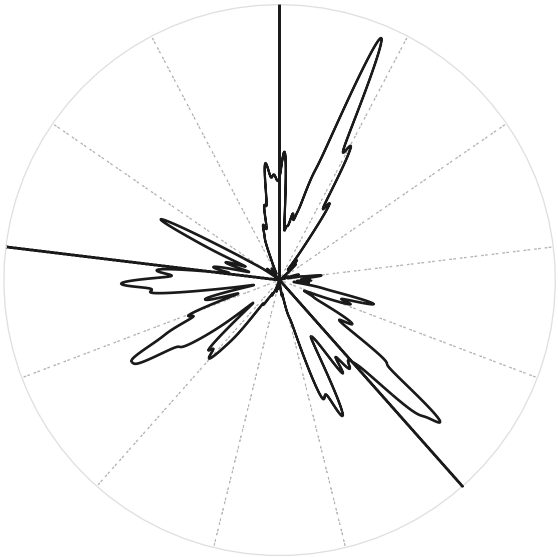

2 3 2 1

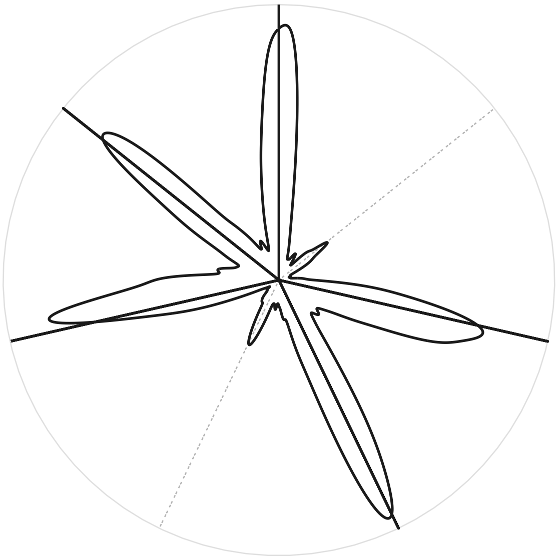

2 3 3 1

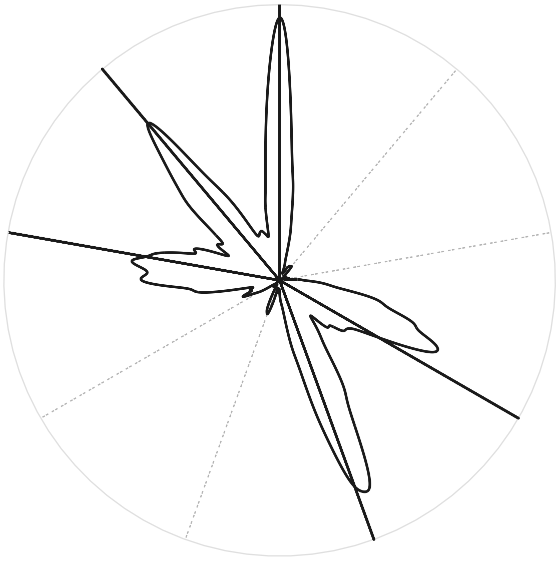

2 3 3 2

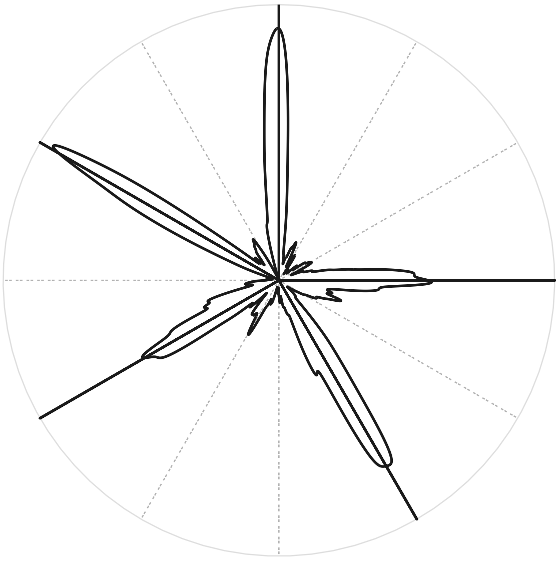

2 3 4 1

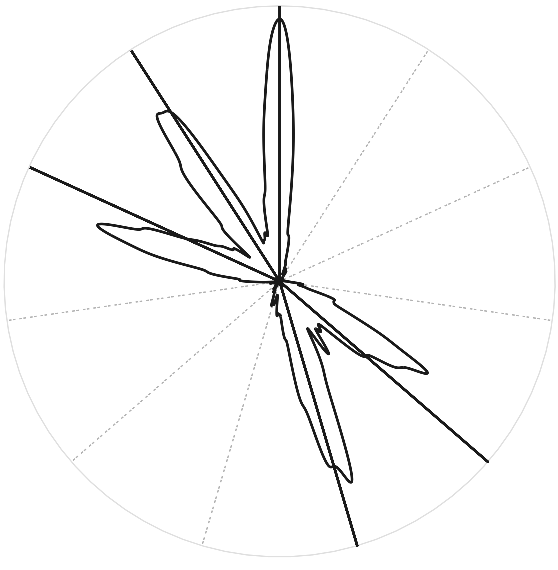

2 3 5 1

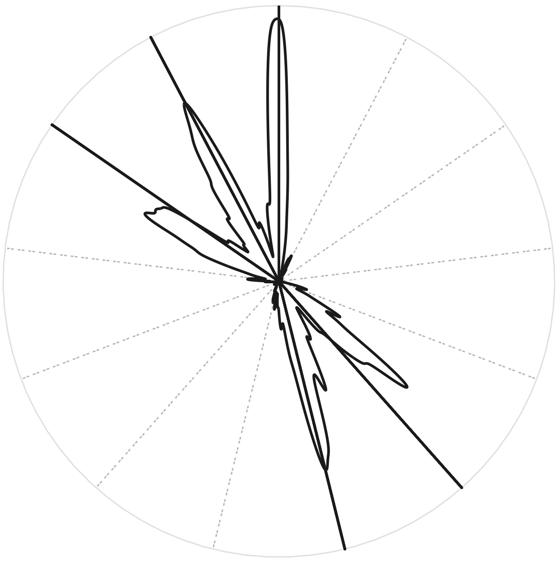

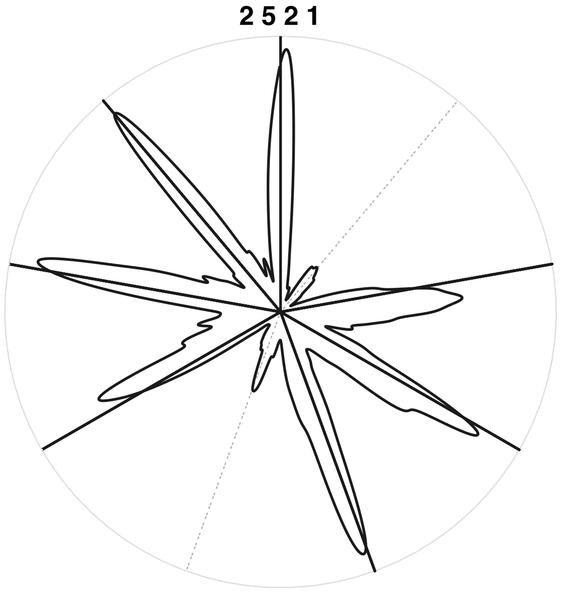
2 5 2 1

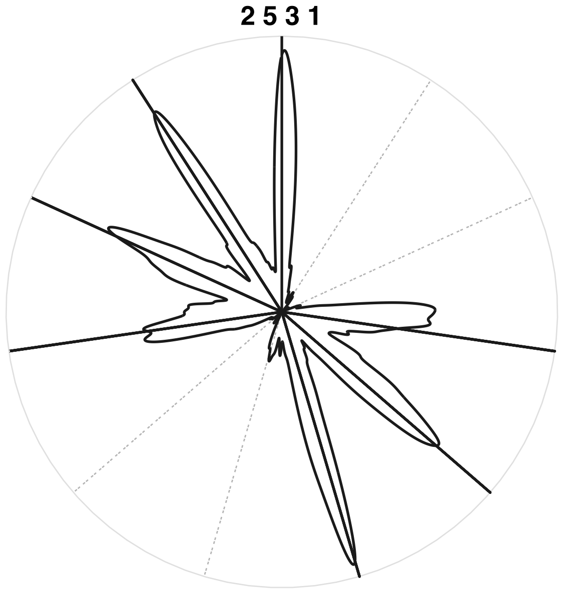
2 5 3 1

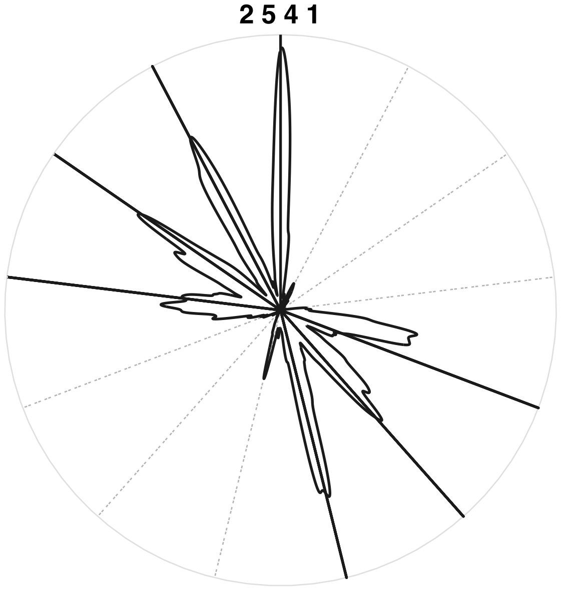
2 5 4 1

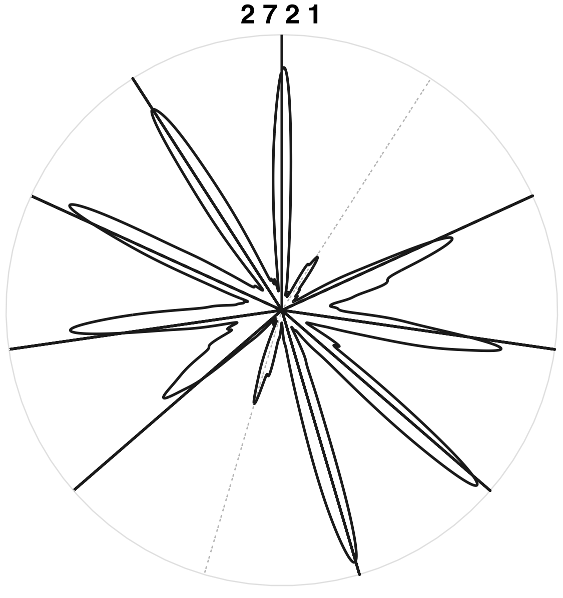
2 7 2 1

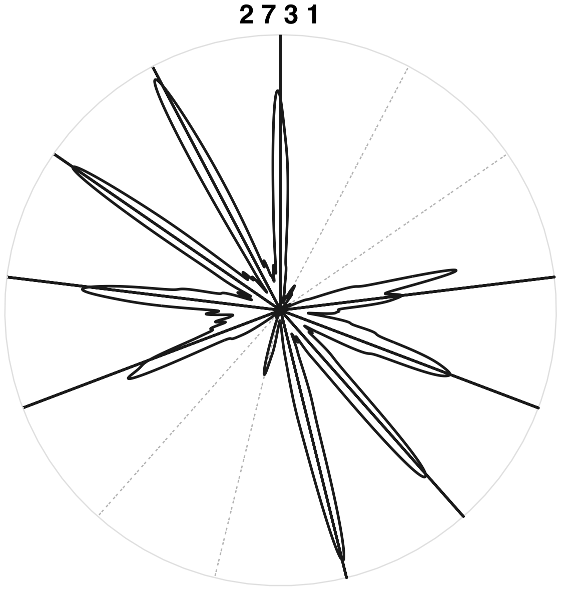
2 7 3 1

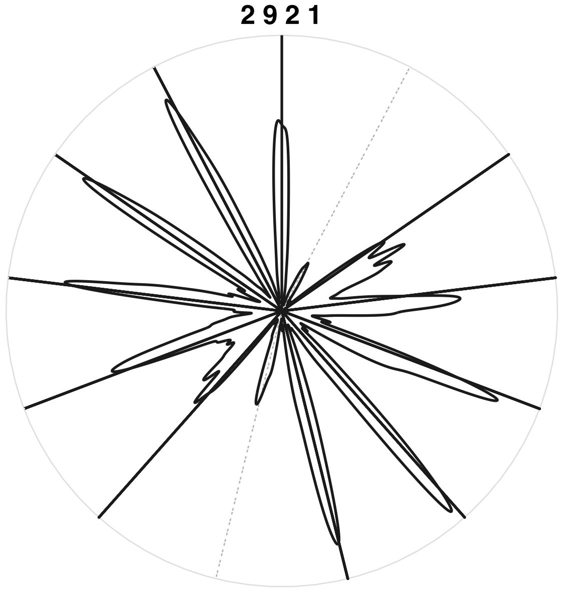
2 9 2 1

3 1 2 1

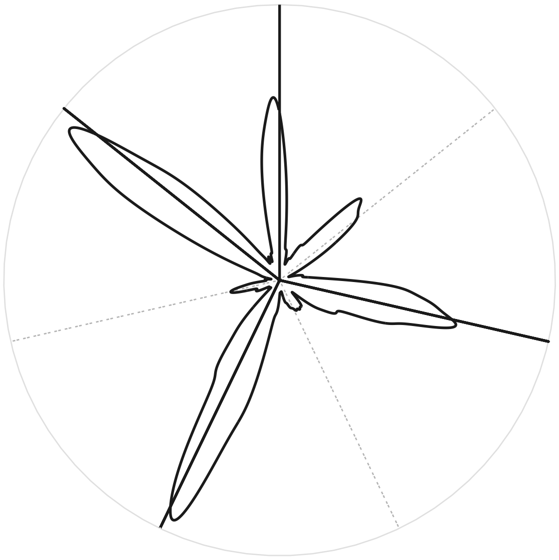

3 1 3 1

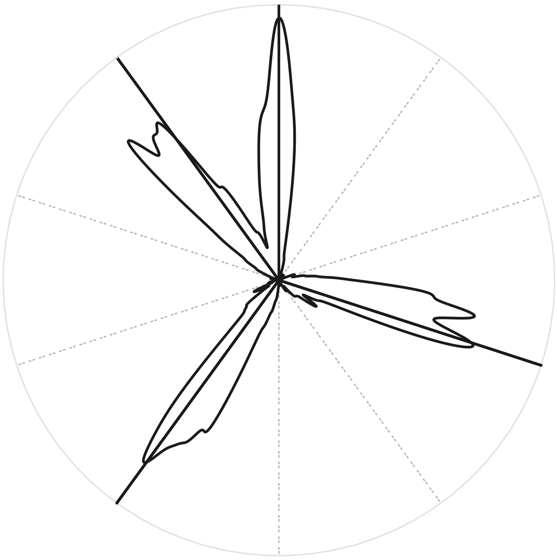

3 1 3 2

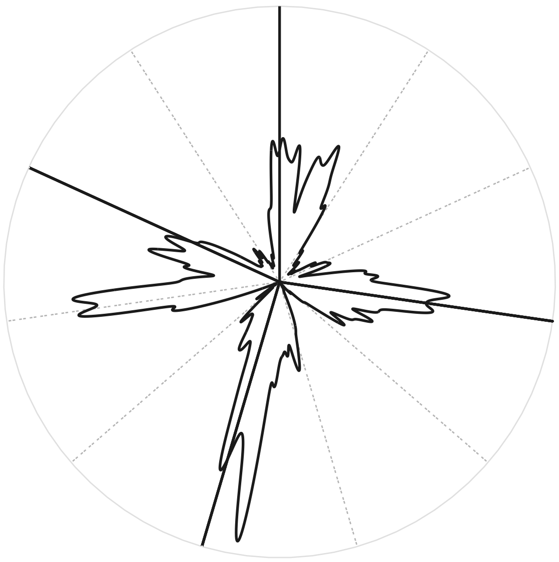

3 1 4 1

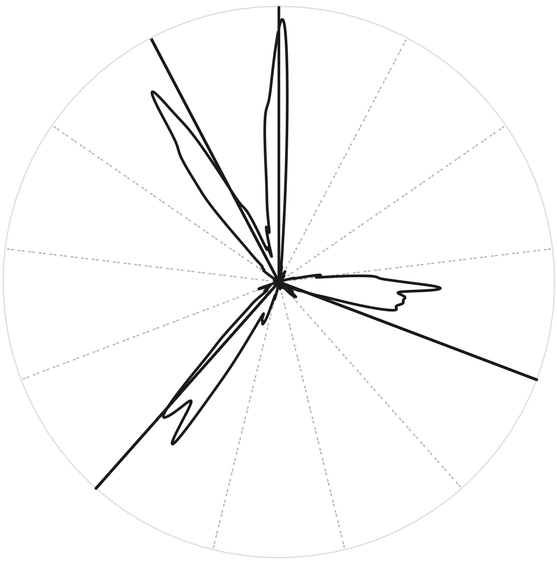

3 2 2 1

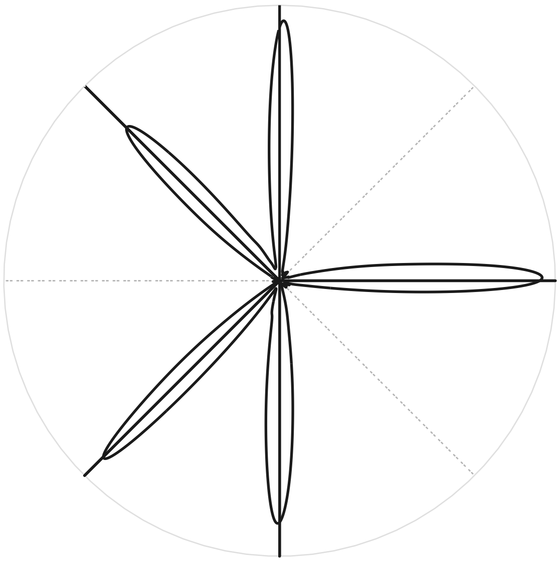

3 2 3 1

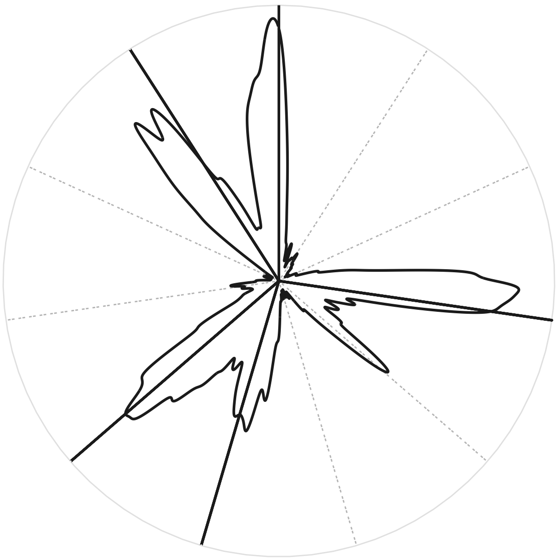

3 2 3 2

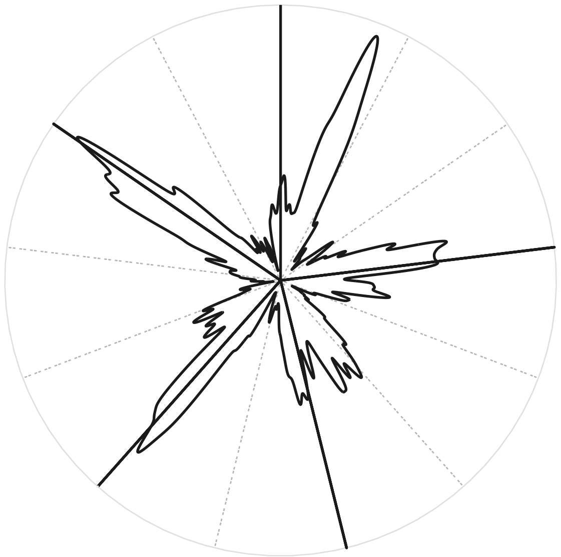

3 4 2 1

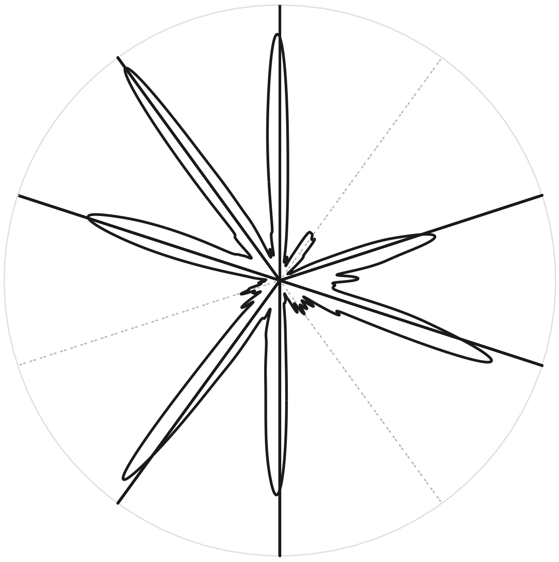

3 4 3 1

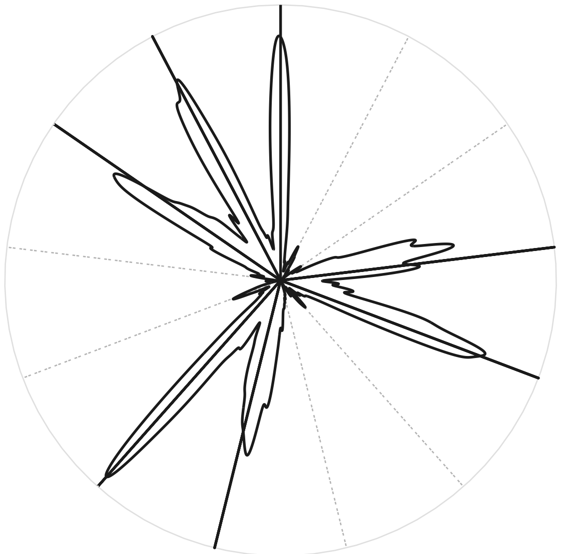

3 5 2 1

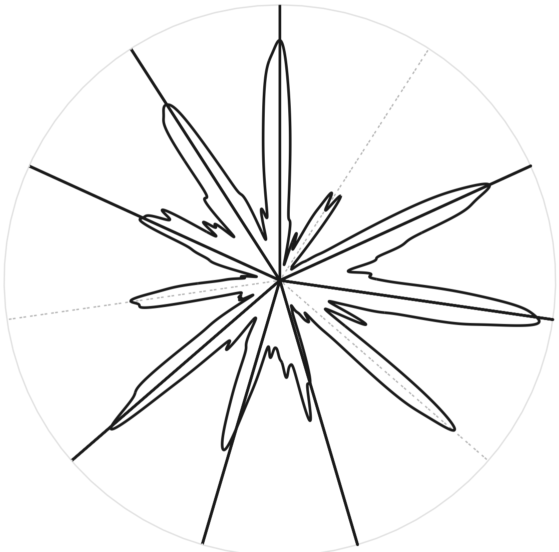

3 7 2 1

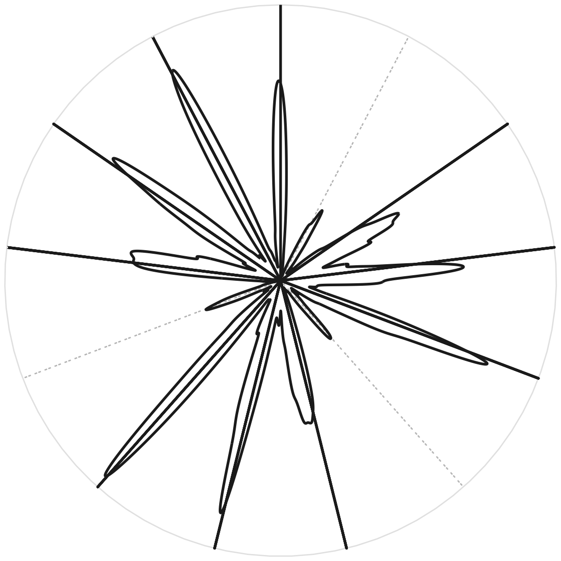

4 1 2 1

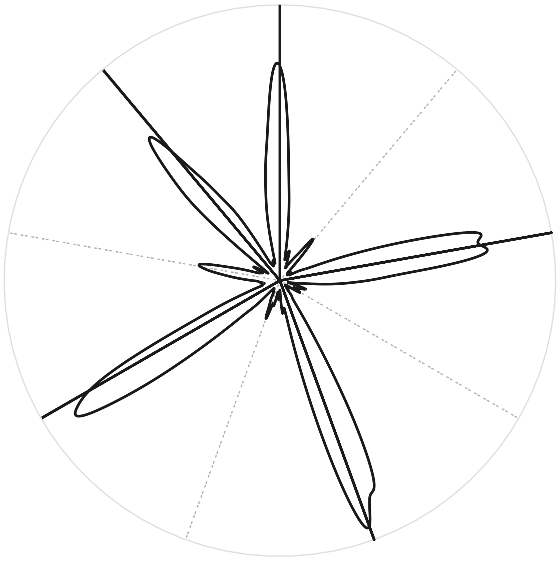

4 1 3 1

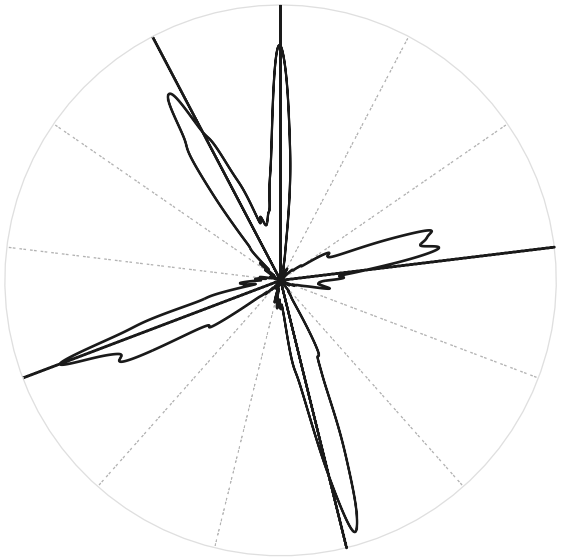

4 3 2 1

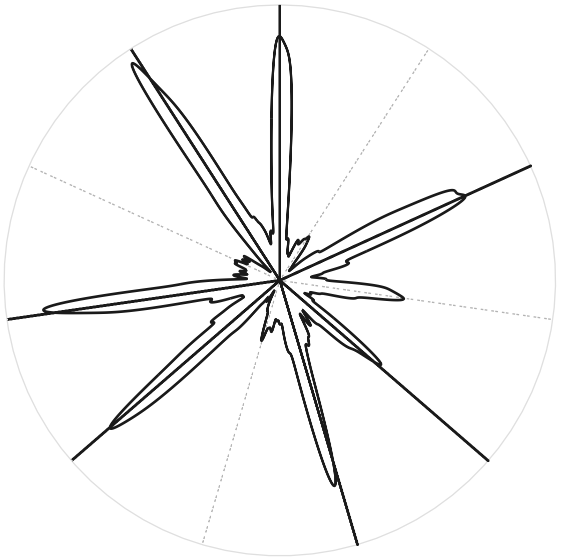

4 5 2 1

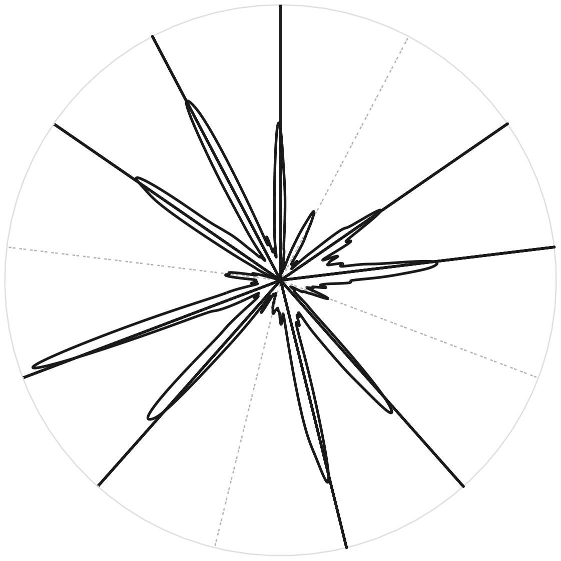

5 1 2 1

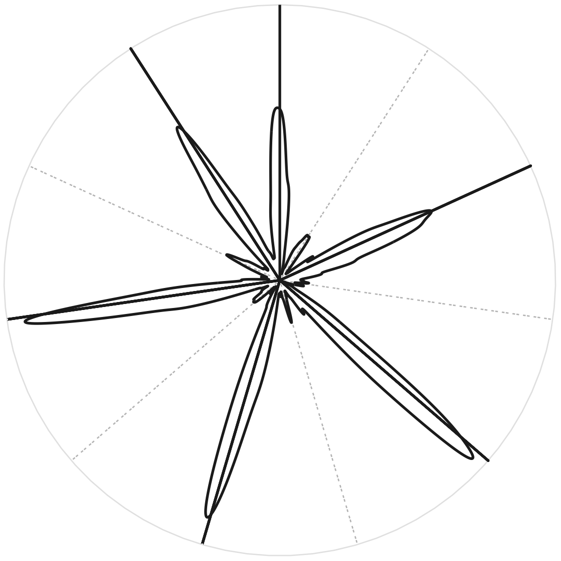

5 2 2 1

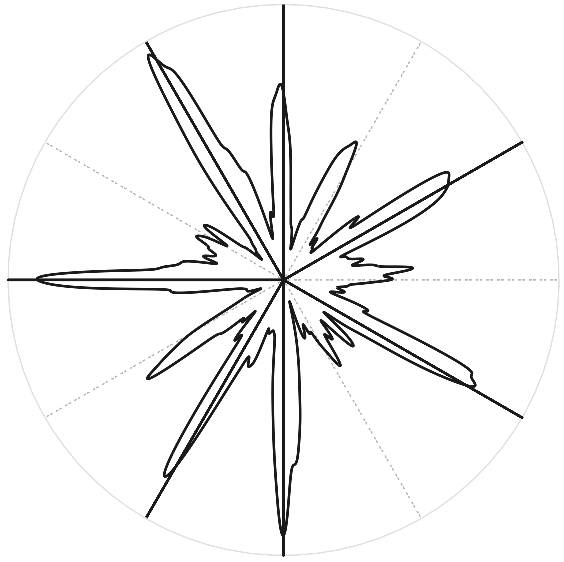

5 3 2 1

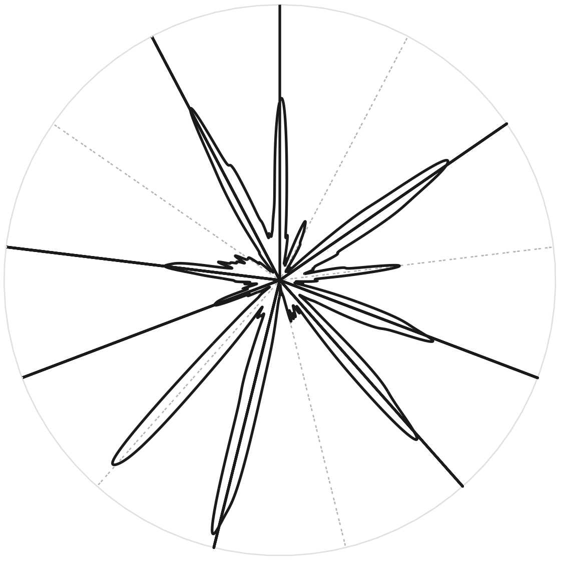

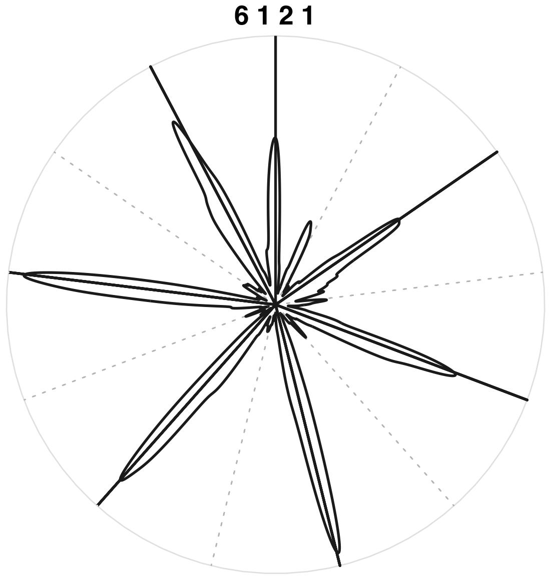
6 1 2 1

S. 8 Performance-level tap accuracy model

Table S5. Full summary of the tap accuracy model.

```
      Family: beta
        Links: mu = logit; phi = identity
     Formula: similarity ~ perf_num + repetition + mean_IOI + evenness + IOI_ent + balance +
duple_triple + (perf_num + repetition + mean_IOI + evenness + IOI_ent + balance + duple_triple |
participant) + (perf_num + repetition | rhythm)
        Data: WF_rhythm_data_perf (Number of observations: 4975)
     Samples: 4 chains, each with iter = 4500; warmup = 2000; thin = 1;
              total post-warmup samples = 10000

     Group-Level Effects:
     ~participant (Number of levels: 111)
                                  Estimate Est.Error l-95% CI u-95% CI Rhat Bulk_ESS Tail_ESS
     sd(Intercept)                    0.38      0.03     0.33     0.43 1.00     1210     2566
     sd(perf_num)                     0.12      0.02     0.08     0.16 1.00     1995     2293
     sd(repetition)                   0.07      0.02     0.02     0.11 1.00     1403     1602
     sd(mean_IOI)                     0.14      0.02     0.12     0.18 1.00     3458     6216
     sd(evenness)                     0.05      0.03     0.00     0.11 1.00     1598     3263
     sd(IOI_ent)                      0.07      0.01     0.05     0.10 1.00     3251     4159
     sd(balance)                      0.10      0.02     0.05     0.14 1.00      804     1666
     sd(duple_triple1)                0.14      0.02     0.09     0.18 1.00     2160     4057
     cor(Intercept,perf_num)         -0.13      0.14    -0.39     0.15 1.00     4267     6330
     cor(Intercept,repetition)        0.40      0.18     0.03     0.73 1.00     4187     3949
     cor(perf_num,repetition)        -0.71      0.18    -0.92    -0.25 1.00     2820     2658
     cor(Intercept,mean_IOI)         -0.20      0.11    -0.42     0.02 1.00     3590     5926
     cor(perf_num,mean_IOI)           0.01      0.16    -0.30     0.31 1.00     1424     3059
     cor(repetition,mean_IOI)        -0.14      0.21    -0.55     0.28 1.02      522     1189
     cor(Intercept,evenness)          0.06      0.28    -0.50     0.59 1.00     7729     7013
     cor(perf_num,evenness)          -0.26      0.31    -0.76     0.41 1.00     4667     6457
     cor(repetition,evenness)         0.24      0.31    -0.43     0.77 1.00     3727     5807
     cor(mean_IOI,evenness)          -0.05      0.29    -0.60     0.52 1.00     8308     7134
     cor(Intercept,IOI_ent)           0.26      0.14    -0.02     0.53 1.00     5909     7126
     cor(perf_num,IOI_ent)            0.13      0.19    -0.25     0.50 1.00     2457     4831
     cor(repetition,IOI_ent)          0.10      0.24    -0.36     0.56 1.00     1197     2764
     cor(mean_IOI,IOI_ent)           -0.20      0.16    -0.50     0.12 1.00     5587     7513
     cor(evenness,IOI_ent)           -0.01      0.30    -0.59     0.58 1.00      923     2563
     cor(Intercept,balance)          -0.18      0.16    -0.50     0.14 1.00     4771     4452
     cor(perf_num,balance)            0.15      0.22    -0.27     0.59 1.00     2516     4134
     cor(repetition,balance)         -0.00      0.25    -0.51     0.48 1.00     1446     3347
     cor(mean_IOI,balance)            0.06      0.19    -0.31     0.43 1.00     5433     6412
     cor(evenness,balance)           -0.20      0.34    -0.75     0.52 1.00      774     2384
     cor(IOI_ent,balance)             0.44      0.18     0.04     0.75 1.00     2421     4134
     cor(Intercept,duple_triple1)     0.42      0.14     0.13     0.69 1.00     3588     4418
     cor(perf_num,duple_triple1)     -0.30      0.18    -0.63     0.07 1.00     2213     5081
     cor(repetition,duple_triple1)    0.30      0.23    -0.17     0.72 1.00      936     2253
     cor(mean_IOI,duple_triple1)     -0.33      0.16    -0.62    -0.01 1.00     4135     5895
     cor(evenness,duple_triple1)      0.15      0.30    -0.46     0.68 1.00     1093     3502
     cor(IOI_ent,duple_triple1)       0.04      0.20    -0.35     0.43 1.00     3157     5420
     cor(balance,duple_triple1)      -0.23      0.21    -0.64     0.20 1.00     2006     3936

     ~rhythm (Number of levels: 91)
                              Estimate Est.Error l-95% CI u-95% CI Rhat Bulk_ESS Tail_ESS
     sd(Intercept)                0.23      0.02     0.19     0.27 1.00     2118     4284
     sd(perf_num)                 0.05      0.02     0.01     0.09 1.00     1629     1781
     sd(repetition)               0.03      0.02     0.00     0.07 1.00     1382     2978
     cor(Intercept,perf_num)     -0.29      0.31    -0.83     0.38 1.00     2802     3995
     cor(Intercept,repetition)   -0.25      0.38    -0.88     0.62 1.00     4923     4736
     cor(perf_num,repetition)    -0.17      0.49    -0.89     0.81 1.00     2633     4809

     Population-Level Effects:
                  Estimate Est.Error l-95% CI u-95% CI Rhat Bulk_ESS Tail_ESS
     Intercept       -1.36      0.05    -1.46    -1.26 1.01      845     1766
     perf_num         0.10      0.02     0.07     0.14 1.00     4181     5641
     repetition      -0.01      0.02    -0.04     0.02 1.00     4783     5524
     mean_IOI        -0.41      0.03    -0.47    -0.35 1.00     1928     3770
     evenness         0.16      0.08     0.01     0.30 1.01     1847     3520
     IOI_ent          0.00      0.03    -0.05     0.06 1.00     1892     3744
     balance         -0.27      0.08    -0.43    -0.11 1.01     1706     3226
     duple_triple1    0.21      0.05     0.10     0.31 1.00     1708     3373

     Family Specific Parameters:
```

```
    Estimate Est.Error l-95% CI u-95% CI Rhat Bulk_ESS Tail_ESS
phi    22.99      0.52    21.98    24.02 1.00     6653     6604

R-squared Values:
                   Estimate Est.Error l-95% CI u-95% CI
Bayes R2               0.67      0.01     0.66     0.68
Cross-validated R2     0.63      0.01     0.61     0.65
```

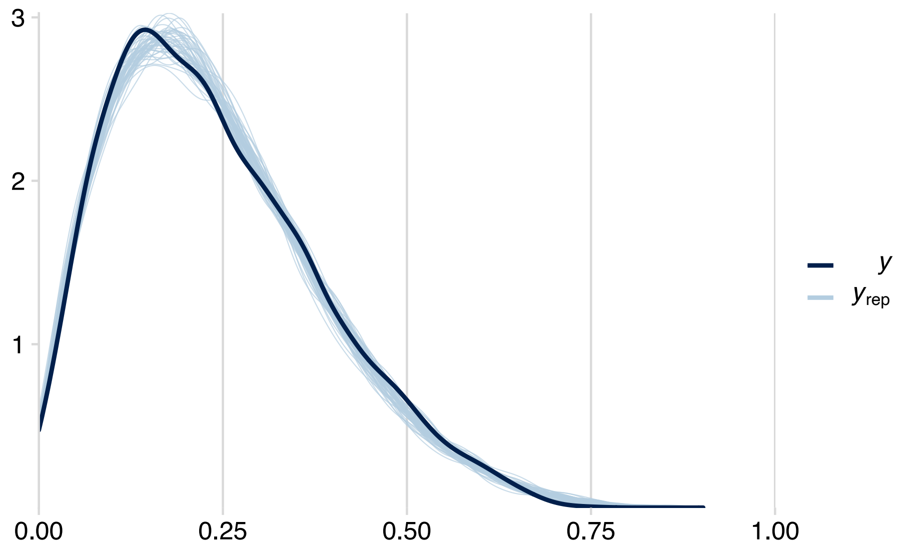

Figure 1. Posterior predictive check for the model shown above: y is the observed data shown as a density plot; y_{rep} shows 50 sets of corresponding predictions from the model.

S. 9 Pulse-level tap probability model

Table S6. Full summary of the tap probability model.

```
      Family: beta_binomial2 
       Links: mu = logit; phi = identity 
     Formula: tap | trials(n_pulses) ~ cue * (tap_lag1 + perf_num + repetition + N + mean_IOI + CQ + 
balance + proj_cent + mean_offset + duple_triple + Markov2 + edge) + (cue | participant) + (cue | 
rhythm) 
        Data: WF_rhythm_data_pulse (Number of observations: 48282) 
     Samples: 4 chains, each with iter = 4500; warmup = 2000; thin = 1;
              total post-warmup samples = 10000

     Group-Level Effects: 
     ~participant (Number of levels: 111) 
                         Estimate Est.Error l-95% CI u-95% CI Rhat Bulk_ESS Tail_ESS
     sd(Intercept)           0.70      0.05     0.61     0.80 1.01      538     1195
     sd(cue1)                0.78      0.05     0.68     0.89 1.01      549     1210
     cor(Intercept,cue1)    -0.89      0.02    -0.93    -0.85 1.00      652     1227

     ~rhythm (Number of levels: 91) 
                         Estimate Est.Error l-95% CI u-95% CI Rhat Bulk_ESS Tail_ESS
     sd(Intercept)           0.64      0.05     0.55     0.75 1.00      806     1258
     sd(cue1)                0.96      0.07     0.82     1.12 1.00      773     1339
     cor(Intercept,cue1)    -0.97      0.01    -0.98    -0.96 1.00     1198     2341

     Population-Level Effects: 
                        Estimate Est.Error l-95% CI u-95% CI Rhat Bulk_ESS Tail_ESS
     Intercept             -1.27      0.12    -1.51    -1.06 1.00      472      917
     cue1                   2.02      0.16     1.71     2.34 1.01      476      930
     tap_lag1              -0.53      0.04    -0.61    -0.46 1.00     2884     4399
     perf_num               0.01      0.02    -0.02     0.05 1.00     2123     4050
     repetition            -0.08      0.02    -0.11    -0.05 1.00     2163     4113
     N                     -0.19      0.08    -0.36    -0.03 1.01      543     1233
     mean_IOI              -0.37      0.08    -0.53    -0.23 1.01      561      890
     CQ                     0.18      0.09     0.01     0.35 1.00      883     1837
     balance                0.12      0.10    -0.08     0.32 1.01      598     1377
     proj_cent              0.45      0.02     0.41     0.48 1.00     3494     5512
     mean_offset           -0.05      0.01    -0.07    -0.02 1.00     3087     4847
     duple_triple1         -0.32      0.15    -0.60    -0.03 1.00      642     1228
     Markov2                0.30      0.01     0.27     0.33 1.00     3836     5868
     edge                   0.43      0.01     0.40     0.45 1.00     3015     5354
     cue1:tap_lag1          0.40      0.05     0.31     0.49 1.00     2984     4779
     cue1:perf_num          0.17      0.02     0.13     0.20 1.00     2152     3981
     cue1:repetition        0.07      0.02     0.04     0.11 1.00     2116     3925
     cue1:N                 0.18      0.13    -0.06     0.42 1.01      527     1320
     cue1:mean_IOI          0.03      0.11    -0.19     0.27 1.01      586      952
     cue1:CQ               -0.24      0.13    -0.49     0.01 1.00      910     1810
     cue1:balance          -0.16      0.15    -0.45     0.13 1.01      588     1220
     cue1:proj_cent        -0.22      0.02    -0.26    -0.18 1.00     3821     5980
     cue1:mean_offset       0.07      0.01     0.05     0.10 1.00     3020     4594
     cue1:duple_triple1     0.46      0.21     0.04     0.88 1.01      635     1227
     cue1:Markov2          -0.27      0.02    -0.31    -0.23 1.00     4371     6070
     cue1:edge             -0.64      0.02    -0.67    -0.61 1.00     3319     5425

     Family Specific Parameters: 
         Estimate Est.Error l-95% CI u-95% CI Rhat Bulk_ESS Tail_ESS
     phi     9.78      0.13     9.54    10.04 1.00     9118     7454

     R-squared Values:
                        Estimate Est.Error l-95% CI u-95% CI
     Bayes R2               0.85      0.00     0.84     0.85
     Cross-validated R2     0.85      0.00     0.85     0.86
```

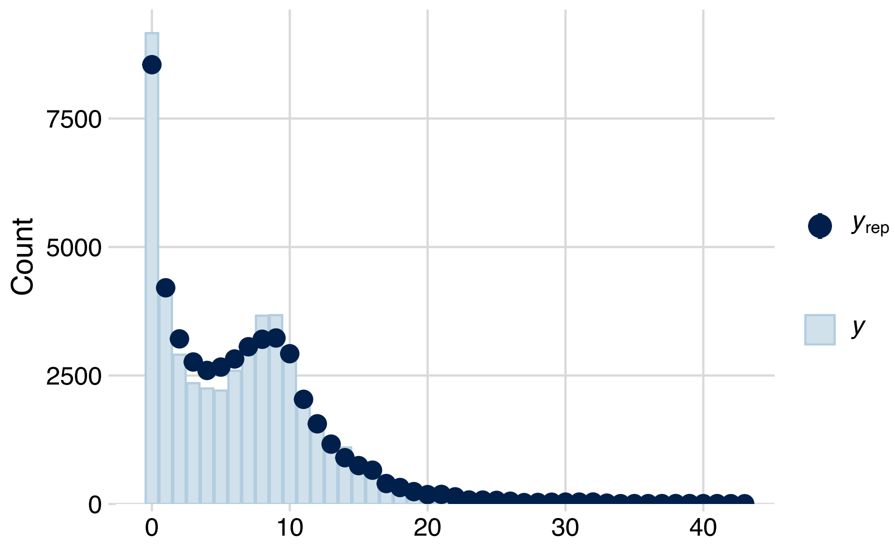

Figure 2. Posterior predictive check for the model shown above: y is the observed data shown as a histogram; y_{rep} shows the 90% intervals obtained from 50 sets of corresponding predictions from the model. The observed data is, for each periodic pulse in each performance, the number of times it was tapped.

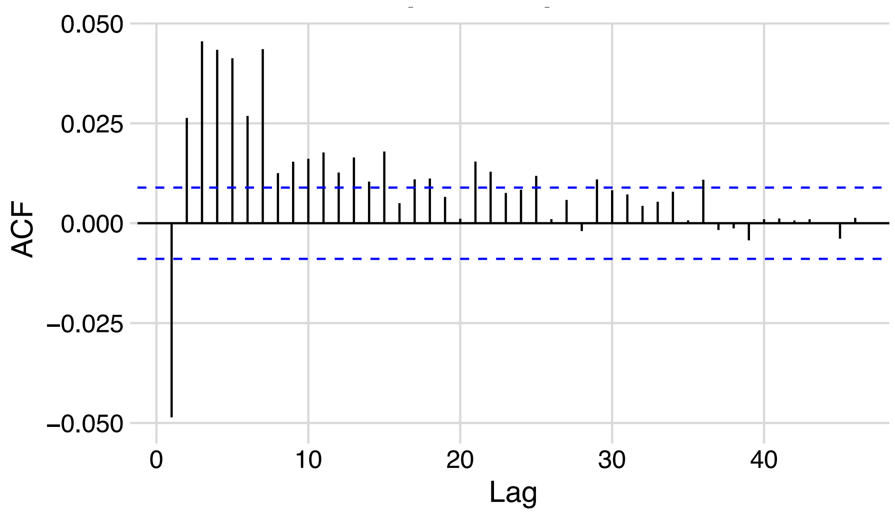

Figure 3. Autocorrelations of the tap probability model's residuals.

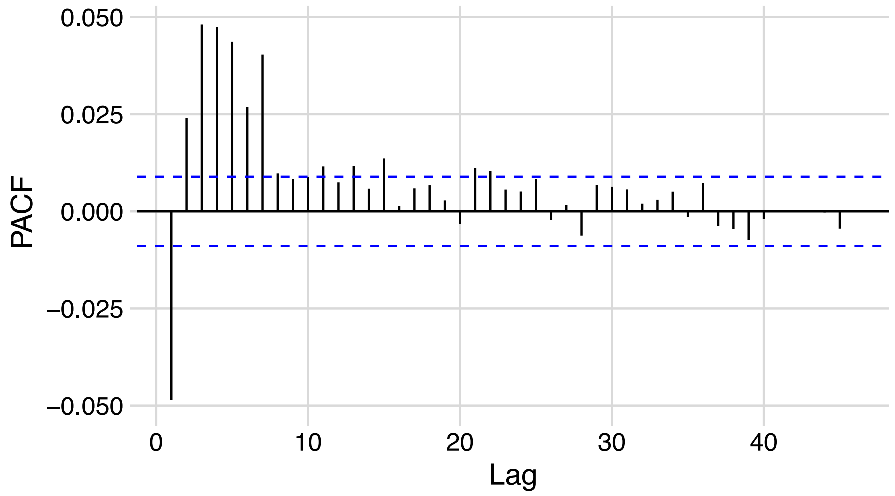

Figure 4. Partial autocorrelations of the tap probability model's residuals.

S. 10 Pulse-level cued pulses with Povel predictors

Table S7. Full summary of the tap probability model for cued pulses substituting Povel's predictors (*iso_accent*, *end_accent*, and *start_accent*) for *edge*.

```
       Family: beta_binomial2 
        Links: mu = logit; phi = identity 
      Formula: tap | trials(n_pulses) ~ tap_lag1 + perf_num + repetition + N + mean_IOI + CQ + balance 
+ proj_cent + mean_offset + duple_triple + Markov2 + iso_accent + end_accent + start_accent + (1 | 
participant) + (1 | rhythm) 
         Data: WF_rhythm_data_pulse_cued (Number of observations: 27425) 
      Samples: 4 chains, each with iter = 4500; warmup = 2000; thin = 1;
               total post-warmup samples = 10000

      Group-Level Effects: 
      ~participant (Number of levels: 111) 
                    Estimate Est.Error l-95% CI u-95% CI Rhat Bulk_ESS Tail_ESS
      sd(Intercept)     0.36      0.03     0.32     0.41 1.00      732     1735

      ~rhythm (Number of levels: 91) 
                    Estimate Est.Error l-95% CI u-95% CI Rhat Bulk_ESS Tail_ESS
      sd(Intercept)     0.38      0.03     0.32     0.44 1.00     1036     1897

      Population-Level Effects: 
                    Estimate Est.Error l-95% CI u-95% CI Rhat Bulk_ESS Tail_ESS
      Intercept         0.86      0.07     0.73     0.99 1.01      652     1249
      tap_lag1         -0.26      0.03    -0.31    -0.21 1.00     4529     6077
      perf_num          0.19      0.01     0.17     0.21 1.00     4203     6054
      repetition       -0.01      0.01    -0.03     0.01 1.00     4233     6224
      N                -0.08      0.05    -0.17     0.02 1.00      737     1347
      mean_IOI         -0.37      0.05    -0.47    -0.28 1.01     1012     2034
      CQ               -0.15      0.05    -0.25    -0.05 1.01     1069     2092
      balance          -0.03      0.06    -0.15     0.08 1.00      984     1771
      proj_cent         0.27      0.01     0.25     0.29 1.00     4000     6273
      mean_offset      -0.05      0.01    -0.07    -0.04 1.00     4448     6405
      duple_triple1     0.16      0.08     0.00     0.33 1.00      774     1562
      Markov2           0.07      0.01     0.04     0.09 1.00     5539     5699
      iso_accent1       0.16      0.03     0.10     0.22 1.00     3370     5424
      end_accent1      -0.08      0.02    -0.12    -0.04 1.00     3936     5671
      start_accent1    -0.68      0.02    -0.73    -0.64 1.00     4849     6164

      Family Specific Parameters: 
          Estimate Est.Error l-95% CI u-95% CI Rhat Bulk_ESS Tail_ESS
      phi    15.57      0.31    14.97    16.18 1.00     5985     6422
```

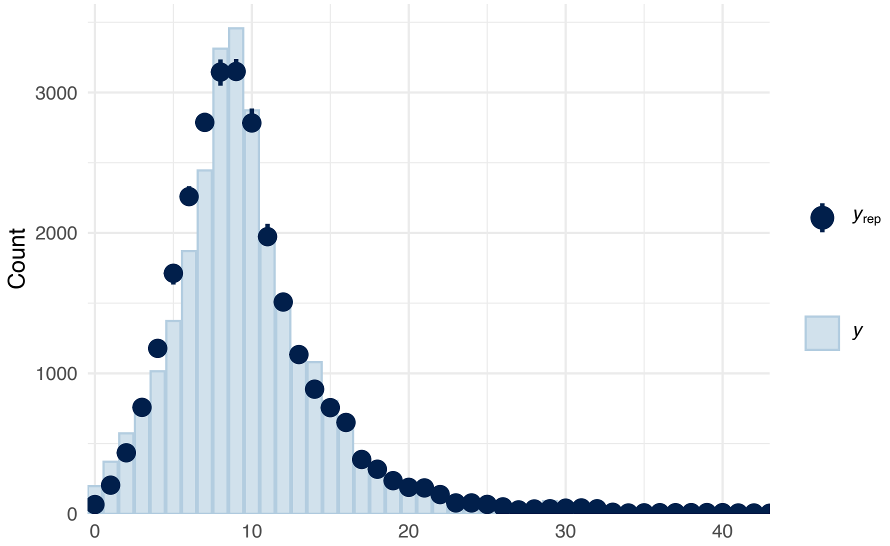

Figure 5. Posterior predictive check for the model shown above: y is the observed data shown as a histogram; y_{rep} shows the 90% intervals obtained from 50 sets of corresponding predictions from the model. The observed data is, for each cued periodic pulse in each performance, the number of times it was tapped. Note that the histogram is different to Figure 2 because this model includes only cued pulses.

S. 11 Pulse-level cued pulses with *edge* predictor

Table S8. Full summary of the tap probability model for cued pulses using *edge* instead of Povel's predictors.

```
      Family: beta_binomial2 
       Links: mu = logit; phi = identity 
     Formula: tap | trials(n_pulses) ~ tap_lag1 + perf_num + repetition + N + mean_IOI + CQ + balance 
+ proj_cent + mean_offset + duple_triple + Markov2 + edge + (1 | participant) + (1 | rhythm) 
        Data: WF_rhythm_data_pulse_cued (Number of observations: 27425) 
     Samples: 4 chains, each with iter = 4500; warmup = 2000; thin = 1;
              total post-warmup samples = 10000

     Group-Level Effects: 
     ~participant (Number of levels: 111) 
                   Estimate Est.Error l-95% CI u-95% CI Rhat Bulk_ESS Tail_ESS
     sd(Intercept)     0.36      0.02     0.31     0.41 1.00      721     1309

     ~rhythm (Number of levels: 91) 
                   Estimate Est.Error l-95% CI u-95% CI Rhat Bulk_ESS Tail_ESS
     sd(Intercept)     0.38      0.03     0.33     0.44 1.00     1254     2402

     Population-Level Effects: 
                   Estimate Est.Error l-95% CI u-95% CI Rhat Bulk_ESS Tail_ESS
     Intercept         0.77      0.07     0.64     0.91 1.01      709     1177
     tap_lag1         -0.14      0.02    -0.18    -0.09 1.00     4152     5614
     perf_num          0.19      0.01     0.17     0.20 1.00     5044     5953
     repetition       -0.01      0.01    -0.03     0.01 1.00     5098     6208
     N                -0.01      0.05    -0.11     0.08 1.00      851     1854
     mean_IOI         -0.34      0.05    -0.43    -0.25 1.00      900     1514
     CQ               -0.07      0.05    -0.17     0.03 1.00     1345     2348
     balance          -0.04      0.06    -0.15     0.08 1.00     1084     1985
     proj_cent         0.24      0.01     0.22     0.26 1.00     5536     6383
     mean_offset       0.03      0.01     0.02     0.04 1.00     5259     5982
     duple_triple1     0.15      0.09    -0.02     0.32 1.01      778     1863
     Markov2           0.03      0.01     0.01     0.05 1.00     5592     6259
     edge             -0.23      0.01    -0.24    -0.21 1.00     5172     6485

     Family Specific Parameters: 
         Estimate Est.Error l-95% CI u-95% CI Rhat Bulk_ESS Tail_ESS
     phi    15.67      0.31    15.07    16.29 1.00     7330     6756
```

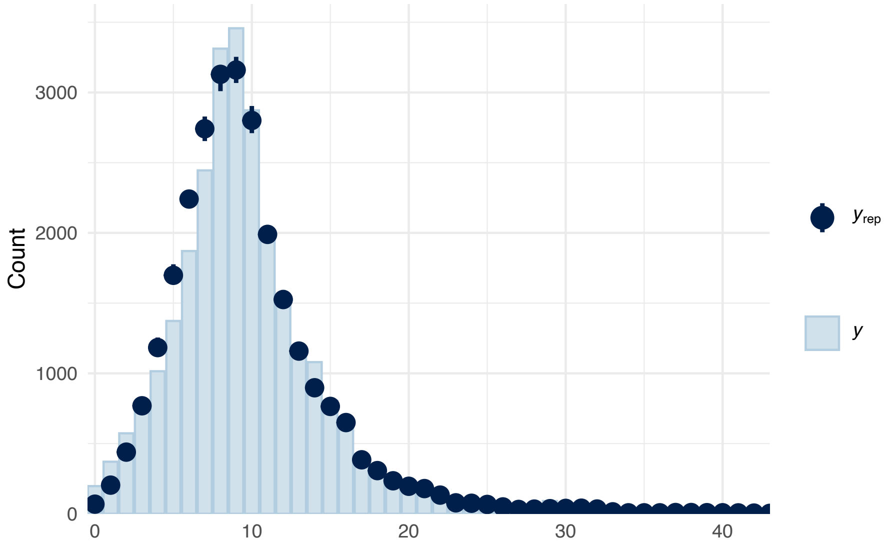

Figure 6. Posterior predictive check for the model shown above: y is the observed data shown as a histogram; y_{rep} shows the 90% intervals obtained from 50 sets of corresponding predictions from the model. The observed data is, for each cued periodic pulse in each performance, the number of times it was tapped. Note that the histogram is different to Figure 2 because this model includes only cued pulses.

S. 12 Pulse-level velocity model

Table S9. Full summary of the tap velocity model.

```
      Family: gaussian
       Links: mu = identity; sigma = identity
     Formula: tap_vel | cens(censoring) ~ cue * (perf_num + duple_triple + seq_exp + Markov2) + (cue |
participant) + (cue | rhythm)
        Data: WF_rhythm_data_tapped (Number of observations: 303903)
     Samples: 4 chains, each with iter = 4500; warmup = 2000; thin = 1;
              total post-warmup samples = 10000

     Group-Level Effects:
     ~participant (Number of levels: 111)
                        Estimate Est.Error l-95% CI u-95% CI Rhat Bulk_ESS Tail_ESS
     sd(Intercept)          0.44      0.03     0.38     0.50 1.01      445     1131
     sd(cue)                0.10      0.01     0.09     0.12 1.00     1720     3228
     cor(Intercept,cue)     0.38      0.09     0.20     0.54 1.00     1228     2518

     ~rhythm (Number of levels: 91)
                        Estimate Est.Error l-95% CI u-95% CI Rhat Bulk_ESS Tail_ESS
     sd(Intercept)          0.06      0.01     0.05     0.07 1.00     2260     3725
     sd(cue)                0.07      0.01     0.05     0.08 1.00     1328     2267
     cor(Intercept,cue)    -0.55      0.08    -0.70    -0.37 1.00     1208     2737

     Population-Level Effects:
                       Estimate Est.Error l-95% CI u-95% CI Rhat Bulk_ESS Tail_ESS
     Intercept             1.29      0.04     1.21     1.37 1.02      219      451
     cue                   0.07      0.01     0.04     0.10 1.01      885     2067
     perf_num              0.12      0.00     0.12     0.13 1.00    16968     8058
     duple_triple1        -0.00      0.01    -0.03     0.02 1.00     1282     2801
     seq_exp              -0.05      0.00    -0.06    -0.05 1.00     5525     7183
     Markov2               0.00      0.00    -0.01     0.01 1.00     6510     7118
     cue:perf_num          0.00      0.00    -0.00     0.01 1.00    17577     7862
     cue:duple_triple1     0.02      0.01    -0.01     0.05 1.00     1162     1987
     cue:seq_exp           0.02      0.00     0.01     0.02 1.00     5462     6607
     cue:Markov2          -0.02      0.00    -0.02    -0.01 1.00     6974     7822

     Family Specific Parameters:
           Estimate Est.Error l-95% CI u-95% CI Rhat Bulk_ESS Tail_ESS
     sigma     0.39      0.00     0.39     0.39 1.00    15565     6920

     R-squared Values:
                        Estimate Est.Error l-95% CI u-95% CI
     Bayes R2               0.62      0.00     0.62     0.62
     Cross-validated R2     0.58      0.00     0.58     0.58
```

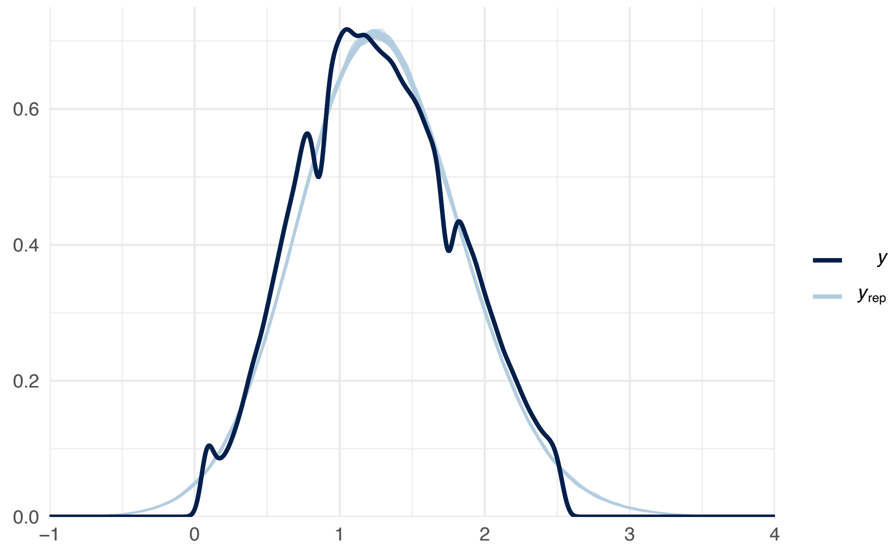

Figure 7. Posterior predictive check for the censored model shown above: y is the observed data shown as a density plot; y_{rep} shows 50 sets of corresponding predictions from the model.

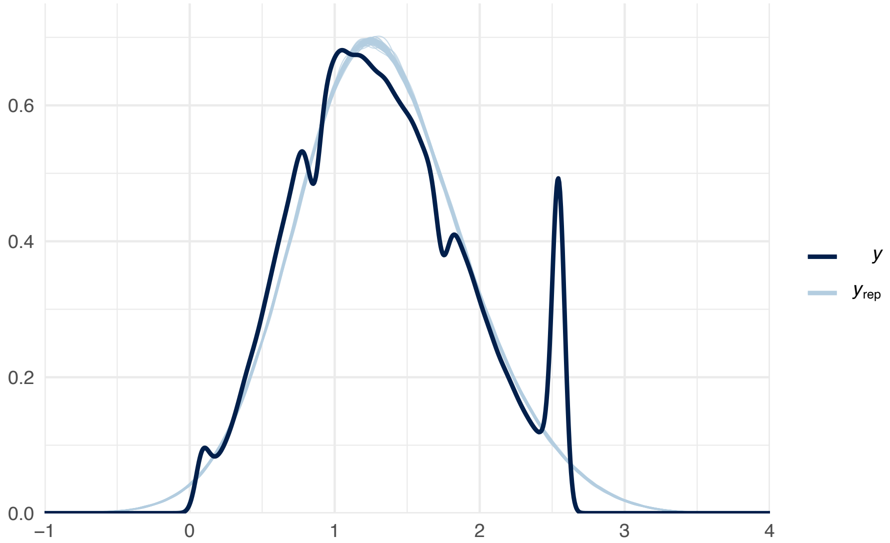

Figure 8. For comparison, this is the posterior predictive check obtained when censoring is not applied: y is the observed data shown as a density plot; y_{rep} shows 50 sets of corresponding predictions from the model. The peak in the data at the right results from the cluster of values at the maximum MIDI velocity value of 127. Many of these taps at 127 would have been harder than the minimum required to reach this velocity value. Note how in the above plot from the censored model, these taps are no longer modelled as being at 127; rather they are modelled as if they represent values ≥ 127, and so are no longer directly depicted in the plot.

S. 13 Pulse-level tap-timing error model

Table S10. Full summary of the tap timing error model.

```
      Family: gaussian
       Links: mu = identity; sigma = identity
     Formula: tap_delta | trunc(lb = -2.372128, ub = 2.372128) ~ cue * (perf_num + mean_offset +
mean_IOI + evenness + Markov2 + edge) + (cue | participant) + (cue | rhythm)
        Data: WF_rhythm_data_tapped (Number of observations: 303903)
     Samples: 4 chains, each with iter = 4500; warmup = 2000; thin = 1;
              total post-warmup samples = 10000

     Group-Level Effects:
     ~participant (Number of levels: 111)
                        Estimate Est.Error l-95% CI u-95% CI Rhat Bulk_ESS Tail_ESS
     sd(Intercept)          0.19      0.02     0.16     0.22 1.00     3014     4493
     sd(cue)                0.29      0.02     0.25     0.33 1.00     1137     2469
     cor(Intercept,cue)    -0.80      0.04    -0.86    -0.71 1.00     1302     2532

     ~rhythm (Number of levels: 91)
                        Estimate Est.Error l-95% CI u-95% CI Rhat Bulk_ESS Tail_ESS
     sd(Intercept)          0.30      0.02     0.25     0.35 1.00     1974     3642
     sd(cue)                0.39      0.03     0.33     0.45 1.00     1845     3953
     cor(Intercept,cue)    -0.95      0.01    -0.97    -0.92 1.00     1962     3911

     Population-Level Effects:
                     Estimate Est.Error l-95% CI u-95% CI Rhat Bulk_ESS Tail_ESS
     Intercept           0.10      0.04     0.02     0.17 1.00     1381     2679
     cue                -0.28      0.05    -0.38    -0.18 1.00     1344     2392
     perf_num            0.04      0.01     0.03     0.05 1.00     9794     7037
     mean_offset        -0.38      0.01    -0.39    -0.37 1.00     9740     6969
     mean_IOI           -0.07      0.03    -0.13     0.00 1.00     1406     2081
     evenness           -0.11      0.03    -0.17    -0.05 1.00     1574     2895
     Markov2             0.13      0.01     0.11     0.15 1.00     7714     7333
     edge                0.08      0.01     0.06     0.10 1.00     5073     6655
     cue:perf_num       -0.03      0.01    -0.04    -0.02 1.00     9904     7301
     cue:mean_offset     0.36      0.01     0.35     0.38 1.00     9866     6177
     cue:mean_IOI       -0.04      0.04    -0.12     0.04 1.00     1451     2082
     cue:evenness        0.14      0.04     0.05     0.22 1.00     1564     2902
     cue:Markov2         0.01      0.01    -0.02     0.03 1.00     7901     7413
     cue:edge           -0.05      0.01    -0.07    -0.03 1.00     5297     6569

     Family Specific Parameters:
           Estimate Est.Error l-95% CI u-95% CI Rhat Bulk_ESS Tail_ESS
     sigma     1.06      0.00     1.05     1.06 1.00    13699     6415

     R-squared Values:
                        Estimate Est.Error l-95% CI u-95% CI
     Bayes R2               0.06      0.00     0.06     0.07
     Cross-validated R2     0.07      0.00     0.06     0.07
```

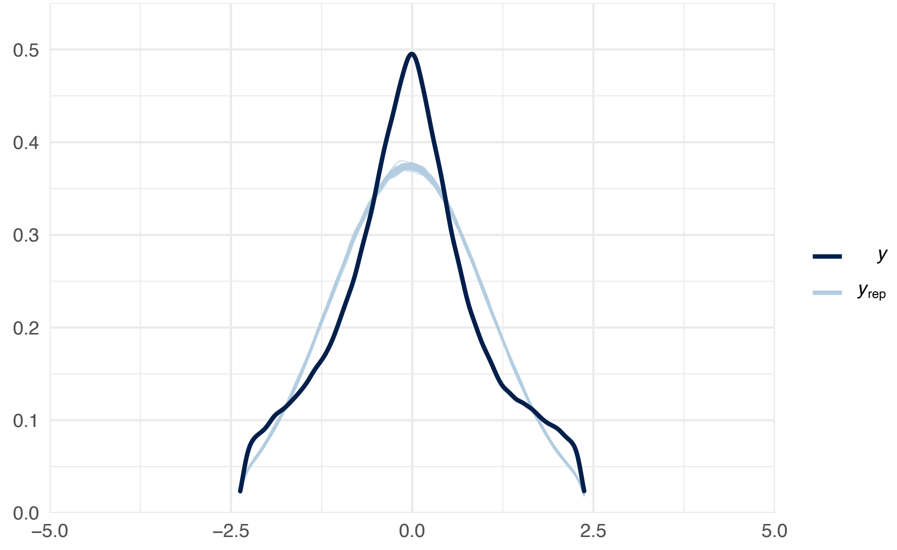

Figure 9. Posterior predictive check for the censored model shown above: y is the observed data shown as a density plot; y_{rep} shows 50 sets of corresponding predictions from the model.

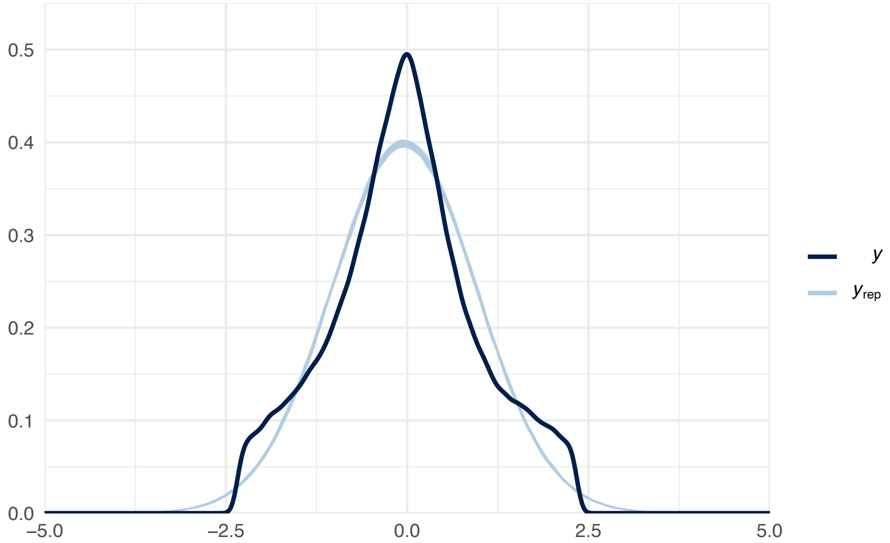

Figure 10. For comparison, this is the posterior predictive check obtained when truncation is not applied: y is the observed data shown as a density plot; y_{rep} shows 50 sets of corresponding predictions from the model. It is worth noting that there is some misfit in both the truncated and untruncated versions – prior to the truncation, the tails are very heavy. A *student-t* distribution was attempted, but fitted worse.
